# Cost-consequence analysis of an e-health intervention to reduce distress in dementia carers: results from the iSupport randomised controlled trial

**DOI:** 10.1136/bmjopen-2024-095611

**Published:** 2025-05-16

**Authors:** Bethany Anthony, Kodchawan Doungsong, Catherine MacLeod, Greg Flynn, Patricia Masterson-Algar, Nia Goulden, Kieren Egan, Kiara Jackson, Suman Kurana, Gwenllian Hughes, Ryan Innes, John Connaghan, Danielle Proctor, Fatene Abakar Ismail, Zoe Hoare, Aimee Spector, Joshua Stott, Gill Windle, Rhiannon Tudor Edwards

**Affiliations:** 1 Centre for Health Economics and Medicines Evaluation (CHEME), Bangor University, Bangor, UK; 2 Centre for Population Health Sciences, Usher Institute, The University of Edinburgh, Edinburgh, UK; 3 North Wales Organisation for Randomised Trials in Health and Social Care (NWORTH), Bangor University, Bangor, UK; 4 Dementia Services Development Centre Wales (DSDC Wales), Bangor University, Bangor, UK; 5 Department of Computer and Information Science, University of Strathclyde, Glasgow, UK; 6 Department of Clinical, Educational and Health Psychology, University College London, London, UK

**Keywords:** Dementia, Caregivers, Health Care Costs, HEALTH ECONOMICS

## Abstract

**Objective:**

The use of e-health interventions has grown in demand due to their accessibility, low implementation costs and their potential to improve the health and well-being of people across a large geographical area. Despite these potential benefits, little is known about the cost-effectiveness of self-guided e-health interventions. The aim of the study was to compare the cost and consequences of ‘iSupport’, an e-health intervention to reduce mental health issues in dementia carers.

**Design:**

A cost-consequence analysis (CCA) of a multi-centre, single-blind randomised controlled trial of iSupport. The CCA was conducted from a public sector (National Health Service, social care and local authority) perspective plus a wider societal perspective. Delivery costs of iSupport were collected using a bottom-up micro-costing approach.

**Setting:**

352 participants were recruited from three centres in England, Wales and Scotland.

**Participants:**

Participants eligible for inclusion were adults over the age of 18 years who self-identified as an unpaid carer with at least 6 months of experience caring for an individual with a diagnosis of dementia. Between 12 November 2021 and 31 March 2023, 2332 carers were invited to take part in the study. 352 participants were randomised: 175 randomised to the iSupport intervention group and 177 to the usual care control group. The mean age of participants in the intervention and control groups was 63 and 62, respectively.

**Main outcome measures:**

The CCA presented the disaggregated costs and health-related quality of life measured using the EuroQol five-dimension.

**Results:**

There was no significant difference in generic health-related quality of life measured using the EQ-5D-5L (p=0.67). Both groups reported higher mean costs between baseline and 6 months, but the change in costs was significantly lower in the intervention group. Between baseline and 6 months, the mean change in total resource use costs from the public sector perspective was significantly different between groups (p=0.003, r=−0.161) reporting a mean change per participant of £146 (95% CI: −33 to 342) between the intervention and control groups. From the wider societal perspective, there was no significant difference (p=0.23) in the mean change in total resource use and informal care costs between the two groups from baseline to 6 months.

**Conclusion:**

Use of iSupport was associated with reduced health and social care resource use costs for carers compared with care-as-usual. Self-guided e-health interventions for dementia carers may have the potential to reduce health and social care resource use and wider societal costs, but evidence relating to their effectiveness and cost-effectiveness is lacking.

**Trial registration number:**

ISRCTN17420703.

**STRENGTHS AND LIMITATIONS OF THIS STUDY**

- This study provided detailed costings of health and social care services utilised by people living with dementia and their carers.
- The analysis accounted for hours spent by carers performing informal care tasks; however, the accurate measurement of informal care costs to acknowledge its full value to society presents particular challenges.
- This study relied on participants’ estimation of hours spent on completing caregiving tasks, which can often be difficult for caregivers to accurately recall.
- Costs were compared at 3 months and 6 months; thus, the longer-term impact on costs is not accounted for in this study.

## Introduction

 In the UK, there are over 800 000 people living with dementia, and this figure is expected to rise by 80% by 2040.^1^ The annual cost of dementia is over £34 billion,^2^ and unpaid care accounts for 40% of this cost, which represents the estimated economic value if the equivalent amount of care was provided by formal (paid) carers.^1^ Caring for people living with dementia can have negative impacts on carers’ physical and mental health.^3^ E-health interventions are increasingly being promoted globally as an option to support informal carers, reduce carer strain and promote resilience. Further potential benefits include their scalability, anonymous delivery mode and reduced costs.

We searched Medline, Embase, the Cochrane Library and the NIHR Centre for Reviews and Dissemination for peer-reviewed economic evaluation articles in English published between 1 Jan 2012 and 17 Nov 2022, with keywords ‘e-health’, ‘self-guided’, ‘economic evaluation’ and ‘cost-effectiveness analysis’. Eleven economic evaluations of self-guided or minimally guided e-health interventions were identified. Self-guided or minimally guided e-health interventions have been shown to be cost-effective for mental health outcomes,^4^,^6^ diabetes management,^7^ urinary incontinence,^8^ parental education,^9^ physical activity^10^ and back pain.^11^ Conversely, other studies have not demonstrated cost-effectiveness findings of self-guided or minimally guided e-health interventions for ankle sprains,^12^ eating disorders^13^ and the management of symptoms among cancer patients.^14^ Our literature searches did not identify any evidence on the cost-effectiveness of self-guided or minimally guided e-health interventions targeting people living with dementia or their carers.

‘iSupport for dementia carers’ is an online self-guided psychoeducation, skills and self-care programme for carers of people living with dementia, developed by experts at the WHO.^15^ In response to UK National Health Service (NHS) recommendations promoting training for informal carers of people living with dementia, ‘iSupport’ aims to equip carers with necessary skills to provide effective care and manage their own physical and mental health.

The iSupport randomised controlled trial (RCT) found no significant between-group differences in either primary outcome: carer burden (12-item Zarit Burden Interview, ZBI-12) and depression (10-item Centre for Epidemiological Studies of Depression Scale, CES-D-10) at 3 months and 6 months.^16^ There were also no significant between-group differences in health-related quality of life (DEMQOL-Proxy) at 3 months and 6 months. Given the null results from the RCT, we present a cost-consequence analysis (CCA) of the iSupport intervention, deviating from the original protocol which planned for a cost-effectiveness analysis. The aim of this study was to compare the costs and consequences of iSupport relative to care as usual (control).

## Methods

iSupport was a multi-centre, pragmatic, single-blinded RCT conducted between January 2021 and December 2023.^17^ Carer participants living in England, Wales and Scotland were recruited at the following centres: UCL, England; University of Strathclyde, Scotland; and Bangor University, Wales. Recruitment was facilitated through advertisements by the study partners (Alzheimer Scotland and Carers Trust Wales), the UK Join Dementia Research (JDR) register and two NHS health boards. The study was also advertised over social media. Participants eligible for inclusion were adults over the age of 18 years who self-identified as an unpaid carer with at least 6 months of experience caring for an individual with a diagnosis of dementia. Participants were eligible if they self-reported feelings of stress, depression or anxiety. Participants in receipt of mental health specialist delivered psychological therapy during the time of recruitment were not eligible to participate in the trial. All participants provide their informed consent to take part in the study. Further details of participants’ eligibility and recruitment are available in the main RCT protocol paper.^17^

Ethical approval was granted by Bangor University’s School of Health and Medical Sciences Academic Ethics Committee (reference: 2021–16915) and the Health Research Authority (IRAS project number: 311,565) via the London—City & East Research Ethics Committee (reference 22/LO/0688). A UKCRC-registered clinical trials unit, a trial management group, an independent trial steering committee and data monitoring committee provided oversight of the trial. The published protocol is available elsewhere.^17^

### Patient and public involvement

We involved people living with dementia and their carers in the design, conduct and dissemination of this project, as reported elsewhere.^16 17^

### Procedure and data collection

Participants were randomly allocated on a 1:1 ratio to iSupport or care as usual (control). iSupport is an interactive e-health platform that was developed by the WHO to provide online training and support to dementia carers. The iSupport intervention consisted of five themes (introduction to dementia, being a carer, caring for me, providing everyday care and dealing with behaviour changes) and 23 accompanying exercises. Each exercise lasted between 5 and 15 min and had read-aloud introductions. iSupport allowed carers to create a personalised plan and select sessions most relevant to them. Further details of the iSupport intervention are available in the protocol paper.^17^

### Intervention

Following randomisation, participants in the intervention group were provided with log-in details and a short guide on how to access the iSupport platform. To maximise the benefits, participants were advised to use the intervention on a regular basis with the availability of technical support provided by an e-coach. This included sending invitation emails and ‘check-in’ emails to ‘inactive’ participants who had not accessed the iSupport platform or who had only accessed the platform for a short duration (eg, accessed a module but did not complete it or log in again). Additional e-coach delivery tasks included responding to participant queries, providing telephone support, and monthly inactivity checks of the iSupport platform, as reported elsewhere.^18^

### Control

Participants in the control group received care-as-usual in the form of a PDF or printed booklet about dementia developed by the Alzheimer’s Society.^2^ Participants in the control group also received some correspondence via e-mail with the e-coach to be a point of contact for the study and to check that they had received their PDF booklet by email.

### Data collection

Data were collected from carer participants at baseline (before randomisation) and 3-month and 6-month follow-ups by trained research assistants via online video conference or telephone calls. A bespoke client service receipt inventory was used to collect frequencies of health and social care resource use for hospital and community-based services, self-reported by carers.

Health-related quality of life was assessed using the EQ-5D-5L, a five-domain quality of life measure encompassing mobility, self-care, usual activities, pain/discomfort and anxiety/depression.^19^ Participants scored each item on a scale of 1–5, where 1 indicates no problems in that domain and 5 indicates extreme problems/unable to perform.

### Perspective of analysis

A CCA of iSupport was conducted from a public sector (NHS, social care and local authority) perspective plus a wider societal perspective. CCA is particularly useful for allowing decision-makers to weight the importance of different costs and outcomes according to their importance in the decision-making context.^20^ The base-case analysis was conducted from a public sector perspective comprising resource use costs borne by the NHS, personal social services and local authorities. The secondary analysis adopted a wider societal perspective, considering privately funded (paid for by patients/families) and third sector (eg, charities) resource use and the opportunity costs of informal care. The analysis is reported according to the Consolidated Health Economic Evaluation Reporting Standards (CHEERS) guidelines.^21^ The CHEERS checklist is available in online supplemental file 1. The health economics analysis plan and the iSupport protocol are available in onlinesupplemental files 2  3, respectively.

#### Intervention delivery costs

**Figure 1:**
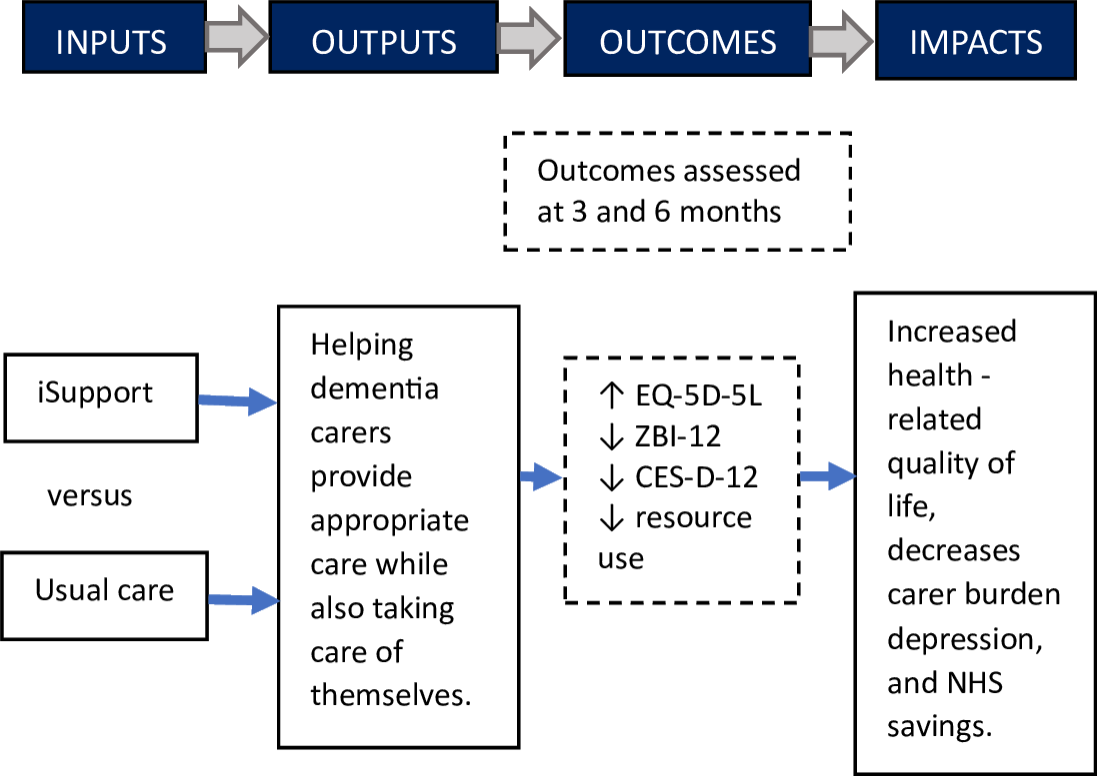
An economic evaluation logic model of the iSupport trial. EQ-5D-5L, EuroQol- 5-Dimensions 5-Levels; ZBI-12, 12-item Zarit Burden Interview; CES-D-12, 10-item Centre for Epidemiological Studies of Depression Scale; NHS, National Health Service

A bottom-up micro-costing approach was conducted to calculate iSupport delivery costs relative to costs incurred by the control group. Each resource use component is collected and then combined with its unit cost to allow accurate programme costing.^22^ iSupport delivery costs comprised e-coach time providing technical support and supporting carers to use the tool. Costs incurred by the control group included e-coach time to send out emails and material costs for delivering paper copies (n=39) of the Alzheimer’s Society booklet (online supplemental file 4, Table 1). E-coach time was collected via a cost information spreadsheet detailing the frequency of tasks performed and the average time (minutes) to complete each discrete task. E-coach time was costed using salary information including on-costs. Total costs for each task were calculated by multiplying the frequency of tasks by the average time to complete each task. Upfront intervention development costs and training are presented separately and do not come under total costs of intervention delivery. The logic model of the iSupport intervention presents how the intervention intended to increase health-related quality of life and decrease carer burden, depression and health service resource use (figure 1).

**Table 1:** Baseline characteristics of participants (n=352)

Control group (n=177)		Control groupn (%)	Intervention groupn (%)
Intervention group (n=175)			
Age (mean, ±SD)		62 (11.3)	63 (12.0)
Gender	
Male	36 (20.3%)	35 (20.0%)
Female	140 (79.1%)	137 (78.3%)
Other	0 (0%)	0 (0%)
No answer	0 (0%)	0 (0%)
Missing data	1 (0.6%)	3 (1.7%)
Main language	
English	175 (98.9%)	172 (98.3%)
Welsh	0 (0%)	2 (1.1%)
Gaelic	0 (0%)	0 (0%)
Other	2 (1.2%)	1 (0.6%)
Ethnicity	
English/Welsh/Scottish/Northern Irish/British	164 (92.7%)	164 (93.7%)
Irish	0 (0%)	1 (0.6%)
Any other White background	4 (2.3%)	3 (1.7%)
Indian	2 (1.1%)	1 (0.6%)
Chinese	1 (0.6%)	0 (0%)
Any other Asian background	0 (0%)	1 (0.6%)
White and Asian	2 (1.1%)	0 (0%)
Any other mixed/multiple ethnic background	2 (1.1%)	0 (0%)
Caribbean	1 (0.6%)	3 (1.7%)
Any other Black/African/Caribbean background	0 (0%)	1 (0.6%)
Any other ethnic group	0 (0%)	1 (0.6%)
Prefer not to say	1 (0.6%)	0 (0%)
Highest education	
University higher degree	47 (26.6%)	39 (22.3%)
First-degree level qualification	46 (26.0%)	62 (35.4%)
Apprenticeship	0 (0%)	4 (2.3%)
Degree level	27 (15.3%)	21 (12.0%)
A level	14 (7.9%)	13 (7.4%)
NVQ level 3 or below	2 (1.1%)	6 (3.4%)
CSEs, GCSE/O level	21 (11.9%)	17 (9.7%)
Any other qualification	17 (9.6%)	11 (6.3%)
None of the above	3 (1.7%)	2 (1.1%)
Occupational status	
Professional	96 (55%)	90 (52%)
Managerial/technical	98 (22%)	35 (20%)
Skilled (non-manual)	34 (19%)	40 (23%)
Skilled (manual)	4 (2.3%)	2 (1.2%)
Partly skilled	2 (1%)	4 (2%)
Unskilled	1 (0.6%)	2 (1%)
Have paid work	
No	12 (6.8%)	12 (6.9%)
No (retired)	94 (53.1%)	99 (56.5%)
No (seeking work)	2 (1.1%)	1 (0.6%)
No (long-term sick or disabled)	2 (1.1%)	1 (0.6%)
Yes, full-time (a contract of 36 hours or more per week)	34 (19.2%)	26 (14.8%)
Yes, part-time	33 (18.6%)	35 (20.0%)
Missing data	0 (0%)	1 (0.6%)
Marital status	
Single and never married or never in a legally recognised civil partnership	18 (10.2%)	18 (10.3%)
Married	132 (74.6%)	130 (74.3%)
Civil partnership in a legally recognised civil partnership	6 (3.4%)	3 (1.7%)
Separate but legally married	0 (0%)	2 (1.1%)
Divorced	5 (2.8%)	8 (4.6%)
Windowed	4 (2.3%)	1 (0.6%)
Cohabiting	12 (6.8%)	13 (7.4%)
Relationship with person with dementia	
Spouse/partner	75 (42.4%)	77 (44.0%)
Sibling	3 (1.7%)	2 (1.1%)
Child	90 (50.8%)	87 (49.7%)
Parent	0 (0%)	0 (0%)
Friend	2 (1.1%)	1 (0.6%)
Other	7 (4.0%)	8 (4.6%)
Type of dementia	
Alzheimer’s disease	80 (45.2%)	80 (45.7%)
Vascular dementia	26 (14.7%)	28 (16.0%)
Familial Alzheimer’s disease	0 (0%)	1 (0.6%)
Frontotemporal dementia	5 (2.8%)	4 (2.3%)
Primary progressive aphasia	1 (0.6%)	0 (0%)
Posterior cortical atrophy	1 (0.6%)	1 (0.6%)
Dementia with Lewy bodies	4 (2.3%)	5 (2.9%)
Other	47 (26.6%)	46 (26.3%)
Don’t know	13 (7.3%)	10 (5.7%)
Travel distance from person with dementia	
Mean (minutes, ±SD)		24 (76.5)	26 (71.4)
Median (IQR)		1.5 (15)	0 (20)
Experience in providing informal care (years, ±SD)		3.9 (2.88)	4.3 (4.5)

#### Resource use and informal care costs

Within our base case analysis, hospital and community-based resource use were costed from the public sector perspective (ie, resource use funded by the NHS, personal social services and local authorities). Hospital and community-based resource use funded privately (by families/patients) or third-sector organisations (eg, charities) were costed within our wider societal perspective of analysis.

Health and social care resource use costs were also collected for both carers in the trial and the person they care for. Resource use data was costed in British pounds sterling (£) for cost year 2021/22 using national unit costs.^23 24^ Total resource use costs were calculated by multiplying unit cost per item by reported number of times each resource was used. For inpatient stays, cost per spell was applied to each hospital resource group (HRG) inpatient stay and an excess bed day charge was applied to any stays exceeding the HRG trim point. No discounting was conducted as the trial follow-up period was less than 1 year. Missing resource use data was not imputed in this analysis. Due to the non-normality of the cost data, non-parametric independent Mann-Whitney U tests were used to compare the difference in mean costs between control and intervention groups at a p value significance level of 0.05. The unit cost schedule of resource use items and costing assumptions is available in online supplemental file 4, Tables 2 and 3.

**Table 2:** Difference in mean costs per participant from the public sector perspective and wider societal perspective (£, 95% CI)

	Control group			Intervention group			Mean change at 6 months between groups (Intervention and control)	P value
	Baseline (n=177)	3 months (n=140)	6 months (n=137)	Baseline (n=175)	3 months (n=129)	6 months (n=126)		
Mean cost of community services (public sector)	£68 (56 to 81)	£147 (112 to 173)	£167 (134 to 205)	£102 (81 to 125)	£163 (122 to 207)	£134 (102 to 168)	−£82 (32 to 132)	0.003*
Mean cost of hospital services (public sector)	£99 (60 to 146)	£109 (39 to 207)	£194 (97 to 318)	£176 (76 to 327)	£161 (71 to 262)	£159 (79 to 259)	−£64 (−96 to 238)	0.57
Mean total resource use (public sector)	£168 (124 to 217)	£247 (165 to 358)	£371 (255 to 516)	£278 (168 to 441)	£326 (220 to 449)	£292 (195 to 416)	−£146 (−33 to 342)	0.003*
Mean cost of community services (societal perspective)	£70 (57 to 83)	£146 (152 to 230)	£189 (153 to 228)	£111 (88 to 135)	£167 (127 to 211)	£140 (104 to 179)	−£98 (47 to 148)	<0.001*
Mean cost of hospital services (societal perspective)	£99 (60 to 146)	£109 (39 to 210)	£194 (97 to 318)	£178 (77 to 328)	£162 (76 to 265)	£159 (79 to 259)	−£64 (−96 to 238)	0.57
Mean informal care costs: Proxy good method (societal perspective)	£461 (404 to 522)	£424 (368 to 485)	£431 (369 to 498)	£483 (417 to 557)	£413 (345 to 487)	£483 (403 to 567)	£64 (−169 to 43)	0.041*
Mean total resource use and informal care costs (societal perspective)	£617 (544 to 694)	£668 (566 to 785)	£785 (643 to 953)	£771 (627 to 967)	£738 (611 to 881)	£771 (641 to 911)	−£81 (−126 to 302)	0.23
Mean informal care costs following sensitivity analysis (societal perspective)	£413 (368 to 459)	£401 (349 to 457)	£383 (336 to 433)	£415 (369 to 463)	£369 (315 to 425)	£396 (344 to 450)	£59 (−132 to 13)	0.048*
Mean total resource use and informal care following sensitivity analysis (societal perspective)	£570 (508 to 635)	£645 (547 to 758)	£740 (607 to 900)	£703 (580 to 875)	£694 (575 to 833)	£685 (574 to 811)	−£86 (−108 to 290)	0.15

**Table 3:** Cost-consequence balance sheet at 6-month follow-up

Costs and consequences	iSupport	Control
Total delivery costs (e-coach time)	Total cost: £1845Mean cost per participant: £10.54	Total cost: £337Mean cost per participant: £1.90
Carer		
Public sector resource use costs	Total costs: £23 385Mean cost per participant: £292	Total costs: £41 371Mean cost per participant: £371
Wider societal resource use and informal care costs	Total: £83 690Mean cost per participant: £771	Total costs: £98 242Mean cost per participant: £785
People living with dementia		
Public sector resource use costs	Total costs: £108 994Mean cost per participant: £893	Total costs: £112 902Mean cost per participant: £921
Wider societal resource use costs	Total costs: £134 053Mean cost per participant: £1099	Total costs: £140 273Mean cost per participant: £1088
Consequence		
Carer health-related quality of life (EQ-5D-5L)	Mean EQ-5D-5L index score: 0.84	Mean EQ-5D-5L index score: 0.86

For the wider societal perspective, we used the proxy good method for calculating informal care costs for our base case analysis within our wider societal perspective. The proxy good method (also known as the replacement cost method) regards work from the perspective of society and requires multiplying the number of hours spent completing informal care tasks by a shadow price market substitute. The informal care shadow price assigned in this analysis was the gross wage rate of a home care worker at £15.98 (assuming a salary of £20 284 per year, salary on-costs of £5151 per year and 1591 working hours per annum). These costs were derived from the Personal Social Services Research Unit (PSSRU) for cost year 2021/22.^23^

At baseline, 3 month and 6 month follow-up, participants were asked to report the number of hours spent during the last week on household activities, personal care and practical support relating to their informal caring role. The estimated hourly cost of a professional home care worker (£15.98) was multiplied by total hours reported for completing these tasks. In our base case assumptions, we included all hours reported by carer participants. For participants who reported very high numbers of hours for informal care tasks, we conducted sensitivity analysis to vary the number of hours reported. Where participants reported over 84 hours per week (12 hours per day over a 7-day period), we used the average number of total hours for informal care tasks reported across participants for each timepoint. We also conducted a separate sensitivity analysis using the opportunity cost method to explore variations in our costing assumptions to calculate informal care costs (see online supplemental file 4). From the public sector perspective, subgroup analysis was conducted to explore the differences in mean total resource use costs across subgroup categories of total time spent on the iSupport platform.

#### Health-related quality of life

A complete case analysis of EQ-5D-5L data at baseline and 6 months was undertaken. Mean EQ-5D-5L scores at baseline and 6 months were reported with 95% CIs estimated using 5000 nonparametric bootstrap sampling (non-parametric bootstrap bias corrected and accelerated). Due to negatively skewed data, the non-parametric independent Mann-Whitney U test was used to compare mean score differences between the control and intervention groups at a p value significance level of 0.05. We compared the mean EQ-5D-5L scores of the intervention and control group with published UK population norms for age-matched (to sample mean age) individuals aged 55–64 years.^25^

#### Sensitivity analysis

Three sensitivity analyses were conducted. First, outliers were removed using the IQR approach to explore the impact on resource use costs. The outlier cut point was set as 1.5 times the IQR. Cost values that were higher than the third quartile plus 1.5*IQR were removed. The second sensitivity analysis explored outliers in informal care hours (defined as ≥84 hours per week) reported by carers. The outliers then were replaced with the average number of informal care hours reported across both groups at each time point. In addition, a separate sensitivity analysis was conducted to explore informal care costs using the opportunity cost method.

## Results

### Participant characteristics

The iSupport trial comprised 352 participants: 175 randomised to the iSupport intervention group and 177 to the usual care control group (table 1). The mean age of participants in the intervention and control group was 63 and 62, respectively. In terms of the relationship between carer and person living with dementia, the majority of carers were children of the person living with dementia (50% in the intervention group; 51% in the control group) or were the spouse/partner of the person living with dementia (44% in the intervention group; 42% in the usual care group). The majority of carers in both the intervention (94%) and control (93%) groups identified as white English/Welsh/Scottish/Northern Irish/British (table 1). The mean number of years providing informal care was slightly higher in the intervention group (4.3) compared with the control group (3.9) (table 1). Further details on participant characteristics are presented in table 1.

### Delivery costs

The total cost of delivering iSupport was £1845 equating to a mean cost of £10.54 per participant (online supplemental file 4, Table 1), comprising the cost of e-coach time completing technical support and further associated delivery tasks. These costs accounted for salary including on-costs and a 30% uplift for overheads. The total costs for usual care in the control group were £337, equating to a mean cost of £1.90 per participant (online supplemental file 4, Table 1). This comprises £220 for e-coach tasks and £117 for postage costs to deliver booklets to control group participants who requested a physical copy. All control group participants received an electronic copy of the booklet. Both electronic and physical copies were provided by Alzheimer’s Society free of charge.

### Health-related quality of life

**Figure 2:**
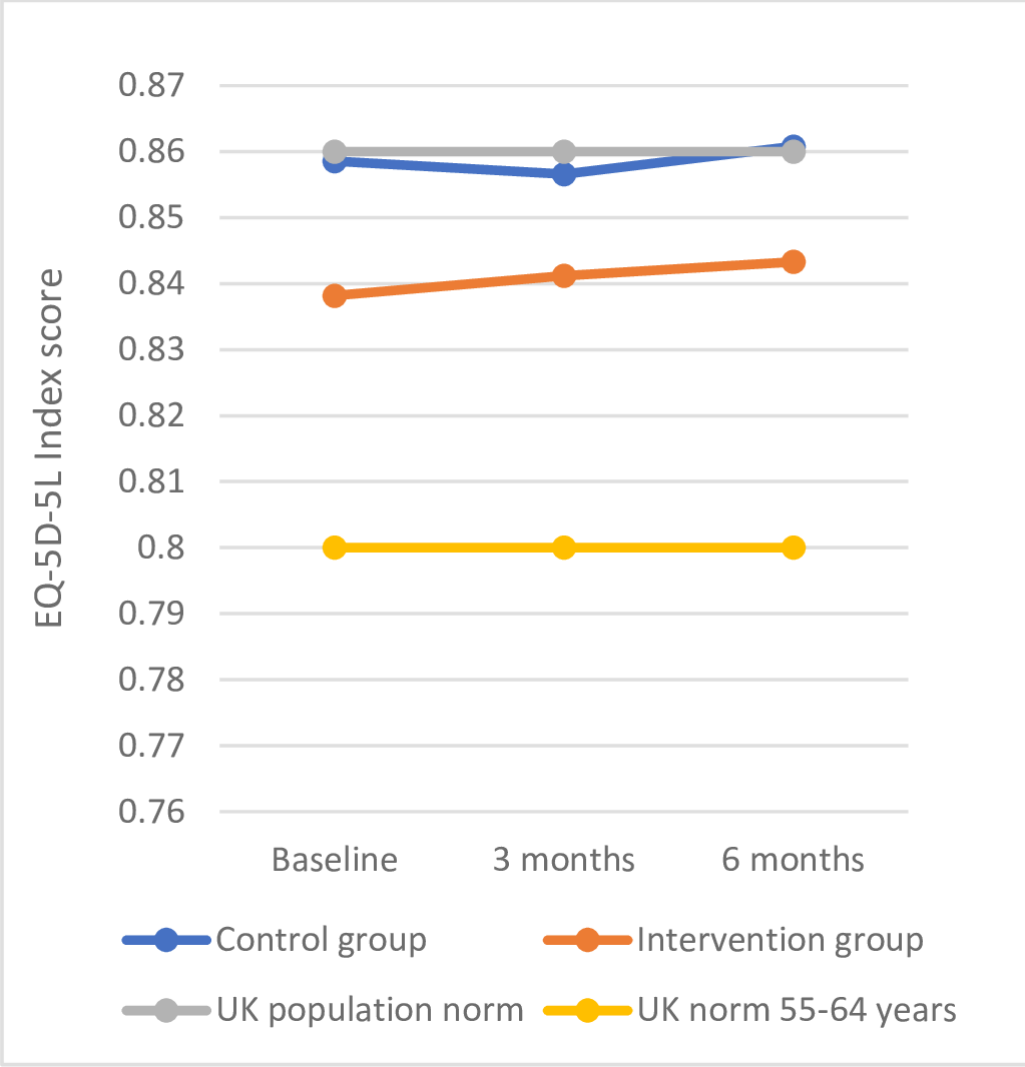
Comparing the EQ-5D-5L index score of iSupport dementia carers with UK population norm.

The number of EQ-5D-5L control (n=136) and intervention (n=125) complete cases at baseline and 6 months totalled 261. The mean change difference at 6 months between intervention and control groups was −0.003 (95% CI: −0.02 to 0.03), with no significant between-group differences at 6 months (p=0.67). At baseline, mean EQ-5D-5L scores for both intervention (0.84) and control (0.86) groups were similar to the age-matched UK population norm (0.80).^25^ At 6 months, there was no change in mean scores (intervention=0.84; control=0.86) (figure 2). The baseline characteristics of EQ-5D-5L complete cases were similar between intervention and control groups. There were some small discrepancies between the complete case EQ-5D-5L sample and main sample for age, gender, paid work status, travel distance and experience providing informal care (online supplemental file 4, Table 4).

### Resource use costs from the public sector perspective

#### Carers

Total resource use costs from the base case analysis conducted from a public sector perspective were £48 632 and £29 516 at baseline for the intervention and control groups, respectively (online supplemental file 4, table 15). The main cost drivers for the high resource use costs at baseline among the intervention group were the high frequencies of GP visits, support/educational group contacts and inpatient hospital stays reported by carer participants (online supplemental file 4, Table 5). Compared with the control group, the intervention group had higher mean costs for GP (£27 vs £39) and nurse (£15 and £26) consultations and support/education group (£8 and £17) at baseline. Total resource use costs for the intervention group decreased by 27.9% (−£13 553) between baseline and 3-month follow-up and decreased by 33.3% (−£11 694) between 3-month and 6-month follow-up (online supplemental file 4, Table 15). For the control group, total resource use costs increased by 21.1% (£6220) between baseline and 3-month follow-up and increased by 15.8% (£5634) between 3-month and 6-month follow-ups (online supplemental file 4, Table 15). Total resource use costs between baseline and 6-month follow-up decreased by £25 247 in the intervention group and increased by £11 856 in the control group (online supplemental file 4, Table 15).

From the public sector perspective, the mean resource use cost per participant at baseline was £278 (95% CI: 168 to 441) in the intervention group and £168 (95% CI: 124 to 217) in the control group (table 2). When compared with baseline, results indicated a significant difference (p=0.003) in the mean change in costs of community-based resource use between groups after 6 months. For the intervention group, the mean cost of community services after 6 months was less than the control group (table 2). There was no significant difference (p=0.57) in the mean change in hospital service use costs between groups between baseline and 6-month follow-up (table 2). Between baseline and 6 months, there was a significant difference (p=0.003) in the mean change in public sector (community and hospital-based) resource use costs between groups. For the intervention group, the mean cost of resource use after 6 months was less than the control group (table 2). The cost-consequence balance sheet can be viewed in table 3. Sensitivity analysis reported that when outliers were removed, no significant difference in the mean change in public sector resource use costs between groups (online supplemental file 4, Table 13).

From the public sector perspective, subgroup analysis was conducted to explore differences in mean total resource use costs across subgroup categories of total time spent on the iSupport platform (online supplemental file 4, Table 6). 32 participants (18%) spent zero time on the iSupport platform and 20 participants (11%) spent 3 hours or more. The increase in total mean resource use costs between baseline and 6-month follow-up for those who spent zero time on the platform was £395, whereas the increase between baseline and 6-month follow-up for carers accessing the platform for 3 hours or more was £4 (online supplemental file 4, Table 6).

#### People living with dementia

For people living with dementia, total resource use costs from the public sector perspective were £127 115 and £131 681 at baseline for the intervention and control groups, respectively (online supplemental file 4, Table 7). Total resource use costs for the intervention group decreased by 30.7% (−£39 043) between baseline and 3-month follow-up and then increased by 23.8% (£20 922) between 3-month and 6-month follow-ups. For the control group, total resource use decreased by 14.3% (−£18 779) between baseline and 3 months and then increased by 6% (£6814) between 3-month and 6-month follow-ups (online supplemental file 4, Table 7). Total resource use costs between baseline and 6-month follow-up decreased by £18 122 in the intervention group and decreased by £11 964 in the control group (online supplemental file 4, Table 7).

From a public sector perspective, the mean total resource use cost per participant at baseline was £726 (95% CI: 582 to 899) in the intervention group and £744 (95% CI: 569 to 945) for the control group (online supplemental file 4, Table 8). At 6 months, the mean total resource use cost per participant increased to £893 (95% CI: 627 to 1186) for the intervention group and increased to £921 (95% CI: 646 to 1234) for the control group (online supplemental file 4, Table 8). Between baseline and 6 months, there was no significant difference in the mean change in total resource use costs between groups (p=0.48) (online supplemental file 4, Table 8).

### Wider societal perspective (including informal care costs for carers)

#### Carers

The secondary analysis adopted a wider societal perspective to consider the costs of private and third sector service use costs and informal care costs (using the proxy good method), in addition to the costs that befall on the public sector. Unit costs can be viewed in online supplemental file 4, Table 3. Our base case analysis of informal care costs included any outliers of very high hours of informal care (defined as reporting 12 hours or more per day of informal care or 84 hours per week). Total and mean (SD) informal care time reported by carers at each time point can be viewed in online supplemental file 4, Table 9.

For the intervention group, total resource use costs from the wider societal perspective including public sector, private, third sector and informal care costs at baseline was £134 728 (online supplemental file 4, Table 15). Wider societal perspective total resource use and informal care costs decreased to £88 452 at 3-month follow-up and then decreased again to £83 691 at 6 months (online supplemental file 4, Table 15). For the control group, wider societal perspective total resource use and informal care costs at baseline were £109 222, decreased to £94 949 at 3-month follow-up and then increased to £98 242 at 6 months (online supplemental file 4, Table 15). From baseline to 6-month follow-up, total resource use and informal care costs from the wider societal perspective decreased by £51 038 in the intervention group and by £10 980 in the control group (online supplemental file 4, Table 15). There was no significant difference between groups (p=0.23) in the mean change in total resource use and informal care costs from the wider societal perspective between baseline and 6 months (table 2). Sensitivity analysis reported that when outliers were removed, there was a significant difference in the mean change in community service use costs between groups (p=0.018) (online supplemental file 4, Tables 13 and 14).

There was a significant difference between groups (p=0.041) in the mean change in costs of informal care between baseline and 6 months, with a reduction in informal care costs observed in the control group (table 2). Sensitivity analysis was conducted to replace outliers in informal care hours (defined as ≥84 hours per week reported by carers) with the average number of informal care hours reported across both groups at each time point to explore variation in costs. A significant difference (p=0.048) in the mean change in informal care costs between groups was found, with a decrease in mean informal care costs between baseline and 6 months for the control group (table 2).

A separate sensitivity analysis was conducted to explore informal care costs using the opportunity cost method. From baseline to 6-month follow-up, total resource use costs from the wider societal perspective decreased by £50 430 in the intervention group and by £12 308 in the control group (online supplemental file 4, Table 10). There was no significant difference between groups (p=0.23) in the mean change in total resource use and informal care costs from the wider societal perspective between baseline and 6 months when using the opportunity cost method to value informal care (online supplemental file 4, Table 11).

#### People living with dementia

For people living with dementia, total resource use costs from the wider societal perspective were £151 059 and £146 461 at baseline for intervention and control groups, respectively (online supplemental file 4, Table 7). Total resource use costs from the wider societal perspective for the intervention group decreased by 21.8% (−£32 951) between baseline and 3-month follow-up and then increased by 13.5% (£15 945) between 3-month and 6-month follow-ups. For the control group, total resource use from the wider societal perspective decreased by 13.8% (−£20 200) between baseline and 3 months and then increased by 11.1% (£14 012) between 3-month and 6-month follow-ups (online supplemental file 4, Table 7).

From the wider societal perspective, the total mean resource use cost per participant at baseline was £876 (95% CI: 716 to 1063) in the intervention group and £836 (95% CI: 647 to 1047) for the control group (online supplemental file 4, Table 12). At 6 months, the total mean resource use cost per participant increased to £1088 (95% CI: 807 to 1407) for the intervention group and increased to £1099 (95% CI: 802 to 1420) for the control group. There was no significant difference in the mean change in total resource use costs between groups between baseline and 6-month follow-up (p=0.37) (online supplemental file 4, Table 12).

For 95% CI using non-parametric bootstrapping, 5000 samples are bias-corrected and the accelerated method is used. Due to non-normality of cost data, Mann-Whitney U independent t test was conducted with p value significance level at 0.05. The mean cost of resource use from the societal perspective included privately funded and third-sector resource use, in addition to public sector resource use.

## Discussion

To our knowledge, this is the first CCA of a self-guided e-health intervention to support carers of people living with dementia. There was a significant difference in the mean change in public sector (community and hospital-based) resource use costs between groups after 6 months. At baseline, public sector resource use costs were higher in the intervention group compared with the control group. The main cost drivers for the high resource use costs at baseline among the intervention group participants were the self-reported high number of GP visits, support/educational group contacts and inpatient hospital stays. Results from the subgroup analysis found that participants who spent zero time on the iSupport platform had higher total resource use costs after 6 months. However, the sample size of between the subgroup categories was small. From the wider societal perspective, there was no significant difference in mean total resource use and informal care costs for carers at 6 months. For people living with dementia, there was no significant difference in mean public sector resource use costs between groups at 6 months or in mean resource use and informal care costs between groups at 6 months.

For carers between baseline and 6 months, there was a significant difference (p=0.003) in the mean change in public sector (community and hospital-based) resource use costs between groups. For the intervention group, the mean cost of resource use after 6 months was less than the control group. Despite reductions in public sector costs, the intervention reported no significant impact on resource use costs from the wider societal perspective. Due to this finding, coupled with the issues around the accessibility of online platforms for older carers, the intervention may not be justified from a societal viewpoint. However, it must be noted that although a reduction in health and social care resource use may result in reduced costs, we must not work under the assumption that using less resources is a favourable outcome. It may not represent positive findings as people from different socioeconomic backgrounds with varying circumstances will consume resources differently. It is acknowledged that low-income, poor-quality housing and low mental well-being are linked to poorer health outcomes, which often leads to a high consumption of health and social care resources. Nevertheless, higher income is often associated with better health outcomes potentially linked to lower overall health and social care resource use. Higher socioeconomic status may result in increased use of some services as greater financial independence enables individuals to attend regular healthcare appointments and preventative screening services, reducing the need for less costly treatment further down the line. Individuals with an educational advantage and higher levels of health literacy may know how best to navigate healthcare systems and make more informed decisions regarding care. There may also be individuals in the middle of the socioeconomic hierarchy who may not receive much health and social care support. Consequently, what may be found is an inverted U-shaped relationship between health and social care resource use and level of income/education, and future research should explore this. By accessing the iSupport platform, carers may also feel better supported to link into services which may explain the counterfactual outcomes reported in this study.

Participants in both intervention and control groups reported similar levels of health-related quality of life (measured using the EQ-5D-5L) to UK population norms.^25^ High EQ-5D-5L scores captured in this study may represent individuals who already have strong support systems and effective coping mechanisms. Moreover, the mean years of caring experience in both the intervention and control groups were high (4.3 and 3.9 for the intervention and control groups, respectively), which may further explain the high EQ-5D-5L scores reported in this study. The iSupport RCT focused on specific outcome measures which may not be the most impacted areas for dementia carers; however, future research on the most appropriate outcome measures in this population is needed. High EQ-5D-5L scores may also be explained by under-reporting difficulties when completing the measure with researchers, as they may feel stigmatised for revealing their true struggles, potentially leading to biased results. Future research may explore different outcome measures in this population, such as measures that capture any improvements in self-regulation or coping strategies following participation in e-health interventions. An important limitation of this study is the sample’s homogeneity which was a predominantly white and highly educated demographic. In consequence, this study may not reflect the experiences of participants with greater needs or accurately reflect patterns of resource use costs across diverse populations. This may have significant impacts on the generalisability of the study findings as participants with the greatest needs for support may have been excluded.

Self-guided e-health interventions offer flexible support that can be upscaled across large geographical areas. Our findings indicate that iSupport is a low-cost intervention in terms of delivery due to the absence of a facilitator delivering the programme; however, this is likely to impact the intervention effectiveness. Given null findings on the clinical effectiveness of iSupport and considering the self-guided nature and low participant engagement with the platform, promoting this intervention in its current format may not be worthwhile, particularly in contexts where alternative support services and information are accessible. Self-guided interventions rely on carers’ capacity to learn by themselves, requiring a high level of motivation and engagement among learners. Moreover, facilitator-led content may assist in learning capacity among participants in this study with differing levels of digital literacy. Future implementation of e-health programmes for carers may benefit from in-person support to deliver information to carers, enabling more tailored advice for participants and enhancing the effectiveness of the intervention. Collecting information on e-health interventions for dementia carers that offer more tailored advice and support would also provide invaluable insights on individual needs and how interventions could be refined to optimise resource allocation decisions. This may lead to better support for carers and people living with dementia and could highlight how e-health interventions could complement or replace traditional services.

This analysis considered the health and social care resource use from a public sector, as well as the impact on wider societal costs such as third sector and privately funded resource use, in addition to unpaid informal care costs. This study provided detailed costings of resource use utilised by study participants. We collected information on the reason participants attended secondary care, allowing us to provide detailed cost information on their resource utilisation. We also collected information on hours spent by carers performing informal care tasks, and the findings of this study indicate that informal care provided by family and friends of people living with dementia accounts for a significant contribution to societal costs in the UK. However, accurately measuring the full costs of informal care to acknowledge its value to society presents particular challenges. This study relied on participants’ estimation of hours spent on caregiving tasks which can often be difficult to accurately recall, especially for carers who may be under considerable stress due to their caregiver role. Moreover, informal care often comprises fragmented tasks which are entwined into daily living, and the time taken to complete different tasks will vary across carers, further adding to the complexity of accurately measuring informal care costs. A further limitation of this study was that we did not calculate work productivity factors such as absenteeism or presenteeism within our wider societal perspective of our analysis. Future research should consider potential costs and outcomes from the employer perspective. Although people who spent more time on iSupport demonstrated less change in total health resource use costs, the results of subgroup analysis cannot be generalised due to the small subgroup sample size. This study assessed costs at 3 and 6 months; therefore, it does not provide insights into the sustained impact of iSupport on health and social care resource use or informal care costs. The initial impacts reported, such as reductions in resource use, may diminish or plateau over longer time horizons as participants require ongoing support beyond the scope of the intervention.

This study offers the first available cost analysis of iSupport. The costs and consequences of self-guided e-health interventions for dementia carers have not been established previously in the literature, limiting our ability to compare our findings with published evidence. It is fundamental to acknowledge that any future comparisons between our study findings and international studies of iSupport will be difficult due to variations in measurement and valuation of costs, utilisation of distinct resources and methods of valuing informal care across studies conducted in different countries.

## Conclusion

This study shows that self-guided e-health interventions for dementia carers may have the potential to reduce health and social care resource use in the public sector, but evidence relating to their effectiveness and cost-effectiveness is lacking. We must not work on the assumption that using fewer resources is a favourable outcome, as there may be individuals who have not been linked into appropriate support. By accessing iSupport, individuals might feel better supported to link into services, which may explain the counterfactual outcomes reported in this analysis.

## Supplementary material

10.1136/bmjopen-2024-095611 online supplemental file 1

10.1136/bmjopen-2024-095611 online supplemental file 2

10.1136/bmjopen-2024-095611 online supplemental file 3

10.1136/bmjopen-2024-095611 online supplemental file 4

## Data Availability

Data are available upon reasonable request.
