# Supplementary material for: Cost-consequence analysis of an e-health intervention to reduce distress in dementia carers: results from the iSupport randomised controlled trial
Source: BMJ Open. 2025 May 16;15(5):e095611. doi: 10.1136/bmjopen-2024-095611 (PMC12086930; doi:10.1136/bmjopen-2024-095611)
Supplement: online supplemental file 1 [file bmjopen-15-5-s001.docx]

**CHEERS 2022 Checklist (Husereau et al., 2022).**

|  | **Item** | **Guidance for Reporting** | **Reported in section** |
| --- | --- | --- | --- |
| **TITLE** | | |  |
| Title | 1 | Identify the study as an economic evaluation and specify the interventions being compared. | Title, p.3 |
| **ABSTRACT** | | |  |
| Abstract | 2 | Provide a structured summary that highlights context, key methods, results and alternative analyses. | Abstract, p.3 |
| **INTRODUCTION** | | |  |
| Background and objectives | 3 | Give the context for the study, the study question and its practical relevance for decision making in policy or practice. | Introduction, p.5 |
| **METHODS** | | |  |
| Health economic  analysis plan | 4 | Indicate whether a health economic analysis plan was developed and  where available. | Methods, p.7 |
| Study population | 5 | Describe characteristics of the study population (such as age range, demographics, socioeconomic, or clinical characteristics). | Methods, p.5-6 and Results, p.8-11 |
| Setting and location | 6 | Provide relevant contextual information that may influence findings. | Methods, p.5 |
| Comparators | 7 | Describe the interventions or strategies being compared and why chosen. | Methods, p.6 |
| Perspective | 8 | State the perspective(s) adopted by the study and why chosen. | Methods, p.7 |
| Time horizon | 9 | State the time horizon for the study and why appropriate. | Methods, p.7-8 |
| Discount rate | 10 | Report the discount rate(s) and reason chosen. | Methods, p.7 |
| Selection of outcomes | 11 | Describe what outcomes were used as the measure(s) of benefit(s) and harm(s). | Methods, p.7-8 |
| Measurement of outcomes | 12 | Describe how outcomes used to capture benefit(s) and harm(s) were measured. | Methods, p.7-8 |
| Valuation of outcomes | 13 | Describe the population and methods used to measure and value outcomes. | Methods, p.7-8 |
| Measurement and valuation of resources  and costs | 14 | Describe how costs were valued. | Methods, p.7-8 |
| Currency, price date, and conversion | 15 | Report the dates of the estimated resource quantities and unit costs, plus the currency and year of conversion. | Methods, p.7-8 |
| Rationale and  description of model | 16 | If modelling is used, describe in detail and why used. Report if the model is publicly available and where it can be accessed. | Not applicable |
| Analytics and assumptions | 17 | Describe any methods for analysing or statistically transforming data, any extrapolation methods, and approaches for validating any model used. | Methods, p.7-8 |
| Characterizing heterogeneity | 18 | Describe any methods used for estimating how the results of the study vary for sub-groups. | Methods, p.7-8 |
| Characterizing  distributional effects | 19 | Describe how impacts are distributed across different individuals  or adjustments made to reflect priority populations. | Methods, p.7-8 |
| Characterizing uncertainty | 20 | Describe methods to characterize any sources of uncertainty in the analysis. | Methods, p.7-8 |
| Approach to engagement with patients and others affected by the study | 21 | Describe any approaches to engage patients or service recipients, the general public, communities, or stakeholders (e.g., clinicians or payers) in the design of the study. | Methods, p.6 |
| **RESULTS** | | |  |
| Study parameters | 22 | Report all analytic inputs (e.g., values, ranges, references) including uncertainty or distributional assumptions. | Results, p.8-15 |
| Summary of main results | 23 | Report the mean values for the main categories of costs and outcomes of interest and summarise them in the most appropriate overall measure. | Results, Table 2, p.14 |
| Effect of uncertainty | 24 | Describe how uncertainty about analytic judgments, inputs, or projections affect findings. Report the effect of choice of discount rate and time horizon, if applicable. | Results, p.8-15 |
| Effect of engagement with patients and others affected by the study | 25 | Report on any difference patient/service recipient, general public, community, or stakeholder involvement made to the approach or findings of the study | Methods, p.6 |
| **DISCUSSION** | | |  |
| Study findings, limitations, generalizability, and current knowledge | 26 | Report key findings, limitations, ethical or equity considerations not captured, and how these could impact patients, policy, or practice. | Discussion, p.16-18 |
| **OTHER RELEVANT INFORMATION** | | | |
| Source of funding | 27 | Describe how the study was funded and any role of the funder in the identification, design, conduct, and reporting of the analysis | Page 2 |
| Conflicts of interest | 28 | Report authors conflicts of interest according to journal or  International Committee of Medical Journal Editors requirements. | Page 2 |

Husereau D, Drummond M, Augustovski F, de Bekker-Grob E, Briggs AH, Carswell C, Caulley L, Chaiyakunapruk N, Greenberg D, Loder E, Mauskopf J, Mullins CD, Petrou S, Pwu RF, Staniszewska S; CHEERS 2022 ISPOR Good Research Practices Task Force. Consolidated Health Economic Evaluation Reporting Standards 2022 (CHEERS 2022) Statement: Updated Reporting Guidance for Health Economic Evaluations. BMJ. 2022;376:e067975.

The checklist is Open Access distributed in accordance with the terms of the Creative Commons Attribution (CC BY 4.0) license, which permits others to distribute, remix, adapt and build upon this work, for commercial use, provided the original work is properly cited. See: [http://creativecommons.org/licenses/by/4.0/.](http://creativecommons.org/licenses/by/4.0/)

**STUDY TITLE:** A randomised controlled trial and feasibility study of the effects of an e-health intervention ‘iSupport’ for reducing distress of dementia carers, especially in the ongoing pandemic of COVID-19 - **Protocol version 5 dated 06/09/2023**

**Contents**

[Contents 3](#_Toc144987456)

[Table of Figures 6](#_Toc144987457)

[Note on the structure of this protocol 7](#_Toc144987458)

[1. Background and rationale 7](#_Toc144987459)

[1.1 What is the problem being addressed? 7](#_Toc144987460)

[1.2 Why is this research important? 7](#_Toc144987461)

[1.3 Brief review of published evidence 8](#_Toc144987462)

[2. Trial objectives and design 8](#_Toc144987463)

[2.1 Trial objectives and design 8](#_Toc144987464)

[2.2 Research questions 9](#_Toc144987465)

[2.3 Trial expected duration 10](#_Toc144987466)

[2.4 Trial flowchart 10](#_Toc144987467)

[3. Selection and withdrawal of trial participants 11](#_Toc144987468)

[3.1 Inclusion criteria 11](#_Toc144987469)

[3.2 Exclusion criteria 11](#_Toc144987470)

[3.3 Trial consent procedure 12](#_Toc144987471)

[3.4 Randomisation procedure 13](#_Toc144987472)

[3.5 Unblinding procedure 14](#_Toc144987473)

[3.6 Withdrawal of participants 14](#_Toc144987474)

[4. Trial procedures 14](#_Toc144987475)

[4.1 Planned intervention 14](#_Toc144987476)

[4.2 Comparison group 15](#_Toc144987477)

[4.3 Setting and context 16](#_Toc144987478)

[4.3.1 Research sites 16](#_Toc144987479)

[4.3.2 Organogram of research sites and study reporting 17](#_Toc144987480)

[4.4 Sampling and sample size 17](#_Toc144987481)

[4.5 WS1 Randomised controlled trial 18](#_Toc144987482)

[4.5.1 Internal pilot study 19](#_Toc144987483)

[4.5.2 Selection of participants 19](#_Toc144987484)

[4.5.3 Primary outcome measures 20](#_Toc144987485)

[4.5.4 Secondary outcome measures 21](#_Toc144987486)

[4.5.5 Data collection 21](#_Toc144987487)

[4.5.6 Data analysis 22](#_Toc144987488)

[4.6 WS2 Process evaluation 22](#_Toc144987489)

[4.6.1 Quantitative data collection 23](#_Toc144987490)

[4.6.2 Qualitative data collection 23](#_Toc144987491)

[4.6.3 Data analysis 23](#_Toc144987492)

[4.7 WS3 health economics 24](#_Toc144987493)

[4.7.1 Outcome measures 24](#_Toc144987494)

[4.7.2 Data collection 25](#_Toc144987495)

[4.7.3 Data analysis 25](#_Toc144987496)

[5. WS4 Feasibility study objectives and design 26](#_Toc144987497)

[5.1 WS4 Feasibility study objectives and design 26](#_Toc144987498)

[5.2 WS4 Research questions 26](#_Toc144987499)

[5.3 WS4 Feasibility study expected duration 26](#_Toc144987500)

[5.4 WS4 Feasibility study flowchart 27](#_Toc144987501)

[6. Selection and withdrawal of WS4 feasibility study participants 27](#_Toc144987502)

[6.1 WS4 Inclusion criteria 27](#_Toc144987503)

[6.2 WS4 Exclusion criteria 28](#_Toc144987504)

[6.3 WS4 Feasibility study consent procedure 28](#_Toc144987505)

[6.4 WS4 Randomisation and unblinding 28](#_Toc144987506)

[6.5 WS4 Withdrawal of participants 28](#_Toc144987507)

[7. WS4 Feasibility study procedures 28](#_Toc144987508)

[7.1 WS4 Planned intervention 28](#_Toc144987509)

[7.2 WS4 Setting and context 28](#_Toc144987510)

[7.3 WS4 Feasibility study 28](#_Toc144987511)

[7.3.1 Phase 1: Adaptation of ‘iSupport’ for younger dementia carers 28](#_Toc144987512)

[7.3.2 Phase 2: Feasibility testing ‘iSupport’ for younger dementia carers 29](#_Toc144987513)

[7.3.3 Sampling and sample size 29](#_Toc144987514)

[7.3.4 Selection of participants 30](#_Toc144987515)

[7.3.5 Data collection 30](#_Toc144987516)

[7.3.6 Data analysis 30](#_Toc144987517)

[8. Translating ‘iSupport’ into Welsh 31](#_Toc144987518)

[9. WS5 Feasibility testing the Bengali adaptation of iSupport 32](#_Toc144987519)

[9.1 WS5 Feasibility study objectives and design 32](#_Toc144987520)

[9.2 WS5 Research questions 32](#_Toc144987521)

[9.3 WS5 Feasibility study expected duration 32](#_Toc144987522)

[9.3.1 WS5 Feasibility study Flowchart 33](#_Toc144987523)

[9.4 Selection and withdrawal of WS5 feasibility study participants 33](#_Toc144987524)

[9.5 WS5 Inclusion criteria 34](#_Toc144987525)

[9.6 WS5 Exclusion criteria 34](#_Toc144987526)

[9.7 WS5 Feasibility study consent procedure 34](#_Toc144987527)

[9.8 WS5 Randomisation and unblinding 34](#_Toc144987528)

[9.9 WS5 Withdrawal of participants 34](#_Toc144987529)

[9.10 WS5 Feasibility study procedures 34](#_Toc144987530)

[9.11 WS5 Planned intervention 35](#_Toc144987531)

[9.12 WS5 Setting and context 35](#_Toc144987532)

[9.13 WS5 Sampling and sample size 35](#_Toc144987533)

[9.14 WS5 Data collection 35](#_Toc144987534)

[9.15 WS5 Data analysis 36](#_Toc144987535)

[10. WS6 ‘CareFit’ for dementia carers 36](#_Toc144987536)

[10.1 WS6 research objectives and design 36](#_Toc144987537)

[10.2 WS6 Research definitions 37](#_Toc144987538)

[10.3 WS6 Planned intervention 37](#_Toc144987539)

[10.3.1 Overview of the ‘CareFit’ app “tabs” with a description of their function 38](#_Toc144987540)

[10.4 WS6 research expected duration 39](#_Toc144987541)

[10.5 Selection and withdrawal of WS6 research participants 39](#_Toc144987542)

[10.6 WS6 Inclusion criteria 39](#_Toc144987543)

[10.7 WS6 Exclusion criteria 40](#_Toc144987544)

[10.8 WS6 research consent procedure 40](#_Toc144987545)

[10.9 WS6 Randomisation and unblinding 41](#_Toc144987546)

[10.10 WS6 Withdrawal of participants 41](#_Toc144987547)

[10.11 WS6 research procedures 41](#_Toc144987548)

[10.11.1 Professional staff who support carers of people with dementia 41](#_Toc144987549)

[10.11.2 Carers of people with dementia 41](#_Toc144987550)

[10.11.3 Stakeholder consultation to understand future use of the CareFit app 42](#_Toc144987551)

[10.12 WS6 Setting and context 43](#_Toc144987552)

[10.12.1 Safety considerations specific to WS6 43](#_Toc144987553)

[10.13 WS6 Sampling and sample size 43](#_Toc144987554)

[10.14 WS6 Data collection 43](#_Toc144987555)

[10.15 WS6 Data analysis 45](#_Toc144987556)

[11. Assessment of safety 45](#_Toc144987557)

[11.1 Definitions 45](#_Toc144987558)

[11.2 Collecting, recording and reporting of adverse events 45](#_Toc144987559)

[12. Project management 46](#_Toc144987560)

[12.1 Trial Steering Committee 47](#_Toc144987561)

[12.2 Independent Data Monitoring Committee 47](#_Toc144987562)

[12.3 Trial Management Group 47](#_Toc144987563)

[12.4 Patient and public involvement (PPI) 47](#_Toc144987564)

[12.5 Coronavirus (COVID-19) mitigation 48](#_Toc144987565)

[13. Ethics and regulatory approvals 48](#_Toc144987566)

[14. Monitoring 49](#_Toc144987567)

[14.1 Quality Assurance (QA) and Quality Control (QC) of data 49](#_Toc144987568)

[14.2 Risk assessment 49](#_Toc144987569)

[14.3 Monitoring plan 50](#_Toc144987570)

[14.4 Source data 50](#_Toc144987571)

[14.5 Direct access to source data and documents 50](#_Toc144987572)

[14.6 Confidentiality 50](#_Toc144987573)

[15. Data handling 50](#_Toc144987574)

[16. Pathways to impact 51](#_Toc144987575)

[17. Indemnity 51](#_Toc144987576)

[18. Financial aspects 52](#_Toc144987577)

[19. Definition of end of study 52](#_Toc144987578)

[20. Archiving 52](#_Toc144987579)

[21. Research expertise 52](#_Toc144987580)

[22. Research collaborators 53](#_Toc144987581)

[23. Protocol amendments 54](#_Toc144987582)

[23.1 Current version of the protocol 54](#_Toc144987583)

[23.2 Amendments 54](#_Toc144987584)

[24. Appendices 58](#_Toc144987585)

[25. References 60](#_Toc144987586)

**Table of Figures**

[Figure 1: Trial flowchart including sample sizes 10](#_Toc144987587)

[Figure 2: Consent procedure flowchart 12](#_Toc144987588)

[Figure 3: Overview of ‘iSupport’ intervention 15](#_Toc144987589)

[Figure 4: Organogram of research sites and study reporting 17](#_Toc144987590)

[Figure 5: Feasibility study flowchart including sample sizes 27](#_Toc144987591)

[Figure 6: WS5 study flowchart including sample sizes 33](#_Toc144987592)

**Note on the structure of this protocol**

For the purposes of this Protocol, the randomised controlled trial will be outlined first, and the feasibility study second. The randomised controlled trial will henceforth be referred to as the “trial”.

**1. Background and rationale**

**1.1 What is the problem being addressed?**

Estimates suggest 850,000 people in the UK live with dementia. Most (700,000) are cared for at home,^^[[1]](#endnote-1)^^ supported by a family member or friend who has little knowledge of the condition and how to best manage it. This is often described as ‘informal care’, in contrast to professional care provision. Informal carers (henceforth referred to as ‘carers’) are unpaid, often performing care tasks similar to those carried out by paid health or social service providers.^^[[2]](#endnote-2)^^ This raises two important points. First, there is a well-documented detrimental impact of caregiving on the physical and mental health of dementia carers.^^[[3]](#endnote-3)^^ Second, despite this detrimental impact to carers, informal care benefits their relatives and also society. For example, the total cost of dementia to society in the UK is £26.3 billion. £11.6 billion of this is contributed by the work of unpaid carers of people with dementia, higher than the £4.3 billion spent on healthcare costs and £10.3 billion spent on social care.^^[[4]](#endnote-4)^^ Given the financial contribution of informal caring on the one-hand, and the negative health impact on the other, Action area 5 of the global action plan on the public health response to dementia 2017-2025 prioritises supporting carers, calling for the provision of accessible evidence based information to improve knowledge and skills and prevent stress and health problems.^^[[5]](#endnote-5)^^ Although health is a devolved area of government policy, UK national dementia strategies^^[[6]](#endnote-6)^,^[[7]](#endnote-7)^,^[[8]](#endnote-8)^^ all make commitments to support the health and wellbeing of dementia carers. NICE ^^[[9]](#endnote-9)^^ recommend informal carers of people living with dementia should be offered training and psychoeducation to help them develop care skills and manage their own physical and mental health. Therefore access to appropriate, useful, low-cost, effective support for carers, with effective implementation strategies, is a priority for people living with dementia, their carers and service providers, and the focus of this research. This is especially important given the current pandemic, when many carers no longer have access to usual respite, leisure, and support services, finding themselves distanced and isolated.

**1.2 Why is this research important?**

**Sustained interest and intent:** The number of people with dementia in the UK is predicted to increase to 1,142,677 within 5 years and 2,092,945 by 2051, an increase of 40% and 156% respectively from the 2013 estimate.^4^ Currently there is no cure, with limited medical treatment options. Most people living with dementia are supported by informal carers. Sustaining the health and capabilities of these dementia carers is a public health priority.^5^

**Health need:** A meta-review concluded that being an informal carer for people with dementia is associated with psychological stress and physical ill-health.^3^ A meta-analysis comparing carers and non-carers found carers were more stressed, depressed, and had lower levels of subjective well-being, physical health, and self-efficacy than non-carers.^2^ The evidence generated from this research will examine how a low cost, accessible and scalable e-health intervention ‘iSupport’ may alleviate the detrimental human and economic impact of dementia. The WHO describes e-health as “the use of information and communication technologies (ICT) for health”.

**Expressed need:** A systematic review of dementia carers’ needs, as voiced themselves (i.e. not by a care professional) found that carers need: a) relevant information and knowledge; b) support with the management of care recipients’ functioning, behavioural and psychological symptoms; c) support with their own physical and mental health; d) support regarding their unbalanced social life.^^[[10]](#endnote-10)^^ The intervention to be tested ‘iSupport’ is specifically designed to address these needs of carers.

**Capacity to generate new knowledge:** This will be the first study in the UK and the first in a majority English-speaking population of a globally targeted e-health intervention for dementia carers. ‘iSupport’ is a recently developed evidence-informed online training and support programme for adult dementia carers to help them provide good care and take care of themselves. It was developed by the World Health Organisation in collaboration with Alzheimer’s Disease International and international experts, consequently ‘iSupport’ has the potential for significant global reach and impact.

**1.3 Brief review of published evidence**

Our ongoing systematic reviews found the most effective interventions for carers’ psychological health should incorporate both an educational component to enhance knowledge and a therapeutic component, such as CBT/cognitive reframing.^^[[11]](#endnote-11)^^ ‘iSupport’ incorporates both these components.

To date, there is no published evidence of the effectiveness of ‘iSupport’. In contrast to the Dutch, Portuguese and Indian evaluations of ‘iSupport’,^^[[12]](#endnote-12)^,^[[13]](#endnote-13)^,^[[14]](#endnote-14)^^ we will not screen participants and restrict our inclusion to those reporting clinically relevant levels of distress, depression or anxiety, we will instead use self-recognition of such outcomes from carers themselves. This ‘real world’ application will generate new knowledge about ‘iSupport’ as a public health approach to prevention.

The proposed feasibility study will also provide new knowledge about the impact of an adapted version of ‘iSupport’ on younger populations of carers. No published works of online interventions for young carers were identified in core databases.

**2. Trial objectives and design**

**2.1 Trial objectives and design**

The objectives of the trial can be separated into three work-streams (WS):

WS1. A definitive pragmatic individually randomised controlled trial across Wales, Scotland and England, with a six-month nested internal pilot. This will:

- Determine progression of the definitive trial based on a go/review/stop criteria (nested internal pilot).
- Determine the effectiveness of ‘iSupport’ in reducing symptoms of distress and/or depression.
- Determine the effectiveness of ‘iSupport’ in reducing symptoms of anxiety.
- Determine the effectiveness of ‘iSupport’ in improving dementia knowledge, relationship quality and resilience.
- Describe the trial sample according to demographic/socioeconomic characteristics.

WS2. A process evaluation will be conducted in line with the established guidelines for process evaluations of complex evaluations^^[[15]](#endnote-15)^,^[[16]](#endnote-16)^^ to determine the barriers and facilitators to the implementation of ‘iSupport’ at scale, and the extent it supports carers in the face of the ongoing or future COVID-19 pandemic. This will:

- Determine participant engagement and adherence to ‘iSupport’.
- Explore the mechanisms of change.
- Identify the external factors to ‘iSupport’ which influence the delivery and function of the intervention.
- Explore the contextual factors that influence the scalability of ‘iSupport’ into wider contexts using the CICI framework.^^[[17]](#endnote-17)^^

WS3. A parallel cost-effectiveness analysis, undertaken from both a public sector perspective (NHS, personal social services and local authorities), and a societal perspective (public sector plus opportunity costs). This will:

- Calculate the costs of implementing ‘iSupport’, including technical support and time spent supporting carers to use the tool.
- Explore patterns of, and estimate the cost of, health and social care resource use for carers in the ‘iSupport’ and comparison arms of the trial.
- Explore patterns of, and estimate the cost of, health and social care resource use for the care recipients of carers in the trial.
- Explore the opportunity cost of informal care through the measurement of informal care time, types of care task, impacts on carer’s leisure and employment hours, and carers’ willingness to pay for more support.
- Using QALYs derived from the EQ-5D-5L, determine the cost-effectiveness of ‘iSupport’ compared to the control condition; conduct secondary cost-effectiveness analyses using the Zarit Burden Interview^^[[18]](#endnote-18)^^ and the Centre for Epidemiological Studies of Depression Scale (CES-D10).^^[[19]](#endnote-19)^,^[[20]](#endnote-20)^^

**2.2 Research questions**

1. Is carer distress and/or symptoms of depression (primary outcomes) significantly reduced in participants allocated to receive ‘iSupport’ compared to participants allocated to a comparison group?

2. Are symptoms of anxiety (secondary outcome) significantly reduced, and resilience, relationship quality and dementia knowledge (secondary outcomes) significantly increased in participants allocated to receive ‘iSupport’ compared to participants allocated to a comparison group receiving standardised information about dementia?

3. What are participant and contextual barriers and facilitators to implementation of ‘iSupport’?

4. What potential mechanisms might underpin changes in outcomes from using ‘iSupport’?

5. What is the cost-effectiveness of ‘iSupport’ compared to standardised information about dementia?

6. What are the carers’ perspectives of ‘iSupport’ in relation to supporting them in an ongoing or future repeated pandemic such as COVID-19?

**2.3 Trial expected duration**

The total time scheduled for the trial has been extended in agreement with the funder to 42 months. Key milestones will be monitored as part of overall project management.

**2.4 Trial flowchart**

*Figure 1: Trial flowchart including sample sizes*

**3. Selection and withdrawal of trial participants**

Dementia carers (age 18+) in Wales, Scotland and England will be recruited through a range of approaches. Research Assistants will work with our Patient and Public Involvement (PPI) groups to promote the trial through social media to promote self-referral, and will use Join Dementia Research (JDR)^^[[21]](#endnote-21)^^ as a tool to identify potential participants. This is an online self-registration service that enables volunteers with memory problems or dementia, carers of those with memory problems or dementia and healthy volunteers to register their interest in taking part in research. Researchers can then contact volunteers, in line with the volunteers’ preferred method of contact, to further discuss potential inclusion.

Our study partners (Carers Trust and Alzheimer Scotland) and other non-statutory organisations will promote the study through their networks and to regional groups (including England), in order to reach a diverse range of dementia carers across different regions.

If recruitment from our collaborators is not happening to target, Research Assistants will approach memory clinics and dementia support groups to identify participants. This would first require NHS ethical approval. An IRAS form would be completed and approved prior to commencing recruitment from clinics. An amendment request would also be submitted to this ethics committee. Trusts that have capacity and sign an agreement with the Sponsor will be sent an Abridged Trial File. Participants attending memory clinics or other NHS secondary services will then be screened and consented by NHS researchers who have received ‘iSupport’ induction training, and following the procedures in this protocol.

All public facing documents, including a plain English/Cymraeg clir trial leaflet explaining the purpose of the research will be finalised in collaboration with our PPI group. We will establish a webpage explaining the study purpose and procedures. This will be hosted by the lead institution. Interested participants will be able to register through this website. All carers expressing interest in taking part will be provided with the leaflet and information sheet and have the opportunity to discuss the trial with the research assistants before committing.

**3.1 Inclusion criteria**

1) Adults (18+) who self-identify as an unpaid carer (partners, children, friends, etc.) of a person with dementia who is not living in a full-time care facility, caring at least weekly for at least 6 months.

2) Self-identify as experiencing at least some stress, depression or anxiety.

3) The care recipient has to have a confirmed diagnosis of dementia (through self-report of the carer, to reflect the ‘real world’ application of ‘iSupport’).

**3.2 Exclusion criteria**

1) Receiving psychological treatment from a mental health specialist at the time of recruitment.

2) Unable to comprehend written English.

3) No access to the internet.

4) Unable to give informed consent to the trial.

5) Have previously used ‘iSupport’ materials (in the last 12 months).

**3.3 Trial consent procedure**

We will use a remote method for assessing eligibility and taking consent, following a procedure successfully implemented in other studies by our co-investigators at UCL. Participants will electronically “sign” a statement of consent after having received the study information and consent forms via email, and having had a one-to-one phone or internet-based meeting with a researcher to ask questions. A flowchart of the consent procedure is below:


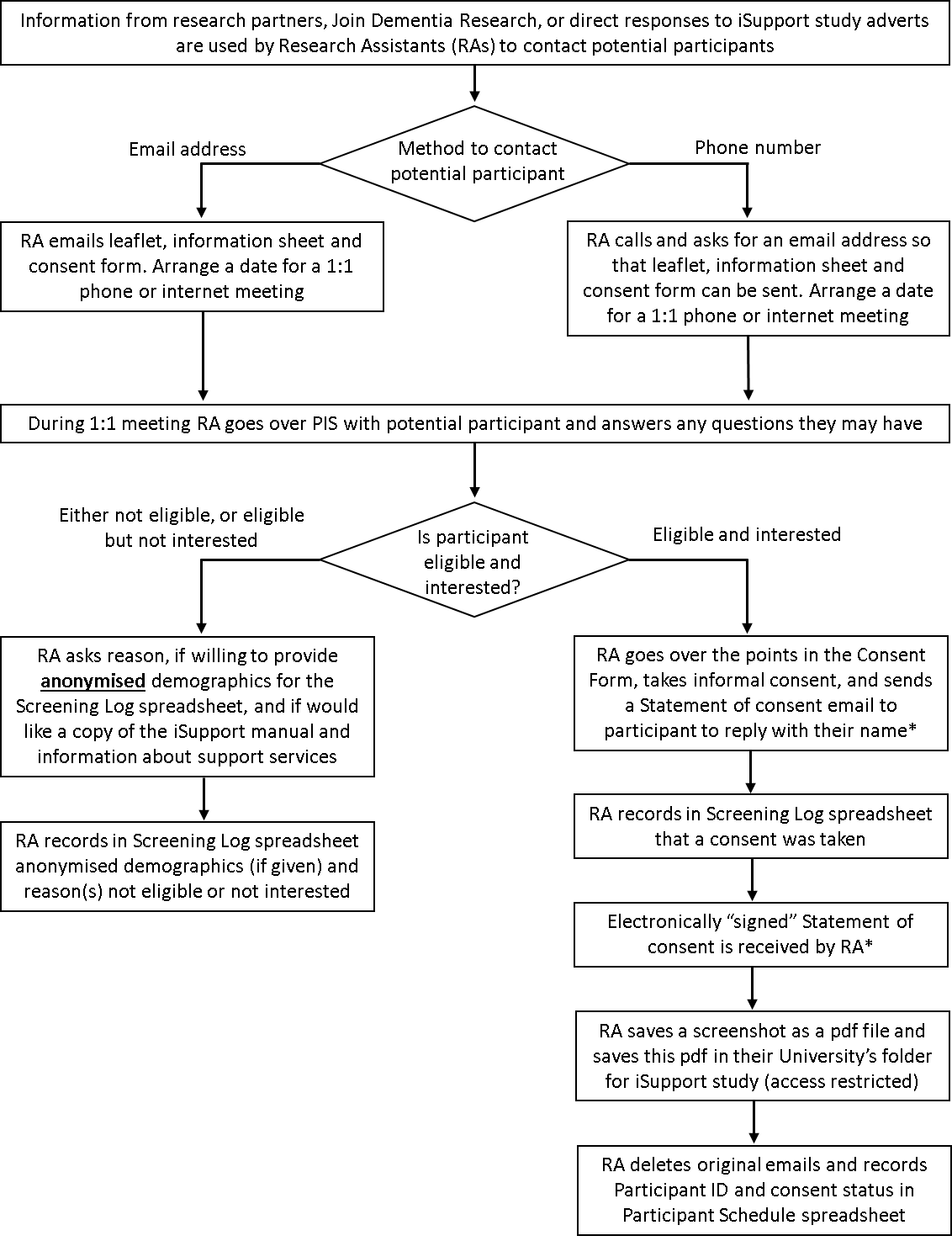


*Figure 2: Consent procedure flowchart*

The Statement of consent will read:

“I [NAME], have read the information sheet and consent forms for the study
titled ‘iSupport for Dementia Carers’. With this email, I hereby electronically
‘sign’ and consent to taking part in the study and to the [NUMBER] items

outlined on the consent form.”

*In the event participants do not use email or other messaging services (e.g. Whatsapp), paper versions of documents will be posted to their address and the procedure for taking consent would slightly differ: The participant would sign while on the phone with the researcher, post their signed consent form to the researcher to be copied and stored in a secure location (e.g. scanned to a secure computer folder and paper in a locked filing cabinet),, and a copy would then be returned to the participant.

In the unlikely event the participant cannot return either the statement of consent or a paper consent form, verbal consent would be sought. The research assistant would audio record the participant consenting to the trial and the recording would be securely stored as an audio file (e.g. MP3, WAV) in a secure computer folder.

**3.4 Randomisation procedure**

Randomisation will be performed by dynamic allocation to protect against subversion.^^[[22]](#endnote-22)^^ The algorithm will ensure that the trial maintains good balance to the allocation ratio of 1:1 both within each stratification variable and overall for the trial. Stratification variables will be site, along with age and gender, previously found to influence the outcome measure of caregiver distress.^^[[23]](#endnote-23)^^

Randomisation will be performed by the Research Assistant after completing the baseline assessment with the participant (see section 2.4 Trial flowchart). The Trial Manager and Chief Investigator will also be able to perform randomisations if required. The randomisation system will allow the user to check entry details before randomisation is performed. A simple confirmation email will be sent to the person who performed the randomisation. For research assistants these will not include any allocation information in order to keep them blinded.

The randomisation system will send a second unblinded email to the Trial Manager and Chief Investigator, informing them of all randomisations performed and the group allocations. They can then inform the participant of their allocation to either the intervention or comparison arm of the study. This will be done by emailing the details contained in the randomisation letter template (uploaded as part of research ethics submission). If the participant does not use email, they will be informed by phone and a letter will be sent to their address.

Randomisation will be achieved by secure web access to the remote randomisation centre at NWORTH, Bangor University. The randomisation system will be set up, maintained, and monitored independently of the trial statistician or other trial staff. A detailed randomisation specification will be drawn up prior to set up of the system that will detail the technical system requirements, this will be guided by NWORTH’s Standard Operating Procedures (SOPs).

**3.5 Unblinding procedure**

It is not possible to blind the individual participants in this trial, but the research assistants, health economists, co-investigators and trial statistician will remain blind until the blinded analysis detailed in the Statistical Analysis Plan has been conducted and reported to the trial team. The exception will be one of the co-investigators leading the process analysis. Unblinding will be performed following procedures outlined in NWORTH SOPs.

**3.6 Withdrawal of participants**

Participants are free to withdraw at any time during the trial without any impact on their future health and care. Participant data collected to the point of withdrawal will be used in the analysis set unless consent for this is specifically withdrawn.

**4. Trial procedures**

**4.1 Planned intervention**

‘iSupport’ is an internet-based psychoeducation and skills development intervention. The theoretical underpinnings of ‘iSupport’ are based on person-centred care, which recognises that dementia care should reflect the individual’s needs, personality and ability.^^[[24]](#endnote-24)^^ These elements are integrated into the interactive content of ‘iSupport’. The self-care techniques are based on theoretically informed programmes with some evidence for benefits, including psychoeducation, relaxation, behavioural activation, cognitive reframing, and problem-solving.^^[[25]](#endnote-25)^^

‘iSupport’ consists of five main themes and twenty-three accompanying exercises, namely: (i) introduction to dementia; (ii) being a carer; (iii) caring for me; (iv) providing everyday care; and (v) dealing with behaviour changes. Each exercise takes approximately 5-15 minutes and follows the same format: information about a topic presented; short interactive exercises and questions with instant feedback on responses; a summary of the lesson; a relaxation exercise.

‘iSupport’ is based on personal choice: carers can construct their own personalised plan and access which sessions they feel are most relevant to them at that point in time. It is anticipated the whole programme can be completed in 3 months. The programme can be followed via the internet using a personal computer or a tablet (e-health), or through a mobile phone accessing a ‘mobile friendly’ version of the platform (m-health).

To address potential inequity of uptake, a short video tutorial on how to use the programme will be developed and sent to all participants randomised to the intervention group. For the purpose of this research, participants will be advised to use ‘iSupport’ regularly in order to obtain the most benefit. They will be provided with the contact details of an ‘e-coach’, who will be trained to explain anything that is not clear about the ‘iSupport’ programme. This training will follow many of the good practice principles in this document:

<https://www.onlinecentresnetwork.org/sites/default/files/a6_your_guide_to_helping_older_people_use_the_internet.pdf> [accessed 10/03/2021]. The ‘e-coach’ will contact participants randomised to intervention shortly after randomisation, 1 month later and 2 months later (if required by the participant).

We will translate ‘iSupport’ into Welsh following WHO adaptation guidelines (see section 8. Translating ‘iSupport’ into Welsh). Approximately one-fifth of the Welsh population speak Welsh^^[[26]](#endnote-26)^^ and the Welsh Government is committed to offering bilingual services as part of health care provision.^^[[27]](#endnote-27)^^ A bilingual resource being widely available at no cost to the user will add value beyond the trial. To improve access, we will also develop audio/read aloud function for inclusion in the platform.

The figure below shows a visual overview of the intervention, and more information can be viewed in a short video produced by the WHO: <https://youtu.be/_g2KMgjukzs> [accessed 10/03/2021]

*Figure 3: Overview of ‘iSupport’ intervention*

**4.2 Comparison group**

Participants assigned to the comparison group will receive information about dementia developed by the Alzheimer’s Society.^^[[28]](#endnote-28)^^ This covers the topics of understanding the diagnosis, taking on the caring role, looking ahead, understanding and supporting the person with dementia, services, support and housing, finances, the later stages of dementia, end of life care and support, contact details of useful organisations. This information will be available online and/or in printed format. Carers can choose which format they prefer. Alongside this education, carers will receive care-as-usual. They can search for other information or seek help from other providers. Information about local context support and services will be obtained at baseline. Following the final data collection the participants allocated to the comparison group will be provided with access to ‘iSupport’.

**4.3 Setting and context**

The research will be undertaken with dementia carers who live in Wales, Scotland and England. Researchers will recruit and assess participants during a one-to-one interview over an internet-based service (e.g. Zoom, Teams or Skype) or telephone. The intervention – ‘iSupport’ – will be hosted by the Pan American Health Organisation (PAHO), the regional office of the World Health Organization (WHO) for the Americas.

For the trial, carers who meet the inclusion criteria, consent to take part, and are randomised to the intervention arm will be provided with access to the platform by the research team for six months. They will be able to access it at their own pace and time from wherever they feel is convenient.

Analysis plans relevant to the WSs will be written, scrutinised and agreed before recruitment has been completed for all quantitative analyses. This will ensure that variables potentially contributing to missing data will be considered a priori. Independent committees will have the opportunity to comment on these plans.

**4.3.1 Research sites**

Bangor University is the lead research site for this study and researchers working for this institution will lead the Welsh arm of the study. University College London and the University of Strathclyde are collaborating research sites. Researchers from these institutions will lead the English arm and Scottish arm respectively. All researchers will follow the same working procedures, as described in this protocol.

**4.3.2 Organogram of research sites and study reporting**


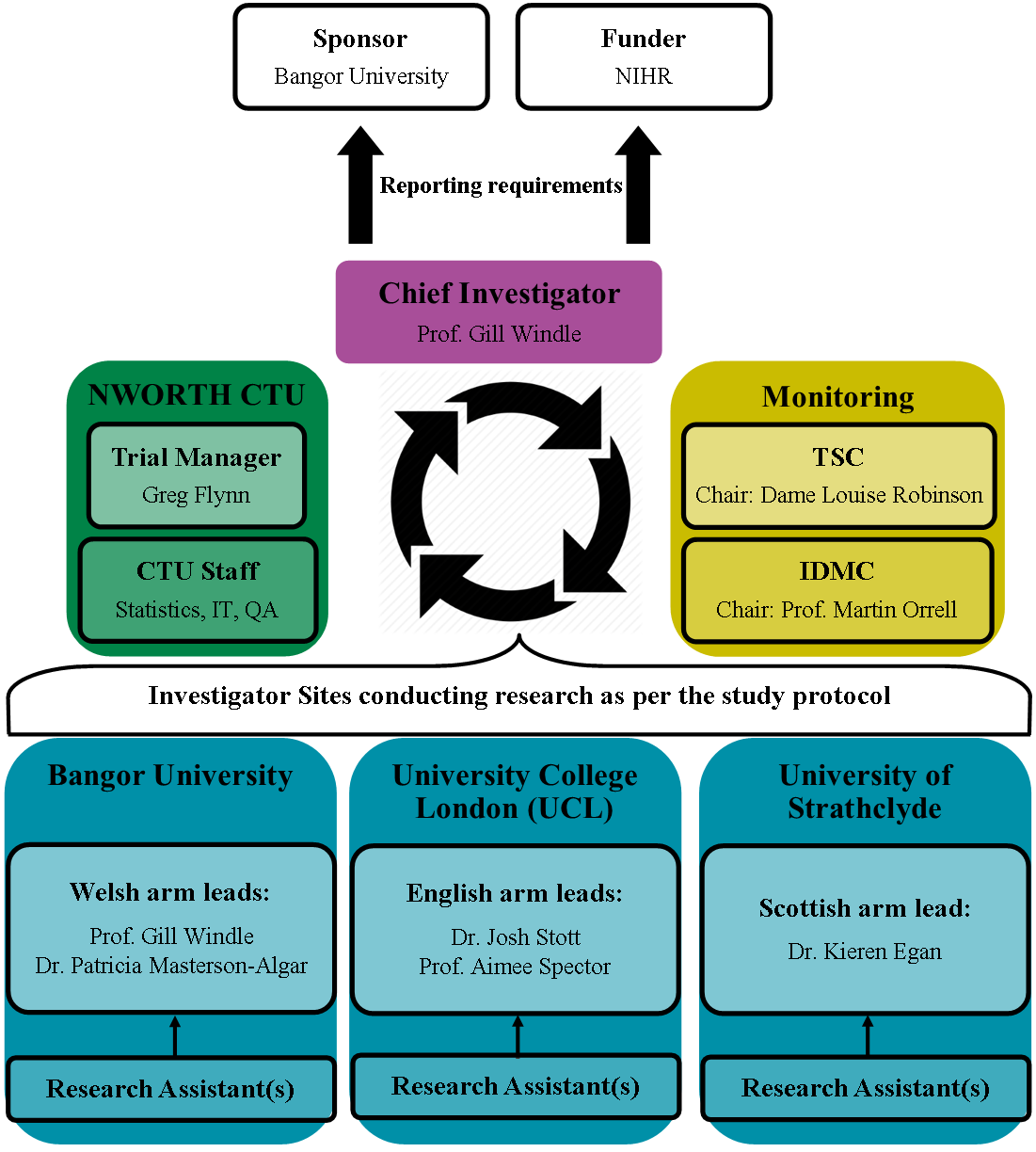


*Figure 4: Organogram of research sites and study reporting*

**4.4 Sampling and sample size**

Both primary outcomes (Zarit Burden scale and CES-D10) are important to the participants and have potential to indicate an effect, a successful trial would be one which detected an effect in either of these outcomes. Therefore, the sample size has been approached considering these as multiple primary endpoints at six months.

The Portuguese RCT of ‘iSupport’ has set an effect size of 0.5^14^ and a meta-analysis of multicomponent interventions for carers found a standardized effect size of 0.65 [CI=0.46-0.84] for the ZBI^23^. Being conservative we have assumed a standardised effect size of 0.4 for the ZBI, which is equivalent to a 4-point difference on the scale and assuming a standard deviation of 10 as derived from scale validation with dementia carers.^^[[29]](#endnote-29)^^

Meta-analysis by Leng et al.^^[[30]](#endnote-30)^^ indicated that the standardised effect size possible for the CES-D10 would be in the order of 0.2. Ying et al.^^[[31]](#endnote-31)^^ denote that the correlation between these two measures is approximately 0.7.

Using the multiple primary endpoint estimator in the R package mpe with power of 90% and significance set to 2.5% established a sample of 262 would be required to have the potential to detect an effect in at least one of these outcomes. The mpe package uses the methodology of Sugimoto et al.^^[[32]](#endnote-32)^^ and Suzo^^[[33]](#endnote-33)^^ to estimate the sample size required based on the defined effect sizes and the correlation between the measures. The attrition rate is estimated as 25%, based on 9 dementia intervention studies, where the mean retention rate was 15.33% (range 2%-24%). Accommodating a 25% attrition rate by six months, we will need to recruit and randomise 350 participants.

For WS2, sociodemographic factors collected at baseline will inform the purposive sampling strategy. The choice of sample size in qualitative research is an area of debate.^^[[34]](#endnote-34)^^ The sample size (up to n=50) will be determined by thematic saturation, along with pragmatic considerations, e.g. it is recommended that studies employing individual interviews undertake no more than 50 interviews in order to manage the complexity of the analysis.^^[[35]](#endnote-35)^^ The purposive sampling will include a diverse range of participant characteristics such as age, gender, ethnicity, location, caring responsibilities, as well as the extent to which they used/didn’t use ‘iSupport’ and level of support from the ‘e-coach’. Motives for declining participation will also be noted where consent is given, to understand barriers for participation and selection bias.

To address possible socioeconomic inequalities, we intend to collect the following

information:

- The number of people who express an interest in the trial but are unable to take part because they do not have access to the internet.
- The number of people who express an interest in the trial and have access to the internet but are unable to take part as their internet is unreliable.
- The number of people who express an interest in the trial but are unable to take part because they do not have a PC, tablet or smart phone.
- The number of people who express an interest in the trial but do not join as they feel their IT skills are not sufficient.
- The number of people who express an interest in the trial but are unable to take part because the intervention languages are not compatible with their first language.
- Anonymised data on the age, gender and ethnicity of people who express interest in the trial but do not consent to take part.

**4.5 WS1 Randomised controlled trial**

WS1 is a multi-centre, pragmatic, single-blinded, two-arm randomised controlled trial (with a nested internal pilot). It will evaluate the effectiveness of ‘iSupport’ in reducing carer distress and symptoms of depression (primary outcomes). Secondary outcomes will assess reductions in anxiety, and improvements in resilience, relationship quality and dementia knowledge. Assessments will be completed at baseline (T0), 3 months after baseline (post-intervention, T1), and 6 months after baseline (follow-up, T2). NWORTH will provide a randomisation system maintained by a team independent of the trial. Randomisation will use a secure web-based dynamic adaptive randomisation algorithm^22^ and be stratified for site along with age and gender, previously found to influence the outcome measure of caregiver distress.^23^ If more than one person identifies as the carer for the same person (e.g. spouse/partner, adult child, or friend) we would allocate one person as the ‘index carer’ (based on caring frequency, or if caring frequency is equal between carers, then the carers will nominate who will be ‘index carer’).

**4.5.1 Internal pilot study**

A six-month internal pilot will be nested in WS1 at each site. Progression criteria will be assessed as a whole, and will guide decisions on a go/review/stop basis. A successful outcome of the internal pilot would be to have all criteria assessed as go. Continuation will still be possible with a combination of stop, review and go flags, but will require additional discussion within the Trial Steering Committee (TSC), research team and the funder prior to proceeding (to mitigate risks highlighted in the pilot study). The discussions would consider the overall context in which the criteria have been assessed, and if a decision is reached which indicates that either the design or processes need to be overhauled, this might suggest termination of the trial. Termination would be fully discussed in collaboration with the funder and independent committees. All thresholds have been set based on levels that would enable completion of the trial objectives within the proposed timeframe (Go without adaptation, Review with adaptations to trial processes, Stop may not be possible to complete).

- Recruitment and set up/ training of sites within time allocated: Go: 3, Review: 2, Stop: 1.
- Recruitment of participants based on target of n=110 by month 6 of recruitment: Go:>= 94 (>=85%), Review:55-93 (50 - 84%), Stop:<55 (<50%).
- Retention of recruited participants to 6 months, assessed as a percentage of those who should have reached 6 months at the time of internal pilot assessment: Go:>=75%, Review:40-74%, Stop:<40%.
- Acceptability of intervention: assessed by utilisation of ‘iSupport’ (the number of participants who have logged in and used the system more than once): Go:>=70%, Review:50-69%, Stop:<50%.
- Ability to collect outcome data (assessed on baseline and first follow-ups only). A measure would be a candidate for removal if less than 85% of participants attempt to complete a measure: Go:>=85%, Review:70-84%, Stop:<70%. This only becomes a trial termination criteria if this were in relation to the primary outcome. Missing data within an outcome measure will be assessed separately.

**4.5.2 Selection of participants**

Potential participants who express an interest in the project but do not meet the inclusion criteria will be provided with information about relevant organisations (including our own support groups) and a copy of the ‘iSupport’ manual.

Participants who meet the inclusion criteria for WS1, provide informed consent and complete the baseline assessments (see section 4.5.5 Data collection for further details), will be randomised and the outcome communicated to them in an email, internet-based service (e.g. Zoom, Teams or Skype) or telephone by the Trial Manager or Chief Investigator. Participants randomised to the ‘iSupport’ group will receive the intervention log-in details and they will have access to the intervention for 6 months. To help retention, participants in the comparison group will be given access to ‘iSupport’ at the end of the data collection. Excluded participants will also be able to use ‘iSupport’ after study completion.

At the end of the trial, both intervention and comparison groups will receive information on national and regional organisations that can provide help, such as those provided by our charity partners, and information about support groups that our respective institutions (UCL and Bangor) host. To aid recruitment and retention in line with suggestions from Carers Trust Wales, all eligible randomised participants will be reimbursed with a gift voucher to thank them for their contributions.

**4.5.3 Primary outcome measures**

Reflecting the intentions of the intervention, the outcome measures assess reductions in psychological morbidity and the promotion of personal capabilities to mitigate against morbidity.

There will be two primary outcome measures for WS1. Sample size has been based on considering both outcomes as primary outcomes where a successful trial would be noted if at least one of the outcomes indicated a statistically significant effect. This choice is justified as a meta-analysis evaluating the efficacy of technology-based interventions for informal carers of people living with dementia found they had a significant effect on reducing depression and burden outcomes.^^[[36]](#endnote-36)^^

The first primary outcome measure will assess reductions in carers’ distress, measured by the 12-item Zarit Burden Interview.^18^ Item responses range from 0 (never) to 4 (almost always), and higher scores indicate greater distress. The original 22-item ZBI is used widely in research with dementia caregivers and internal consistency of the 12-item version, as measured by Cronbach’s alpha, is α =.85.^^[[37]](#endnote-37)^^ Concurrent validity of responses to the 12-item version has been examined and found to be good relative to indices of patient behavioural disturbance and ADL impairment in dementia.^18^ The ZBI-12 is considered valid for evaluation of burden in clinical practice and research as a fast, efficient option for screening burden among older caregivers of community-dwelling older adults.^^[[38]](#endnote-38)^^

The second primary outcome measure is the Centre for Epidemiological Studies of Depression Scale (CES-D10),^20^ a very widely used 10-item measure of depression. Ratings relate to the past week with eight items measuring frequency of depressive symptoms and two measuring positive affect. Response categories range from 0 (rarely or none of the time present) to 3 (most or all of the time present). Scores range from 0 (no depression) to 30 (very depressed). Internal consistency ranges between Cronbach α=.86 -.88 in an older caregiver population.^20^ CES-D is a valid and reliable scale for detecting caregiver depression in dementia. It has added utility, beyond that of a caregiver burden scale, in identifying a subgroup of caregivers with depression but not burden.^^[[39]](#endnote-39)^^

**4.5.4 Secondary outcome measures**

Generalised Anxiety Disorder Questionnaire (GAD-7)^^[[40]](#endnote-40)^^ is a widely used 7-item measure rating the frequency of common symptoms of anxiety in the past two weeks. Response categories range from 0 (not at all) to 3 (nearly every day). Scores of 15 indicate severe anxiety. GAD-7 has excellent internal consistency (Cronbach α=.92), good sensitivity, is a valid and reliable measure for detecting generalised anxiety disorder in the general population. It has been used with carers of people living with dementia.^^[[41]](#endnote-41)^^ GAD-7 was selected by the NHS England Improving Access to Psychological Therapies (IAPT) programme as the gold-standard measure of anxiety.^^[[42]](#endnote-42)^^

Following the recommendations of a 12-country European working group to include measures of ‘living as well as possible’ with dementia,^^[[43]](#endnote-43)^^ improvements in the way the carers perceive they can manage the situation will be assessed through the Resilience Scale-14
(RS-14).^^[[44]](#endnote-44)^^ Derived from the original 25-item Resilience Scale, this 14-item version is strongly correlated with the original (r=0.97, p>0.001) and has an excellent internal consistency ranging from Cronbach α = .89 to .96. Response options range from 1 (Strongly disagree) to 7 (strongly agree). Higher scores are indicative of resilience level. Construct validity has been established in a wide range of previous research and it has been used in previous research with dementia caregivers.^^[[45]](#endnote-45)^^

We will examine the influence of ‘iSupport’ on the quality of the caregiving relationship, using the quality of the carer-patient relationship (QCPR).^^[[46]](#endnote-46)^^ The QCPR is a measure of relationship quality, comprising 14 items designed to assess warmth, levels of conflict and criticism in the caregiving relationship. Previous studies have shown that the QCPR has good internal consistency and concurrent validity and it has been used in relation to online interventions for dementia carers.^36^

Improvements in how the carer understands their relative will be assessed with a measure of dementia knowledge (DKAS).^^[[47]](#endnote-47)^^ The 25-item measure exhibits good reliability (α = .85; ωh = .87; overall scale), with acceptable subscale internal consistency (α ≥ .65; subscales). Subscales showed acceptable correlation without any indication of redundancy. Total and DKAS subscale scores show good discrimination between cohorts of respondents who would be anticipated to hold different levels of knowledge on the basis of education or experience related to dementia.

To ascertain the impact on the health-related quality of life of the person being cared for, we will use the DEMQOL-Proxy. This is a widely-used instrument for measuring the health-related quality of life of people living with dementia, completed by the carer. It is adapted for use as a preference-based measure in economic evaluations.^^[[48]](#endnote-48)^^

**4.5.5 Data collection**

The primary mode of data collection for all the outcome measures will be technology mediated, i.e. interviews over an internet-based service (e.g. Zoom, Teams or Skype) or telephone. The outcome measures will be collected first on paper Case Report Forms (CRFs) and then entered into an online data management system (MACRO) by a member of the research team. Assessments will be done at baseline (T0), 3 months after baseline (T1), and 6 months after baseline (follow-up, T2). Demographic data will be collected at baseline (e.g. age, gender, marital status, ethnicity, education, occupation, length and frequency of caring, dementia diagnosis of family member). The researcher will also collect the country/area specific COVID-19 restrictions at the time of data collection.

Whilst every effort will be made to follow-up participants as close as possible to the defined time point this may prove difficult. In these instances T1 and T2 data collection would be acceptable 2 weeks early and up to 4 weeks late. Date of data collection will be recorded in the CRF.

**4.5.6 Data analysis**

Primary analysis will be conducted on an intention to treat (ITT) basis, blinded to treatment allocation. The primary assessment for effectiveness will be adjusted estimates of the ZBI scores and CES-D10 scores between the two groups assessed at 6 months. A linear mixed effects model adjusting for baseline scores, randomising site (random effect) and stratification variables will be fitted for each of the two primary outcomes. Similar models will be fitted for all continuous secondary outcomes.

All estimates of effect will be presented together with 95% confidence intervals. The aim is to minimise missing data; however, predictors of missingness will be investigated using regression models and any predictors found will be considered for inclusion in the models. Multiple imputation will be employed to address missing scores where appropriate. A sensitivity analysis will be utilised to assess whether there is any impact resulting from participants completing outcome measures in Welsh.

A full Statistical Analysis Plan will be written and agreed before completion of data collection. The independent committees will have the opportunity to comment on this plan. If any deviations from the planned statistical analysis are required these will be fully documented and justified in the final analysis report.

**4.6 WS2 Process evaluation**

Process evaluation will run alongside WS1 and will apply mixed-methods (semi-structured interviews, quantitative questionnaires and analysis of data from the online platform). It will be conducted in line with established guidance frameworks.^15,16^ It will examine throughout the intervention period how participants engage with and adhere to particular aspects of ‘iSupport’ (e.g. most/least frequently visited pages, the most ‘popular’ modules/sessions, sessions with quizzes with the highest rates of wrong responses).

Change mechanisms will be investigated by exploring the barriers, facilitators and contextual factors which influence the uptake and implementation of ‘iSupport’ (i.e. the sociodemographic diversity of participants). The extent to which ‘iSupport’ may have changed behaviours beyond the intervention (e.g. help-seeking) will be explored, as will the extent to which it is beneficial in the current circumstances of distancing and isolating in an ongoing or future repeated pandemic such as COVID-19.

**4.6.1 Quantitative data collection**

A System Usability Scale (SUS)^^[[49]](#endnote-49)^^ will be administered at 6-month follow-up. This 10-item scale will quantitatively evaluate the overall usability of the ‘iSupport’ platform. Each item is a statement (e.g. “I thought ‘iSupport’ was easy to use”) and responses given on a 5-point Likert scale 0 (strongly agree) to 4 (strongly disagree). Total scores range from 0 to 40 which are then converted to 0-100 and normalised to produce a percentile ranking. The SUS is easy to administer, reliable and valid and effectively differentiates between usable and unusable systems. To avoid unblinding the research assistants, they will contact the Trial Manager following the 6-month follow-up. The Trial Manager will send out a thank-you email with a link to the SUS and other questions about using iSupport, which will be self-completed online (e.g. through Qualtrics or Survey Monkey).

WS2 will collect data from the online platform regarding usability (e.g. frequency and length of use, which modules/ lessons / pages users most frequently visit; average length of time spent on each module / lesson / page per user; from tablet or PC). The number of contacts with the ‘e-coach’ will also be recorded.

**4.6.2 Qualitative data collection**

Semi-structured interviews will be undertaken using an internet-based service (e.g. Zoom, Teams, Skype, or GoToMeeting) or telephone, with a sub-sample of the intervention participants. These will be recorded and professionally transcribed. The topic guides will be guided by the process evaluation parameters described in recognised frameworks,^15,16^ and drawing upon theoretical models such as Normalisation Process Theory (NPT).^^[[50]](#endnote-50)^^ They will be developed in partnership with the PPI group and our collaborators. Regular meetings will be held with the Research Assistants and ‘e-coach’ to identify any new questions which arise from emerging themes.

**4.6.3 Data analysis**

Quantitative data from the online platform will be analysed descriptively by calculating total numbers, percentages, means and standard deviations, or, the median and range if not normally distributed. This will provide information on the intervention. Descriptive statistics of sociodemographic characteristics of intervention participants will demonstrate the reach of the project and will then be compared with Office For National Statistics data to preliminarily investigate sample representativeness.

Interview data will be recorded either by the videoconferencing software, or via an encrypted digital recorder and then professionally transcribed verbatim. Transcripts will be checked for accuracy against the recordings and any corrections made. The researchers will re-read all transcripts to gain familiarity with the data which will then be coded, informed by a coding framework based on proposed/hypothesised mechanisms identified in the study’s logic model (see Appendix 1) and informed by NPT.^47^ Analysis will follow the phases of thematic analysis by Braun and Clarke^^[[51]](#endnote-51)^^ using NVivo. This analysis will reveal the experiences of ‘iSupport’ and its delivery, the barriers and facilitators to its uptake and continued use, and the perceived benefits for the carer participating in ‘iSupport’ and for the person they are caring for and how were these realised (mechanism of change).

Results will also be applied to aspects of the ‘Context and Implementation of Complex Interventions’ (CICI) checklist^17^ to generate recommendations for the WHO that pay particular attention to the contextual factors (e.g. personal characteristics) that may influence, or be influenced by the trial setting (e.g. online access) and their relationship with the trial recruitment and intervention delivery, which may reflect implementation in a real world setting (see Appendix 1).

**4.7 WS3 health economics**

WS3 will collect health economics data to calculate the cost-effectiveness of ‘iSupport’. Cost-effectiveness analysis will be undertaken from two perspectives; the base case analysis will adopt a public sector perspective (NHS, personal social services and local authorities) in line with NICE public health guidance,^^[[52]](#endnote-52)^^ and a secondary analysis will be undertaken from a societal perspective using an opportunity cost method. Here, the value of the carer’s next best use of time is calculated (the value of their leisure time or paid employment) to ascertain changes in employment hours (productivity losses) due to caring. If the internal pilot phase of WS1 indicates <60% of carers provide sufficient information on these two indicators to use this method, we will use the proxy-good method. Here a market price for substitute labour to carry out care-related tasks is applied to informal care hours. Out of pocket expenses, such as travel expenses, will be captured through a Service Use questionnaire developed for ‘iSupport’.

**4.7.1 Outcome measures**

The primary outcome measure for the cost-effectiveness will be Quality-Adjusted Life Years (QALYs) at T2. Utility values for the QALY will be obtained from responses to the EQ-5D-5L^^[[53]](#endnote-53)^^ at T0, T1 and T2. The EQ-5D-5L is a generic, preference based, health-related quality of life (HRQoL) measure widely applied in economic evaluation, and in dementia research with both people living with dementia and dementia caregivers. It consists of two parts, a five-item questionnaire and a visual analogue scale (EQ-VAS). The first part asks the respondent about the level of difficulty they have in the following domains: mobility, self-care, usual activities, pain / discomfort, anxiety / depression. The second part asks respondents to rate their overall health using a visual-analogue scale, where health is rated anywhere between 0 (worst imaginable health) and 100 (best imaginable health). Previous studies have shown that the EQ-5D-5L has a good construct validity and good reliability compared with two dementia specific measures.^^[[54]](#endnote-54)^^

As mentioned in WS1 secondary outcome measures, the DEMQOL-Proxy will be adapted for use as a preference-based measure in economic evaluations.^45^

Resource use data will be collected using a study-specific Service Use questionnaire at T0, T1 and T2. Some examples of the topics carers will be asked to report on are their own frequency of contacts with health and social care professionals and that of the person that they care for. We will ask carers to report on the use of respite care and sitting services to allow us to consider the impact of ‘iSupport’ on the person being cared for. Health and social care service use will be costed using national unit costs.^^[[55]](#endnote-55)^,^[[56]](#endnote-56)^^ To incorporate opportunity costs, we will ask carers to report on changes in employment hours (productivity losses) due to caring, employment status and hours, income (including carers allowance and attendance allowance), hours spent caring, types of care tasks undertaken, whether people carry out paid/unpaid care, and carers’ willingness to pay for more support or more leisure time. The Erasumus iMTA informal care questionnaire will be adapted for this aspect.^^[[57]](#endnote-57)^^ The cost of technical support for ‘iSupport’ over the intervention period will be calculated. Records will be kept of the time spent supporting carers to use the tool, and the staff costs associated with this activity will be calculated.

**4.7.2 Data collection**

See section 4.5.5 Data collection (collected in same CRF as for WS1).

**4.7.3 Data analysis**

WS3 will adopt an intention to treat approach. A scoring algorithm using UK tariff values^^[[58]](#endnote-58)^^ will be used to convert carer EQ-5D-5L responses into an index score of between -0.594 and 1, with 1 representing full HRQoL. Care recipient utility values will be derived using the DEMQOL-proxy scoring algorithm. These index values will then be used to calculate QALYs. An appropriate regression model will be used to adjust for imbalances in baseline utility. Cost and QALY data will be combined to calculate an incremental cost-effectiveness ratio (ICER). As the intervention follow-up period is less than 1 year it will not be necessary to discount costs. The nonparametric bootstrapping approach^^[[59]](#endnote-59)^,^[[60]](#endnote-60)^^ will be used to determine the level of sampling uncertainty surrounding the mean ICER by generating 5,000 estimates of incremental costs and benefits. Cost effectiveness acceptability curves^^[[61]](#endnote-61)^^ will be produced to show the probability that ‘iSupport’ is cost-effective compared to standard care for a range of willingness-to-pay thresholds. Secondary cost-effectiveness analyses will calculate the cost per unit change in carer distress using the 12-item Zarit Burden Interview^18^, and cost per unit change in carer anxiety and depression using the 10-item CES-D^20^. A subgroup analysis will be conducted on the number of times that carers in the intervention group access ‘iSupport’ (low/ moderate/ high user categories to be classified during the internal pilot). Sensitivity analyses will be conducted to vary the costs of inputs (e.g. the cost of the staff supporting carers to use ‘iSupport’). The economic evaluation will be reported according to the Consolidated Health Economic Evaluation Reporting Standards.^^[[62]](#endnote-62)^^

A full Health Economics Analysis Plan (HEAP) will be written and agreed before completion of data collection. The independent committees will have the opportunity to comment on this plan. If any deviations from the planned analysis are required these will be fully documented and justified in the final analysis report.

**5. WS4 Feasibility study objectives and design**

**5.1 WS4 Feasibility study objectives and design**

The feasibility study (WS4) is a non-randomised feasibility study of intervention refinement for younger dementia carers. This will:

- Explore the potential of ‘iSupport’ to address the required support that is unique to young carers, including the potential of the platform in the face of the ongoing or future COVID-19 pandemic.
- Work with young carers to refine ‘iSupport’ to fit their needs.
- Identify what outcomes are most important and relevant to young carers in relation to ‘iSupport’.
- Identify the best ways to increase the accessibility and uptake of ‘iSupport’ for young carers.
- Explore the feasibility of the refined ‘iSupport’ intervention.

**5.2 WS4 Research questions**

1. Is it feasible, useful and acceptable to digitally deliver a refined ‘iSupport’ to young carers?

2. What are the carers’ perspectives of ‘iSupport’ in relation to supporting them in an ongoing or future repeated pandemic such as COVID-19?

**5.3 WS4 Feasibility study expected duration**

The total time scheduled for the WS4 feasibility study is 36 months. Key milestones will be monitored as part of overall project management.

**5.4 WS4 Feasibility study flowchart**


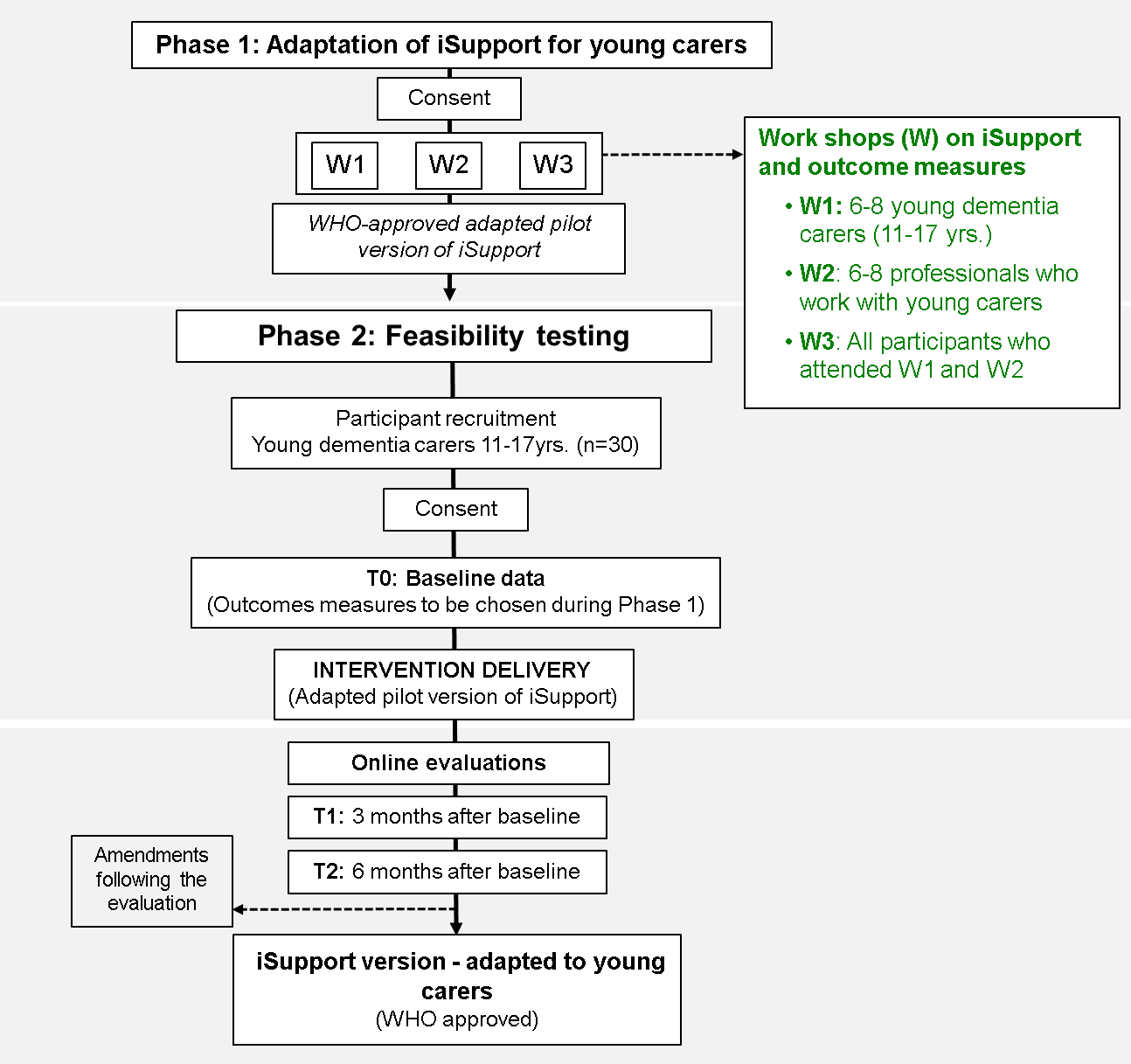


*Figure 5: Feasibility study flowchart including sample sizes*

**6. Selection and withdrawal of WS4 feasibility study participants**

Young carers (ages 11 - 17) will be recruited through stakeholder and research teams’ networks (including secondary schools), social media, and national carers associations (e.g. Carers Trust).

**6.1 WS4 Inclusion criteria**

1) Young people between the ages of 11 - 17 (secondary school age) who self-identify as a carer of a person with dementia who is not living in a full-time care facility, caring at least weekly for at least 6 months.

2) The care recipient has to have a confirmed diagnosis of dementia (through self-report of the carer, to reflect the ‘real world’ application of ‘iSupport’).

**6.2 WS4 Exclusion criteria**

1) Receiving treatment from Child and Adolescent Mental Health Services (CAMHS) at the time of recruitment.

2) Unable to comprehend written English.

3) No access to the internet.

4) Have previously used ‘iSupport’ materials (in the last 12 months).

**6.3 WS4 Feasibility study consent procedure**

For young carers between the ages of 11 - 15, consent will be taken from the parent or legal guardian. Young carers aged 16 or 17 can provide consent independently. Age-specific documentation has been produced, and researchers taking consent will be trained on which participant information sheet to use.

Please see section 3.3 Trial consent procedure.

**6.4 WS4 Randomisation and unblinding**

As this is a non-randomised feasibility study, randomisation and unblinding are not applicable.

**6.5 WS4 Withdrawal of participants**

Please see section 3.6 Withdrawal of participants.

**7. WS4 Feasibility study procedures**

This is a refinement and feasibility study of ‘iSupport’ for young carers, delivered in two phases: Phase 1 (adapting the intervention) applies principles of co-design reflecting co-applicant Masterson-Algar’s expertise.^^[[63]](#endnote-63)^^ It will involve three sequential co-design workshops with young carers and professionals; Phase 2 (feasibility testing) will then explore the refined ‘iSupport’ from phase 1.

**7.1 WS4 Planned intervention**

The intervention – as laid out in section 4.1 Planned intervention – will be adapted for the specific needs of young carers as explained below.

**7.2 WS4 Setting and context**

The research will be undertaken with young carers who live in Wales, Scotland or England. Researchers will recruit and assess participants in a one-to-one meeting using an internet-based service (e.g. Zoom, Teams or Skype) or over the telephone. The adapted version of the intervention will be hosted by the PAHO/WHO.

**7.3 WS4 Feasibility study**

**7.3.1 Phase 1: Adaptation of ‘iSupport’ for younger dementia carers**

Phase 1 will follow WHO adaptation guidelines to tailor the programme for young carers. This will consist of three workshops. Depending on the government guidelines regarding COVID-19 and safety, workshops will either be held in-person at Bangor University, or they will be technology mediated i.e. using an internet-based service (e.g. Zoom, Teams or Skype). The first (Workshop 1) will explore with 6-8 young carers, their experiences of caring, what is important to them and how this might be reflected (or not) in ‘iSupport’. They will provide in-depth feedback on the content and style of each of the 5 modules. Workshop 2 will undertake a similar exercise with 6-8 professionals who work with young carers. The refined ‘iSupport’ will be shared in Workshop 3 with all participants who attended the first two workshops, along with discussion regarding outcomes and measures. Modifications will be made before being sent to WHO for approval.

The workshops will draw on participants’ experiences, using creative activities to stimulate and encourage reflectivity. These activities will simultaneously engage, reveal tacit knowledge and generate ideas. Participants will be given access to ‘iSupport’ at least 2-weeks before the workshops and will be instructed to note what is useful, what is clearly explained (or not), what could be made better, the extent to which it is beneficial in circumstances of isolating and distancing, etc. They will also be provided with a printed version of ‘iSupport’ content for them to make further annotations. They will identify what outcomes are most important to young carers in relation to ‘iSupport’, using the measures in the trial as discussion points. Following this, the final WHO-approved version will be produced for phase 2 (feasibility testing).

**7.3.2 Phase 2: Feasibility testing ‘iSupport’ for younger dementia carers**

The WHO-approved version will test the feasibility of the refined ‘iSupport’ intervention with a group of 30 young dementia carers. Outcome measures selected as important in Phase 1 will be used to collect data, as per the data collection process for WS1 of the trial (see section 4.5.5 Data collection), with some new outcome measures for young people and questions relating to demographic information tailored for younger carers (e.g. how many older siblings do you have, how many younger siblings do you have, do you go to school/college).

During this phase we will also aim to develop an understanding of recruitment pathways and explore barriers and enablers to recruitment for a future definitive trial. This is particularly important as young dementia carers are a ‘hard to reach’ group. Parents of young carers and professionals who work with young carers will be invited to semi-structured interviews and/or focus groups.

**7.3.3 Sampling and sample size**

No formal sample calculation has been conducted due to the nature of this feasibility study. However, informed by the methodological framework proposed by Lancaster et al.,^^[[64]](#endnote-64)^^ it is envisaged that a sample of n=30 for phase 2 will provide enough information on the acceptability of the intervention, the appropriateness of data collection forms, the feasibility of recruitment and consent procedures and the most appropriate primary outcome measure. Hence, indicating whether further investigation of the developed intervention is warranted.

For the qualitative interviews and/or focus groups to explore Phase 2 recruitment pathways, barriers and enablers, a sample of n=5-10 participants will provide enough information

**7.3.4 Selection of participants**

Young carers and their parents will be recruited through stakeholder and research teams’ networks (including secondary schools), social media, and national carers associations (e.g. Carers Trust).

Professionals will be recruited through stakeholder and research teams’ networks (including secondary schools), social media, local authorities, national carers associations (e.g. Carers Trust). To be eligible they will need to be professionals who, as part of their professional role, have regular contact with young people and young carers (e.g. teaching staff involved in pastoral care, young carer charity workers and social workers in children’s services.)

**7.3.5 Data collection**

Phase 1 workshops will either be held in-person at Bangor University, or they will be technology mediated i.e. using an internet-based service (e.g. Zoom, Teams or Skype) depending on the government guidelines regarding COVID-19 and safety. Workshops will be video recorded and will be approximately 3 hours long. PPI co-applicant Hughes is a fluent Welsh speaker and will co-facilitate all workshops. Decisions on outcome measures for phase 2 will be informed by data collected during Phase 1.

Phase 2 will be conducted as per the data collection process for WS1 of the trial (see section 4.5.5 Data collection). Participants will also be asked to complete an online evaluation of their experiences using ‘iSupport’, similar to that of WS2 (see section 4.6.1 Quantitative data collection).

In order to explore Phase 2 recruitment pathways, one to one semi-structured in-depth qualitative interviews as well as a focus group will be undertaken using an internet-based service (e.g., Zoom, Teams or Skype). Participants (n=5-10) will include parents of young dementia carers and professionals working closely with young carers, for example as part of young carers support projects across the UK. Both, the interviews and the focus group will be recorded (the focus group will also be video recorded) and professionally transcribed. The topic guides will be informed by Phase 1 results as well as parameters described in recognised frameworks^15,16^.

**7.3.6 Data analysis**

Data from Phase 1 workshops will be selectively transcribed, and hand-written field notes will be converted into electronic text. Masterson-Algar and the research assistant will re-visit the recordings and take written notes to gain familiarity with the data. This qualitative data will inform modifications to ‘iSupport’ with the aim to make it more relevant for younger carers. The work will be undertaken in line with the FRAME^^[[65]](#endnote-65)^^ and with the WHO adaptation and implementation guide (2018).

All quantitative data collected during phase 2 will be presented descriptively. No inferential testing will be undertaken for this feasibility data. The mean change from baseline, associated variances and 95% confidence intervals will be calculated for all selected outcomes. Consideration will be given to the applicability of these outcomes for development into a protocol for a defined randomised controlled trial (RCT) if the acceptability of the intervention is proven. Success will be defined as acceptability of the recruitment and consent procedure, data collection tools, intervention content and delivery to participants, as well as compliance.^64^ An estimation of the precision of the means and variances will be made to inform the power calculation for a future RCT protocol.

The online evaluation in phase 2 will include Likert-like and open-ended questions and will be informed by NPT and the mechanisms of change through the application of frameworks.^15,16^ It will aim at exploring young carer’s thoughts about the content, accessibility and perceived benefits of the refined version of ‘iSupport’.

The analysis of Phase 2 qualitative data from interviews and the focus group will follow the phases of thematic analysis by Braun and Clarke^51^. This analysis will reveal barriers and enablers to recruitment as well as identify recruitment pathways and perceived factors impacting on the uptake and acceptability of support (such as iSupport for young dementia carers) by young dementia carers.

**8. Translating ‘iSupport’ into Welsh**

We will follow the WHO standardised guide for translation and adaptation to translate ‘iSupport’ into the Welsh language for WS1. Following professional translation the full text will be independently checked by two experts in the field (already known to the research team). This may lead to suggestions for minor modifications following the original translation. Following any modifications the subsequent procedures will be applied.

Up to 10 caregivers and 6 professionals (Welsh speaking) will be recruited following the procedures outlined for WS1. They will be invited to share their expertise by working through the Welsh version of ‘iSupport’. They will be provided with prior guidance by the researcher through either a group meeting or individually, depending on their availability. They will be asked to individually go through ‘iSupport’, examine the exercises and write down their opinions about content that needs attention. Following this phase the researcher will convene either a group meeting or meet each person individually to discuss their suggestions and send any final modifications to the WHO. All meetings will be undertaken using an internet-based service (e.g. Zoom, Teams or Skype). The final version of the Welsh ‘iSupport’ will be implemented in WS1.

**9. WS5 Feasibility testing the Bengali adaptation of iSupport**

WS5 will explore the feasibility of a Bengali language ‘iSupport’. WS5 is being led by ‘iSupport’ Study collaborator Aimee Spector at UCL. WS5 received additional funding from the NIHR, awarded under a variation to contract to the initial research programme (Reference NIHR 130914).

**9.1 WS5 Feasibility study objectives and design**

This is a non-randomised feasibility study of the translated and culturally adapted Bengali version of iSupport. This will test, both quantitatively and qualitatively; the feasibility of recruiting people to the intervention, retention to the study, attrition, acceptability of the intervention, feasibility of the selected outcome measures and potential changes following the intervention.

**9.2 WS5 Research questions**

1. Is it feasible, useful, and acceptable to digitally deliver a translated version of ‘iSupport’ for Bengali-speaking carers?

2. What are the carers’ perspectives of ‘iSupport’ in relation to supporting them in an ongoing or future repeated pandemic such as COVID-19?

3. What are the barriers and facilitators to engaging Bengali carers in the ‘iSupport’ intervention?

**9.3 WS5 Feasibility study expected duration**

The total time scheduled for the WS5 feasibility study is 12 months. Key milestones will be monitored as part of overall project management.

**9.3.1 WS5 Feasibility study Flowchart**

*Figure 6: WS5 study flowchart including sample sizes*

**9.4 Selection and withdrawal of WS5 feasibility study participants**

Bengali-speaking carers will be recruited through our project collaborators’ (e.g. Carers Centre Tower Hamlets, Age UK Lancashire, Lancashire BME Network, and Sandwell Dementia Community Support Service) via their networks and social media.

Additionally, community and religious leaders and professionals working with South Asian dementia carers (e.g. working as part of a carers support project across the UK) will be invited to take part in semi-structured qualitative interviews and/or a focus group.

Participants are free to withdraw at any time during the trial without any impact on their future health and care. Participant data collected to the point of withdrawal will be used in the analysis set unless consent for this is specifically withdrawn.

**9.5 WS5 Inclusion criteria**

1) Bengali speaking adults (18+) who self-identify as an unpaid carer (partners, children, friends, etc.) of a person with dementia who is not living in a full-time care facility, caring at least weekly for at least 6 months.

2) Self-identify as experiencing at least some stress, depression, or anxiety.

3) The care recipient must have a confirmed diagnosis of dementia (through self-report of the carer, to reflect the ‘real world’ application of ‘iSupport’).

**9.6 WS5 Exclusion criteria**

1) Receiving psychological treatment from a mental health specialist at the time of recruitment.

2) No access to the internet.

3) Unable to give informed consent to the trial.

4) Have previously used ‘iSupport’ materials (in the last 12 months).

**9.7 WS5 Feasibility study consent procedure**

Please see section 3.3 Trial consent procedure. If WS5 participants are recruited in-person (e.g. while attending a project collaborator’s group), informed consent will be taken in-person in line with the Trial consent procedure and Good Clinical Practice (GCP).

**9.8 WS5 Randomisation and unblinding**

As this is a non-randomised feasibility study, randomisation and unblinding are not applicable.

**9.9 WS5 Withdrawal of participants**

Please see section 3.6 Withdrawal of participants.

**9.10 WS5 Feasibility study procedures**

The WHO-approved version of the adapted Bengali-language ‘iSupport’ will be used by 25 Bengali-speaking dementia carers for three months to test if it is feasible to conduct a larger-scale trial of the translated intervention.

Measures will be translated and culturally validated versions of the outcomes used in the main ‘iSupport’ trial, or where these are not available alternate measures of the same construct which are culturally appropriate.

The constructs (with example measures) will be:

- Carer burden (e.g. Zarit Burden Interview)
- Depression (e.g. Center of Epidemiologic Studies Depression Scale 10-item version)
- Anxiety (e.g. General Anxiety Disorder-7)
- Quality of life (e.g. EQ-5D 5-level version, WHO-QOL BREF, Cornell-Brown Scale)
- Resilience (e.g. 14-item Resilience Scale, Resilience Scale for Adults)
- Carer-patient relationship (e.g. Quality of Carer–Patient Relationship Scale)
- Dementia knowledge (e.g. Dementia Knowledge Assessment Scale)

Data will be collected at baseline and at 3-month follow up. A sub-sample of up to 15 participants will also undergo semi-structured qualitative interviews (see section 9.14 WS5 Data collection).

We also aim to develop an understanding of recruitment pathways and explore barriers and enablers to recruitment for a future definitive trial. Up to 15 community and religious leaders, and professionals who work with South Asian dementia carers, will be invited to semi-structured qualitative interviews and/or a focus group.

**9.11 WS5 Planned intervention**

The intervention – as laid out in section 4.1 Planned intervention – will be translated and culturally adapted to produce a Bengali version of ‘iSupport’ for dementia carers. This is being led at UCL through PPI and stakeholder engagement.

**9.12 WS5 Setting and context**

WS5 is led by UCL and will be undertaken with Bengali speaking dementia carers who live in the UK. Researchers will recruit and assess participants in a one-to-one meeting either face-to-face, over the telephone, or using an internet-based service (e.g., Zoom, Teams or Skype) depending on participant preference. The adapted version of the intervention used for this work-stream will be developed and hosted by UCL in partnership with the Sponsor.

**9.13 WS5 Sampling and sample size**

No formal sample calculation has been conducted due to the nature of this feasibility study. Twenty-five participants will be selected to take part, and 15 of these recruited carers will be purposively sampled for qualitative interviews following their participation in the feasibility study.

For the qualitative interviews and/or focus groups to explore recruitment pathways, barriers and enablers, we anticipate that a sample of n=10-15 participants will provide enough information to reach data saturation.

**9.14 WS5 Data collection**

The primary mode of data collection for all outcome measures will be technology mediated. Participants will be sent a personalised link to self-complete outcome measures online using an appropriate survey tool licenced by UCL (e.g. Qualtrics). If the participant would prefer to have support answering the questions, a researcher can provide support over video call (e.g. Zoom, Teams or Skype) or telephone.

Assessments will be done at baseline (T0) and 3 months after baseline (T1). Demographic data will be collected at baseline (e.g. age, gender, marital status, ethnicity, education, occupation, length and frequency of caring, dementia diagnosis of family member).

The 3 month follow up (T1) will be as close as possible to the defined time point, with an acceptable window of 2 weeks before or up to 4 weeks after the defined point. Date of data collection will be recorded in the online questionnaire.

Semi-structured qualitative interviews with 15 participants from the feasibility study will be audio recorded. Final topic guides will be co-produced with our study partners, as well as people with dementia and their carers, with the interviews aiming to explore the feasibility, acceptability and cultural appropriateness of the intervention and outcome measures. Interviews will also explore participants’ motivation and perceived barriers and enablers to their involvement in the study.

In order to further explore recruitment pathways, one-to-one semi-structured qualitative interviews and/or a focus group will be undertaken using an internet-based service (e.g., Zoom, Teams or Skype). Both the interviews and the focus group will be audio recorded and transcribed at UCL, and the focus group will also be video recorded.

**9.15 WS5 Data analysis**

All quantitative data collected during WS5 Feasibility study will be presented descriptively. No inferential testing will be undertaken for this feasibility data. The mean change from baseline, associated variances and 95% confidence intervals will be calculated for all selected outcomes. Consideration will be given to the applicability of these outcomes for development into a protocol for a defined randomised controlled trial (RCT) if the acceptability of the intervention is proven. Success will be defined as acceptability of the recruitment and consent procedure, data collection tools, intervention content and delivery to participants, as well as compliance. An estimation of the precision of the means and variances will be made to inform the power calculation for a future RCT protocol.

Data from interviews will be transcribed verbatim, coded, and analysed by two independent researchers using a deductive thematic analytic approach. The framework for analysis will be in line with our aims and explore the perceived feasibility, acceptability, fidelity, and value of the intervention.

The analysis of qualitative interviews with professionals will reveal barriers and enablers to recruitment, as well as identify recruitment pathways and perceived factors impacting on the uptake and acceptability of support (such as iSupport for South Asian carers) by South Asian dementia carers.

**10. WS6 ‘CareFit’ for dementia carers**

WS6 is preparatory research to explore the adaptation, recruitment, feasibility and implementation of a physical activity mobile application (app) called ‘CareFit’ for informal carers of people with dementia. WS6 is being led by ‘iSupport’ Study collaborator Kieren Egan at University of Strathclyde. WS6 received additional funding from the NIHR, awarded under a variation to contract to the initial research programme (Reference NIHR 130914).

**10.1 WS6 research objectives and design**

WS6 is a mixed-methods evaluation of a motivational smartphone app called ‘CareFit’ to support home-based regular physical activity for unpaid dementia carers. WS6 aims to improve current uncertainties around social care pathways to implementation, and gain understanding of facilitators and barriers to the reach, effectiveness, adoption, implementation and maintenance of ‘CareFit’ in a real-world setting.

Building on existing work at University of Strathclyde^^[[66]](#endnote-66)^,^[[67]](#endnote-67)^^, WS6 will:

- Expand an initial 3-week intervention to an 8-week intervention to improve understanding of usage adherence
- Develop understanding of recruitment pathways and explore barriers and enablers to recruitment for a future definitive trial, including recruiting vulnerable/lower socio-economic groups
- Develop understanding of the most reliable methods to regularly measure physical activity and sedentary behaviour within dementia carers
- Explore how ‘CareFit’ could provide added value for dementia carers with the existing solution of ‘iSupport’ through qualitative interviews or focus groups with key stakeholders
- Explore unexpected benefits including whether people with dementia also see benefits of using the intervention

WS6 follows the WHO recommendations for Monitoring and Evaluating Digital Health Interventions^^[[68]](#endnote-68)^^ (see section 10.2 WS6 Research definitions). Additionally, the WHO recommends that a model of evaluation is adopted that supports testing and evaluation under real world conditions. Based on this recommendation, the RE-AIM evaluation framework^^[[69]](#endnote-69)^^ has been chosen based on its alignment to the aims and objectives of the project. The RE-AIM framework supports the collection of data across indicators of five dimensions of a health intervention; reach, effectiveness, adoption, implementation and maintenance.

**10.2 WS6 Research definitions**

| **Term** | **Evaluation definition** | **Example questions** |
| --- | --- | --- |
| Implementation | Early indicators of the  uptake, integration and sustainability of evidence-based digital health interventions for a given context, including policies and practices. | What are the barriers and facilitators to sustainability?  What are the barriers and facilitators to the reach of the application?  What contextual factors affect the implementation of the application? |
| Feasibility and acceptability | Does the digital health system work as intended in a given context?  Can dementia carers use the app for the intended purpose? | Is it possible to develop an app that faithfully represents the needs of carers to support regularly physical activity?  Will carers be able to regularly use it?  Do carers want to use it? What are the motivations and barriers to using it?  Number of educational modules undertaken?  How many exercises/activities were started/completed?  How much time did participants spend using the app and on each component? |
| Usability | Can the digital health system be used as intended by users? | Was the app easy to use?  Would users learn how to perform basic tasks?  Was the interface use intuitive? |

**10.3 WS6 Planned intervention**

‘CareFit’ is a smartphone application (app) designed to support carers of people with dementia. It will be available on both Android and Apple smartphones. It has been co-designed with caregivers, health and social care professionals and physical activity experts. It was developed to support the early steps in physical activity behaviour change in line with the Transtheoretical model of behaviour change^^[[70]](#endnote-70)^^.

Carers would be expected to engage with the app in relatively short sessions of between 5 and 20 minutes at a time, to fit around their schedule. Activities (number and/or intensity/duration) would be expected to increase gradually over time, aligned to where an individual is within their stage within the transtheoretical model.

Like the ‘iSupport’ intervention, ‘CareFit’ has been designed for caregivers to decide how much or little they would like to do.

**10.3.1 Overview of the ‘CareFit’ app “tabs” with a description of their function**

| **Component** | **Function/detail** |
| --- | --- |
| **Activities** | Activity videos will be produced or reviewed by qualified fitness professionals including from the University of Strathclyde and at intensities set out within the UK Governments Physical Activity Guidelines for adults and older adults (e.g. NHS/UK Government physical activity guidelines). Videos will last approximately 5-minutes, or longer for warm-up and cool-down. Cardiovascular activities will be recorded in terms of time and intensity. Muscle and balance activities will be recorded in days, with the aim of reaching the recommended 2 days per week. Sedentary behaviour will be recorded by the number of sedentary breakers used each day/maximum time sedentary. These three main activity types align to the current UK physical activity guidelines. Participants will also have the option to record additional “active living” activities such as walking, climbing stairs or housework. |
| **Learn** | Educational materials (e.g. text, diagrams, video, audio) will guide users through the rationale and safe method of physical activity participation. Information will be provided in relation to physical activity (e.g. cardiovascular, muscle and balance and sedentary behaviour), decisional balance (e.g. pros and cons of physical activity), how to overcome the cons (e.g. barriers), physical activity goal setting, relapse prevention, and relaxation. Though users will be encouraged to start the learn section early in the study, this is optional for participants. |
| **Progress and planner tabs (including reminders)** | Participants will be asked to set their own goals for each week of the trial. The user will add activities to their weekly planner and when completed they will be recorded. If users choose to, there will be an option to add ‘activity’ calendar events into other personal online calendar software. Reminders can be set by users for activities/planners as desired. |
| **More** | Links to local resources and websites suitable for carers, localised to specific regional areas (e.g. Lanarkshire, Glasgow). |
| **Social/sharing functionalities** | Communication elements have been developed so that users can share their progress with family members and friends via a specific ‘share’ icon, which then links to social media applications and email accounts. Using the share function is entirely at the users’ own discretion.  Participants will also be invited to share with the research team their own experience with using the app to engage in physical activity. If participants specifically request it, we will have the capacity to share some limited information about transtheoretical model of behavioural change components (e.g. personal barriers and enablers and activities) within the app. For example, quotes can be presented within the app to highlight individual experiences, to celebrate/highlight progress and potentially motivate others. A separate ethical application and consent form at the University of Strathclyde covers the consenting details for this additional workflow. |

**10.4 WS6 research expected duration**

The total time scheduled for the WS6 feasibility study is 12 months. Key milestones will be monitored as part of overall project management.

**10.5 Selection and withdrawal of WS6 research participants**

WS6 will engage with health and social care professionals in an appropriate way relative to ongoing work pressures. Formal letters of collaboration will be sent to study collaborators (e.g. Carers Scotland, Alzheimer Scotland, H&S partnerships). We will ask collaborators to share study details with colleagues through emails, study adverts and other media.

Informal carers of people with dementia in the UK will be identified and recruited through our partner organisations’ networks (e.g. Carers UK, Alzheimer Scotland, Brain Health Scotland, private pharmacies, health and social care partnerships in Glasgow and Lanarkshire), the Join Dementia Research (JDR) database, and will be supported by other stakeholders through advertising via posters, emails, social media and centre referrals.

Staff from our partner organisations will also have the opportunity to consent into the online survey evaluation as CareFit is deployed (see section 10.11.1). Staff are not obliged to participate in the evaluation even where their organisation is a collaborator in this work.

Stakeholders working in the field of digital health, dementia or carer communities will be recruited using purposive sampling (see section 10.11.3 Stakeholder consultation to understand future use of the CareFit app). Stakeholders will be chosen from a variety of organisations that could directly relate to the implementation of CareFit, e.g. individuals working in the field of digital health, professionals from health and social care partnerships, charity organisations, local government, Public Health Scotland, and/or Scottish government, and NHS staff (although we will not advertise or promote WS6 in NHS organisations).

All participants are free to withdraw at any time without any impact on their future health and care or working role. Participant data collected to the point of withdrawal will be used in the analysis unless consent for this is specifically withdrawn.

**10.6 WS6 Inclusion criteria**

**Carers**

1. Adults (18+) living in Scotland who self-identify as an unpaid carer (partners, children, friends, etc.) of a person with dementia (self-reported)
2. Contemplating or preparing to undertake physical activity
3. Ability to undertake simple exercises such as arm raises or stretching
4. Be able to read and write in English
5. Have access to a smartphone (Android or Apple) alongside access to the internet
6. Normal or corrected to normal eyesight

**Health and Social Care Professionals**

1. Adults Aged 18 and over
2. Based in Scotland
3. Working as a health and social care professional
4. Willing to engage with the study (e.g. share information about ‘CareFit’ with carers of people with dementia) through their professional role for a period of at least 3 months.

**Stakeholders within digital health, dementia or carer communities**

1. Adults Aged 18 and over
2. Engaged or has expertise of interest in the digital health and/or dementia and/or carer community in Scotland

**10.7 WS6 Exclusion criteria**

**Carers**

1. Anyone advised by a clinician not to undertake physical activity or make any change in their present level of exercise
2. Are already regularly exercising to a significant level outside the home (e.g. running/cycling)
3. Residing outside Scotland at the time the study is conducted
4. Currently part of the main ‘iSupport’ study

**Health and Social Care Professionals**

1. Working exclusively within the National Health Service (NHS)

**Stakeholders within digital health, dementia or carer communities**

1. Not engaged with the dementia and/or carer community

**10.8 WS6 research consent procedure**

The method for taking consent will be technology mediated following the principles in the HRA and MHRA Joint statement on seeking consent by electronic methods^^[[71]](#endnote-71)^^.

Potential participants who contact the research team to register their interest in the study will be sent a copy of the Participant Information Sheet (PIS) and Consent Form. They will also be sent a link to an online Consent Form using an appropriate survey tool licensed by University of Strathclyde (e.g. Qualtrics). Participants will electronically select individual items in the online form, which correspond to the paper consent form, in order to confirm they have read and agree to each item in the consent form. Their electronic signature will be achieved by entering their first and surname, and then either typing or drawing their signature next to a declaration.

During the consent process potential participants can be supported by a researcher in an online meeting (e.g. Skype, Zoom) or telephone call, and if a potential participant has any questions they will be prompted to email the research team to arrange a call. They will then complete the online form, or the form will register that they declined.

Completed consent records will be kept by the University of Strathclyde separate to any data collected. The research team will remove consent information stored in Qualtrics once recruitment activity has concluded: data will then be stored on Strathclyde secure servers.

If WS6 participants are recruited in-person (e.g. while attending a project collaborator’s group), informed consent will be taken in-person in line with Good Clinical Practice (GCP) but stored electronically as described above.

**10.9 WS6 Randomisation and unblinding**

As this is a non-randomised feasibility study, randomisation and unblinding are not applicable.

**10.10 WS6 Withdrawal of participants**

Please see section 3.6 Withdrawal of participants.

**10.11 WS6 research procedures**

**10.11.1 Professional staff who support carers of people with dementia**

Professional staff who consent to take part will help advertise the study through their professional working role within Scotland.

To help professional staff learn about ‘CareFit’ we will develop a short video that will explain how the study works and what we are asking them to do. They will be contacted every 3 months to ask whether they are willing to continue working with us, or whether they would prefer to “exit” the study.

When exiting the study, we will share an “exit” questionnaire (see section 10.14 WS6 Data collection), which would take approximately 20 minutes to complete. We will send a maximum of two emails to ask for completion of their “exit” questionnaire. Where no response is received to emails, we will call professional staff to ask whether they would like to continue involvement or not. If there is no response, we will categorise the participant as lost to follow up and not follow up further. All professional staff involved will be asked to exit the study before the study end date.

Up to 6 professionals will be selected at random to take part in a one-to-one interview that will take approximately 30 minutes to complete.

**10.11.2 Carers of people with dementia**

Carers will be asked to download the ‘CareFit’ app for free on their personal Android or Apple smartphone/tablet from the Google Play or Apple stores. Participants would use the ‘CareFit’ app for a period of eight weeks. App usage data is anonymously collated by the research team to understand key aspects of use such as: implementation, acceptability, usability, and feasibility (see section 10.2 WS6 Research definitions). Participants will be asked to delete the ‘CareFit’ app from their smartphone/tablet at the end of this study, but given the opportunity to be notified when a public version is released.

**10.11.2.1 Before app use**

Participants who consent will be asked to complete a baseline questionnaire (see section 10.14 WS6 Data collection). If the participant requires support answering the questions, a researcher will arrange a video (e.g. Zoom, Teams or Skype) or telephone call.

Participants will be asked to complete a number of short education ‘learn’ sections while using the app.

**10.11.2.2 During app use**

Contacts will be made by email or automated within app ‘prompts’ where possible:

- At the 1 to 2-week mark after being sent the link to ‘CareFit’ to check in for any initial technical issues or support
- At the 4 to 5-week mark to see if they need any overall support using the application.

If a participant chooses to reply to the above, anonymised summary statistics will be collated, so that we can understand what types of supports would be required from participants in a definitive trial (e.g. number of requests, types of requests made).

**10.11.2.3 After app use**

Participants will be sent a questionnaire (as at baseline). In addition, participants will be asked questions related to their app experience, what components of the app are most important to them, and future intention to use the app including what they would keep, remove or change. If the participant requires support answering the questions, a researcher will arrange a video (e.g. Zoom, Teams or Skype) or telephone call.

Up to 15 participants will be selected at random for semi-structured, qualitative interviews to explore their experience of using the app.

**10.11.2.4 Post-WS6 Support**

All WS6 participants will be supported after the study through referral to relevant charities for further information. For example, links will be given at the conclusion of the follow up survey both to thank participants and link them directly to these external resources.

**10.11.3 Stakeholder consultation to understand future use of the CareFit app**

Near the end date of WS6, professionals who have previously been involved and individuals from organisations who have not previously been involved will be invited to contribute to a stakeholder consultation. Stakeholders will be invited to either a focus group (round table meeting), or a qualitative interview. Focus groups may be held in-person at the University of Strathclyde, or online using an internet-based platform (e.g. Zoom) and would take approximately 1 hour.

At the beginning of the focus group or interview, stakeholders will be given a short presentation introducing the CareFit app and some preliminary results of the study in order to provide context. The focus group or interview will be followed-up by a short Qualtrics survey designed to take approximately 20 minutes.

The focus group or interview topic guide was developed with unique questions that differentiate from the professional staff questionnaires (outlined in section 10.11.1). Such stakeholder consultation will help understand potential future use of CareFit in Scotland.

**10.12 WS6 Setting and context**

WS6 is led by University of Strathclyde and will be undertaken with adult dementia carers and health and social care professionals who live in Scotland.

**10.12.1 Safety considerations specific to WS6**

- Safety when undertaking exercises. Participants will be reminded when using the app that exercises must be undertaken in a safe space and must be proportional to physical ability. It remains better to do exercises carefully and slowly opposed to pushing too hard. Physical injuries can happen at any time, and participants will be encouraged to be aware of any new issues or problems that are worsening due to exercise and to seek help/advice wherever required. A H&S risk assessment has been conducted and stored on the University of Strathclyde risk assessment system (Number 7436 10/11/2022).
- Unexpected consequences/other risks of physical activity. Participants will be reminded that the tool is not a diagnostic app and that care as usual should continue. Participants will be encouraged to seek medical support as normal, should they have any specific health concerns. As with any exercise or physical activity, there is an inherent risk of injury or illness as a consequence. Participants will be required to consent that they do not have any medical reason that using such an app would not be advised for them, and regularly reminded during app use that they should stop using the app if there are any problems.
- Potential digital challenges/risks. We anticipate that a number of carers using our app may not have significant experience of using apps. Any application on a device is subject to security loopholes. We will ensure that reasonable efforts have been made to allow secure use of the app that will not compromise device security.
- Individuals who take part in our study will increase awareness about their health and may subsequently feel distressed that their own physical health is not where they would like it to be, or that they are significantly at risk of further decline in health.

Participants will be supported by the research team, including referring to partner organisations and/or health and social care professionals.

**10.13 WS6 Sampling and sample size**

Due to the preparatory nature of this study, no power calculation has been conducted. 50 carers will be recruited. A sub-sample of up to 15 carers will be randomly sampled for qualitative interviews following their participation in the feasibility study.

We aim to engage with up to 20 professionals who can comment on the use of ‘CareFit’ through questionnaires. A sub-sample of up to 6 professionals will be randomly sampled for qualitative interviews following their participation in this work.

For the separate focus group or qualitative interviews with stakeholders, we will employ purposive sampling methods (e.g. personal contacts at the University of Strathclyde or other organisations who have an interest in digital supports for carers) to recruit approximately 4 to 6 participants per session, and no more than 48 stakeholders in total.

**10.14 WS6 Data collection**

The primary mode of data collection for surveys will be technology mediated. Participants will be sent a personalised link to self-complete questionnaires online using an appropriate survey tool licenced by University of Strathclyde (e.g. Qualtrics).

Online questionnaires will be emailed to participants who are carers at baseline and 8-week follow-up. Demographic data will be collected at baseline only (e.g. age, gender, ethnicity, work status, local authority area, medical conditions). Behaviours and behavioural determinants will be measured in the baseline and follow-up questionnaire. Such measures will include:

- Physical activity & sedentary behaviour (for example, International physical activity questionnaire- short form^^[[72]](#endnote-72)^^, adapted question on breaks in sitting^^[[73]](#endnote-73)^^)
- Muscle Strength (for example, non-validated item adapted form strain et al.^^[[74]](#endnote-74)^^)
- Knowledge of Physical Activity (for example, non-validated items created for this study)
- Motivation (for example, non-validated items created with background information from Biddle et al.^^[[75]](#endnote-75)^^)
- Self-efficacy (for example, validated item from Marcus et al.^^[[76]](#endnote-76)^^)
- Social support (for example, non-validated item form Biddle et al.^75^)
- Health related quality of life (for example, EQ-5D-5L^53^)

Follow-up question items related to the usability, feasibility, implementation, effectiveness and maintenance of ‘CareFit’ will be added to the follow-up questionnaire for participants who are carers of people with dementia.

Health and social care staff will be sent a questionnaire at the end of their involvement in the study related to the reach, effectiveness, adoption, implementation, and maintenance of ‘CareFit’ in their organisation, and may be asked to share organisational documents and other materials if they wish.

All semi-structured qualitative interviews and focus groups will be audio recorded and transcribed. Draft topic guides have been developed using the RE-AIM framework^69^ and final topic guides will be adapted based on feedback from a testing phase of the application.

App usage data will be logged for analysis. A “log event” will be triggered each time the user enters or leave the app or an activity within the app. The logs will include a unique userID and deviceID and will be stored on AddJam (our development partner) servers. Logs will be securely transferred to the research team at the University of Strathclyde. AddJam will not hold any personal data.

A number of different measures based within the platform will be used to understand the user experience. These include data around:

Feasibility and acceptability: (i) The number, timing and duration of activity plans developed; (ii) The development of a personalised exercise plan for each user; (iii) The number of exercises planned, initiated and completed, use of the app across the different tab sections of “Activities”, “Progress”, “Learn”, “Planner” and “more”;

Usability: (i) Analysis of the user patterns of behaviour, including ‘bad’ patterns; (ii) Number of system crashes; (iii) Unresponsive gesture metrics, and (iv) in app feedback on each individual page. In addition, users will be able to vote on specific sections of the app in terms of whether they like or dislike during ongoing use.

The number of participants who contact the support email address and types of questions asked will be collated and anonymised.

**10.15 WS6 Data analysis**

Data will be used to provide insights and learnings from WS6 for future work aligned to the RE-AIM framework^69^. For example, data will be used to help identify mechanisms of impact of our intervention and hypothesis generation for future studies. All other statistical data will be summarised using n, Mean and Standard Deviation (SD), or by percentage frequency. For pre-post use questions, differences between groups will be identified using standard statistical techniques including paired t-tests^^[[77]](#endnote-77)^^, but as this is preparatory work, we are not measuring the significance of the intervention.

Qualitative interviews and focus groups will be transcribed verbatim. The six step approach of Braun and Clarkes thematic analysis^51^ will be used. The researcher will undertake a process of familiarising with the data, generating initial codes, development of initial themes, reviewing these themes, defining and naming the themes, and relating the analysis to the evaluation questions and previous literature^51^.

The data collected with carers, professionals and stakeholders will facilitate understanding of the potential for ‘CareFit’ to be implemented under real world conditions as a part of routine practices in the health and social care system. This will be done in line with the RE-AIM framework^69^. The results of this will inform further development of ‘CareFit’ and wider evaluations of this in a definitive trial.

Descriptive data, such as counting the number of times participants seek technical support, will be analysed and reported as part of the results to help us understand barriers for participants, including the ‘digital divide’. Additionally, outreach from participants to study team, screening rates, participation rates and dropout rates will be collected and reported to help analyse the reach of ‘CareFit’.

**11. Assessment of safety**

**11.1 Definitions**

Adverse Event (AE): Any untoward medical occurrence in either a trial or feasibility study participant which does not necessarily have a causal relationship with the intervention.

Serious Adverse Event (SAE): Any adverse event that a) Results in death; (b) Is life threatening; (c) Requires hospitalisation or prolongation of existing hospitalisation; (d) Results in persistent or significant disability or incapacity; or (e) Is otherwise considered medically significant by the investigator.

Pre-existing conditions do not qualify as adverse events unless they worsen over the course of the trial or feasibility study.

**11.2 Collecting, recording and reporting of adverse events**

Assessment of harm will be undertaken and overseen by an Independent Data Monitoring Committee (IDMC), who will report to the Trial Steering Committee (TSC). The IDMC will be able to advise on changes to the conduct of the trial via recommendations to the TSC and will also receive regular safety reports from the team.

The adverse event reporting period for the trial and feasibility study begins as soon as participants consent to take part and one month after their final data collection ends. All adverse event data will be collected and recorded in line with NWORTH’s SOP on Safety monitoring. Reporting of SAEs will also form part of the delegation log and be covered in training.

Reports will be sent to the Sponsor, the Research Ethics Committee, IDMC and TSC within the required timelines from the SOP. Other adverse events will be noted in the same log as the SAEs and a monthly report will be compiled by the Trial Manager.

Safety analysis will be pre-specified analyses in the statistical plan and can be represented graphically e.g. as volcano plots^^[[78]](#endnote-78)^^ or in the usual tabular format. The former plots all SAEs and provides a visual representation of outliers. This method is preferred to inferential analysis, as they would be under-powered. Using graphical methods will allow the IDMC to identify any potential safety signals and these will be reported to the TSC.

A copy of the AE and SAE CRF will be stored at the recruiting site in the ISF, and those signed by the Chief Investigator stored in the Trial Master File (TMF).

Given the nature of the intervention, we do not feel there are serious safety concerns for the person being cared for. However we will be collecting data on health and social care usage, and also the DEMQOL-proxy, which assesses the health-related quality of life of the care recipient. This data will be available to the IDMC during the course of the trial. In the reporting of unanticipated harms, we will include an assessment of whether the reported event could have an impact on the person being cared for.

**12. Project management**

The study is sponsored by Bangor University and the governance and management of the study will be undertaken by NWORTH. As a result, the study will adhere to NWORTH’s SOPs for all study and data management, statistical and regulatory matters. Study-specific SOPs will be developed as required and will be addressed throughout the study period and regularly reviewed. Best practice will be employed throughout to ensure both the trial and feasibility study are managed to the highest possible standard. Appropriate supervision and training of research staff and training in Good Clinical Practice (GCP) will be ensured. NWORTH’s Trial Manager will provide advice to the sites on all aspects of the running of the trial and feasibility study and will supply appropriate templates.

We have established a Trial Steering Committee (TSC), an Independent Data Monitoring Committee (IDMC) and a Trial Management Group (TMG). The TSC and IDMC will meet at agreed time intervals, which will be documented in the committees terms of reference or charter. Both will consist of an independent chair and an independent statistician. The IDMC will be able to advise on changes to the conduct of the trial and feasibility study via recommendations to the TSC and will also receive regular safety reports from the TMG.

**12.1 Trial Steering Committee**

The project’s TSC will oversee the running of the study on behalf of the sponsor and funder and will have overall responsibility for the continuation or termination of the trial and/or feasibility study. It will ensure that both the trial and feasibility study are conducted in accordance with the principles of GCP and the relevant regulations, and to provide advice on all aspects of the study.

**12.2 Independent Data Monitoring Committee**

The project’s IDMC will monitor the data and ethics aspects of the study and provide advice on changes to the conduct of the trial and feasibility study via recommendations to the TSC.

**12.3 Trial Management Group**

A TMG will oversee the day-to-day running of the trial and feasibility study and is composed of research team members, including the Chief Investigator, Trial Manager, Statistician etc. In addition, the group may include other members of the trial team with specific expertise, such as the Senior Software Engineer, Health Economists, and site Principal Investigators. The TMG will meet frequently during set up and subsequently on an agreed periodic basis once the trial is open to recruitment, and will monitor all aspects of conduct and progress, and ensure the protocol is adhered to.

**12.4 Patient and public involvement (PPI)**

Our PPI colleagues will be involved throughout the duration of the study and the CABAN group will formally meet twice a year in relation to ‘iSupport’. Masterson-Algar will support the CABAN group’s involvement with the trial and feasibility study. Co-applicant Hughes will join the TSC and contribute to the ongoing progress of the trial and feasibility study. She will jointly work with Masterson-Algar, assisting the development and facilitation of the feasibility study co-design workshops.

All public-facing documents will be finalised in consultation with our PPI group to ensure they are user-friendly and suitable for all levels of literacy skills. At the start of the project, all colleagues will contribute to the design of information sheets to help aid recruitment. After the intervention platform has been developed, our PPI group will help us make a short video to show other carers how to use ‘iSupport’. Some Welsh speaking members will help with pilot testing the Welsh version of ‘iSupport’ and research documents.

Throughout years 1 and 2, colleagues will help promote the trial and feasibility study to assist the recruitment of participants. They will discuss the development of interview questions for the process analysis. In year 3, they will advise on the interpretation of the research results and the production of a plain English/Cymraeg clir summary and assist with the dissemination of the study findings. The trial team will develop and deliver short and simple research methods sessions if required, e.g. ‘what is a randomised controlled trial?’ to help colleagues understand this specific research process.

The group will hold the project management team accountable for maintaining the DEEP-Ethics Gold Standards for Dementia Research.^^[[79]](#endnote-79)^^ The six principles include: Working in real partnership; respect and acknowledgement; safety and wellbeing; informed consent and capacity; confidentiality and anonymity; and information that is simple, accessible, and open.

Collaborators the Carers Trust will send relevant documentation and discuss specific issues with a virtual reference group, which meets monthly.

All PPI colleagues will be reimbursed for any travel expenses and will be thanked for their contributions with shopping vouchers. All payments will be recorded in line with the Monitoring Plan.

**12.5 Coronavirus (COVID-19) mitigation**

Due to the Coronavirus pandemic (COVID-19), we are following government guidelines regarding working from home. Remote contact will be privileged over in-person meetings (including for site initiation visits and training), where possible, to ensure that all staff are protected. Remote contact will be conducted using an internet-based service (e.g. Zoom, Teams or Skype), and through email communication.

Following any subsequent relaxing of the guidelines, if any in-person site visit need to be performed, these will only be undertaken where sites have been COVID-free for at least 14 days.

Both the staff conducting the site visit and all staff at the site will provide the answers to the following screening questions prior to the in-person visit to ensure risk is minimised:

- Have any staff been unwell recently (that could be attributable to COVID-19)?
- Have any staff had a recent onset of a new continuous cough?
- Have any staff had a high temperature? (temperatures may be checked and recorded).
- Have any staff noticed a loss or change in normal sense of taste or smell?
- Have any staff had recent contact (in the last 14 days) with anyone with COVID-19 symptoms or come into contact with someone who has been confirmed as COVID-19 positive?*

*If yes, they must follow the local rules and national regulations on self-isolation

Site visits will then be booked. During the visit all staff present will wear a mask, ensure social distancing and handwashing/hand sanitisation are performed, in line with local rules and national regulations. The provision and recording of details for all staff present will ensure ‘track and trace’ can be performed, should this be necessary. Approval from our respective institutions will be sought, and this procedure will only be implemented following approval.

**13. Ethics and regulatory approvals**

The study protocol, associated documentation, and all substantial amendments thereof will be submitted for review by Bangor University Schools of Health and Medical Sciences Research Ethics Committee (REC). The main ethical concern for both the trial and feasibility study are the process of gaining informed consent. The consent procedure is detailed in 3.3 Trial consent procedure and 6.3 Feasibility study consent procedure.

All researchers will have been fully trained in consent procedures and mental capacity. Applying the principles of the Mental Capacity Act (MCA), the researcher will support the potential participants to fully understand the nature of the trial and what is required of them. In line with the MCA Code of Practice this involves ascertaining the capacity to understand information, retain information and use or weight up the information to arrive at a decision/choice. This will be assessed by the researcher at each point of data collection at the point it needs to be made, i.e. when discussing the trial or feasibility study with a view to gaining consent, by going through the information sheet with them.

Good supervision will ensure a sensitive approach, and dilemmas will be discussed in regular meetings with the team, which include senior clinicians with many years of experience assessing capacity. If there is an indication of a lack of capacity, and a person is unable to give informed consent, the researcher will be trained how to manage that situation.

All information provided will be prepared in an acceptable manner that is clear and understandable. Bilingual information will be provided in Wales. The researchers will undertake the necessary checks (e.g. DBS) and be given full training and support in all procedures. In order to elicit data in a sensitive and appropriate manner and to ensure the questions are asked in a meaningful order, the interview schedule/questionnaires will first be piloted and revised as necessary. The research assistant in Wales will speak Welsh, should any participants prefer to undertake the study in Welsh.

**14. Monitoring**

**14.1 Quality Assurance (QA) and Quality Control (QC) of data**

QA includes all the planned and systematic actions established to ensure the trial and feasibility study are performed and data generated, documented/recorded and reported in compliance with the principles of GCP and applicable regulatory requirements.

QC is the operational techniques and activities undertaken within the quality assurance system to verify that the requirements for quality of the research-related activities are fulfilled.

**14.2 Risk assessment**

A risk assessment has been conducted by a cross functional team in order to identify the potential risks/hazards associated with the trial and inform the appropriate approach to monitoring. It was also used in the composition of this protocol.

**14.3 Monitoring plan**

A Monitoring Plan will be prepared prior to participant recruitment detailing the monitoring strategy for the trial and feasibility study. The plan will include requirements for day-to-day centralised monitoring, and any requirements identified in the risk assessment.

**14.4 Source data**

The CRF will be considered the source data and should be consistent and verifiable with the information recorded in MACRO. Information regarding how the data is to be collected, stored, and transferred is included in the Data Management Plan which will be stored in the TMF.

**14.5 Direct access to source data and documents**

In order to perform their role effectively, monitors and persons involved in QA and inspection may need direct access to source data. Since this affects the participant’s confidentiality, this fact will be included on the Patient Information Sheet and Informed Consent Form.

**14.6 Confidentiality**

All data will be handled in accordance with General Data Protection Regulation (2018).

The CRFs will not include the participant’s name or other personal identifiable data. Audio recordings containing personal identifiers will be substituted for pseudo names during transcription.

All trial staff and members of the research team will preserve the confidentiality of participants taking part in the trial or feasibility study, and the Sponsor is registered as a Data Controller with the Information Commissioners Office.

All interviews will be conducted in a way to ensure privacy and confidentiality (e.g. there is no-one else in the room with the researcher at the time) and all data will be securely stored in lockable areas.

**15. Data handling**

All aspects of the trial and feasibility study will be managed in accordance with General Data Protection Regulations (GDPR), principles of GCP, and relevant NWORTH SOPs.

In light of the current COVID-19 pandemic, all members of the research team are currently working from home. The security of confidential data will be upheld in line with governing policies of the research team’s respective institutions. This will ensure that all data is securely stored electronically using password protected computers and in lockable areas in the home, or if working in offices in a locked filing cabinet.

Participants will be allocated a unique study number, which will be used in any documentation associated with the trial or feasibility study. Participants’ names will not appear on any documentation associated with the trial or feasibility study.

A Data Management Plan will be developed to outline the responsibilities of all staff and the procedures for collecting, handling, and transferring data. This will be developed in line with NWORTH SOPs.

**16. Pathways to impact**

The Chief Investigator and all co-applicants will prepare and agree a publication policy, which will be reviewed by the TMG, to agree on authorship of future papers and other outputs from the study. Multiple routes will be taken to the dissemination of the study:

A dedicated ‘iSupport’ project webpage will be developed. All participants will receive up to 4 study updates during the project timescale. The ‘iSupport’ intervention platform and an adapted version of ‘iSupport’ for young carers will be widely available across the UK and globally at the end of the research. We will produce data on the recruitment, retention, data quality and acceptability of ‘iSupport’ for young carers to inform a larger definitive study, along with exploring a range of appropriate outcome measures for this group. This could give an indication of the likely magnitude of possible movement within an outcome measure and some suggestion of variability, which could inform a future sample size calculation, along with further evidence from the literature.

We will develop a ‘how to use ‘iSupport’’ video to help other carers who may lack confidence with using technology. We will produce at least 5 academic papers that will be published open access to ensure maximum use, and an article for a practitioner magazine (e.g. Journal of Dementia Care). We will present the findings at academic conferences (e.g. Alzheimer’s Europe; Alzheimer’s Disease International). We will work with our stakeholders and PPI group to deliver up to three stakeholder and public events, and to produce plain English/Cymraeg clir summaries of the research findings that are visually appealing. Findings will be presented at the Carers Trust annual conference. We will develop policy briefings for our respective devolved governments. A short video developed with young carers will capture some of the main aspects of the adaptation (including methodological guidance) which will be of use to others who may also want to adapt ‘iSupport’ for young carers in other countries. Carers Trust will disseminate information via their magazine to over 150 carers services in the UK.

**17. Indemnity**

Cover for harm as a result of the design or conduct of the study has been arranged with the study Sponsor.

**18. Financial aspects**

This study is funded by the National Institute for Health Research (NIHR) Public Health Research and will be managed in accordance with the relevant policies and procedures.

**19. Definition of end of study**

This is defined as the date of the last assessment of the last participant.

**20. Archiving**

Archiving will be conducted in line with NWORTH’s SOP on Archiving. The Data Management Plan will also describe the requirements for data archiving, and responsibilities will be documented in the Delegation Log.

**21. Research expertise**

**GW** is a Professor of Ageing and Dementia Research and Associate Director of the Wales Centre for Ageing and Dementia Research, with extensive expertise in the leadership of research studies. GW will lead the trial.

**RTE** is a Professor of Health Economics and Co-Director of Health and Care Economics Cymru (formerly WHESS). She has extensive experience of research involving people living with dementia and their carers, and will lead WS3.

**PMA** is an experienced researcher within the field of neurological conditions and their impact on individuals and their families. Her recently completed research fellowship explored, applying innovative co-design approaches, the experiences of young people living in families affected by neurological conditions such as dementia. PMA will lead the feasibility study and process evaluation.

**JS** is an Associate Professor of clinical psychology at UCL and clinical psychologist with a track record in leading dementia research projects including development of clinical interventions for people affected by dementia and evaluating online support for carers. He will lead the English arm of the trial. He is also clinical director of the largest clinical psychology training course in the country (150 active students at any one time).

**AS** is a Professor of Old Age Clinical Psychology. She has extensive experience in the development and evaluation of psychosocial interventions, including Cognitive Stimulation Therapy (CST), which is recommended by UK government guidelines and is the primary psychosocial intervention offered by UK memory clinics.

**KE** is an experienced researcher and core member of the Digital Health and Wellness team at the University of Strathclyde. He gained prominent international experience within the World Health Organization developing ‘iSupport’, and has worked on a number of technology-based studies including Randomised Controlled Trials in Dementia. Since moving to Strathclyde in early 2018 he has established strong working links with Alzheimer Scotland and clinical NHS colleagues making an ideal recruitment base within the Scottish setting. He will lead the Scottish arm of the trial.

**ZH**, as principal statistician of NWORTH CTU, will provide statistical and methodological oversight for the project and will supervise all statistical analysis.

**GH** is a young carer of a family member with dementia. She is a member of the TSC and is the research assistant for the Welsh arm of the trial, where she has assisted in adapting ‘iSupport’ for Welsh language speakers and will undertake bilingual assessments. .

**GF,** as Trial Manager at NWORTH, will co-ordinate all aspects of quality management and regulatory issues, and will provide advice to the team on all aspects of the running of the trial.

**RI and SK** are research assistants working on the Scottish and English arms of the trial, and all have prior experience of conducting quantitative and qualitative research. All research assistants will be fully trained in all the trial procedures.

**BA** is an experienced health economist with expertise in designing and conducting cost-effectiveness analyses in both health and social care research, and will work on WS3 under the supervision of RTE.

**EF, AB and BA** are experienced research assistants on WS5 working from UCL.

**RM, MD, BF and AK** are experienced Co-Investigators on WS6 and will support the aims of this work-stream through their expertise and networks.

**RM and WH** are experienced research assistants on WS6 working from the University of Strathclyde.

**22. Research collaborators**

**Carers Trust** will promote the programme, support recruitment and help ensure the programme is designed to be equitable, accessible and effective, based on in-depth knowledge of working with unpaid carers from across the UK.

**Alzheimer Scotland** are a leading and innovative dementia charity with over 9,000 members, 90,000 dementia friends and support from 1,000 volunteers. The organisation currently has 21 Dementia Resource Centres (DRCs) spread geographically right across Scotland, regularly supporting individuals with a wide variety of services including obtaining information, training and peer support. Their localised efforts, health and social care links and involvement in developing National Dementia Strategies for Scotland make them an ideal partner for this ‘iSupport’ study across platform development, recruitment and ensuring impact and implementation.

**Professor Anne Margriet Pot** is strategic advisor Care for Older People at the Health Care Inspectorate, Ministry of Health, the Netherlands. From 2014 till 2018, she was posted in Geneva at the World Health Organisation (WHO), where was responsible for the development of ‘iSupport’: WHO’s extensive online training and support program for carers of people with dementia. Anne Margriet Pot is also endowed professor at the Vrije Universiteit Amsterdam, extraordinary professor at Optentia, North West University, Johannesburg, South Africa and honorary professor at the University of Queensland, Australia.

We are working closely with the World Health Organisation who are providing expertise and input regarding ‘iSupport’. We are also working closely with the Pan-American Health Organisation who are developing the ‘iSupport’ online platform for our trial and feasibility study.

**23. Protocol amendments**

**23.1 Current version of the protocol**

**Version 5 dated 06/09/2023.**

**23.2 Amendments**

| **Pg/section** | **Changes to protocol since v4** |
| --- | --- |
| Pg.1 | Protocol version and date updated. |
| Pg.2 | Planned End Date updated to accommodate 6-month non-costed extension, agreed by NIHR project manager 16/08/2023 |
| Contents | Minor changes to page number to include new interviews in sections 9 and 10, as outlined in this table. |
| 2.3 | Minor change to update total time scheduled from 36 to 42 months due to 6-month non-costed extension, and change to remove bold font formatting. |
| 4.5.6 | Minor change to remove sentence on CACE analysis which is no longer needed. |
| 9.2 | Addition of a third research question to take into account additional semi-structured interviews and/or focus groups with professionals (hence “work with professionals”). |
| 9.4 | Addition of new paragraph to include participants selected for work with professionals. |
| 9.10 | Addition of new paragraph to include procedure for work with professionals. |
| 9.13 | Addition of new paragraph to include sample size for work with professionals. |
| 9.14 | Minor change to make clear 15 participants in the existing qualitative interviews will be selected from the feasibility study, and more information on the topics for these interviews;  Addition of new paragraph to include data collection for work with professionals. |
| 9.15 | Addition of new paragraph to include data analysis for work with professionals. |
| 10. | Minor changes throughout section to standardise word ‘CareFit’. |
| 10.5 | Addition of new paragraph to include participants selected for semi-structured interviews and/or focus groups with stakeholders (hence “work with stakeholders”). |
| 10.6 | Addition of new inclusion criteria for work with stakeholders. |
| 10.7 | Addition of new exclusion criteria for work with stakeholders. |
| 10.11.3 | New sub-section to include procedures for work with stakeholders. |
| 10.13 | Addition of new paragraph to include sample size for work with stakeholders. |
| 10.14 | Minor change to add focus groups will collect data the same as semi-structured qualitative interviews. |
| 10.14 | Minor change to add focus groups will analyse data the same as semi-structured qualitative interviews;  Minor change to add data from stakeholders will be analysed the same as for professionals. |
| 21 | Minor change to remove research assistants JC and FAI, who have left the study;  Minor change to add WS6 research assistant RM and remove BM. |
| 23.1 | Minor change to update protocol version and date. |

| **Pg/section** | **Changes to protocol since v3** |
| --- | --- |
| Pg.1 | Protocol version and date updated. |
| Contents | Minor changes to include addition of new section 9 and subsequent amendments for subsequent section numbers. |
| 3.3 | Minor changes to consent flowchart: sources of information for screening participants made more clear; consent records will be stored in University computer folders with access restrictions; Participant IDs and consent status will be saved in a spreadsheet; Information about support services offered to potential participants who are not eligible.;  Minor change to wording to make more clear where paper and electronic consent records are stored. |
| 4.6.1 | Minor change to make clear email link is to questions about using iSupport. |
| 5. | Minor change to add “WS4” in the section title, as this is what the Feasibility study is referred to in study documents. |
| 5.1 | Minor change to add “WS4” in the section title, and “(WS4)” in the first sentence. |
| 5.3 to 7.3 | Minor changes to add “WS4” in the section titles for: 5.3; 5.4; 6.; 6.3; 7.; and 7.3. |
| 7.3.2 | Minor change to make clear WS4 Feasibility study Phase 2 will use some new outcome measures for young people which were not used in WS1;  Addition of new paragraph to include semi-structured interviews and/or focus groups with parents and professionals. |
| 7.3.3 | Addition of new sentence to include semi-structured interviews and/or focus groups with parents and professionals. |
| 7.3.4 | Minor change to include how parents will be recruited for the semi-structured interviews and/or focus groups. |
| 7.3.5 | Minor change to clarify online evaluation can be asked before participants complete T2 data collection.  Addition of new paragraph to include semi-structured interviews and/or focus groups with parents and professionals. |
| 7.3.6 | Minor change to update reference from 59 to 64;  Addition of new paragraph to include semi-structured interviews and/or focus groups with parents and professionals. |
| 9. | New section and sub-sections to include work-stream 5 (WS5), an additional project to feasibility test the Bengali adaptation of iSupport. WS5 will follow procedures laid out in this new section and in other sections of the ‘iSupport’ protocol (as referenced). WS5 is being led by co-applicant Aimee Spector from UCL. WS5 is funded by the NIHR in a variation to contract to the main ‘iSupport’ grant (NIHR_130914). |
| 10. | New section and sub-sections to include work-stream 6 (WS6), an additional project to explore recruitment feasibility and adaptation of a physical activity application ‘CareFit’ for dementia carers. WS6 will follow procedures laid out in this new section and in other sections of the ‘iSupport’ protocol (as referenced). WS6 is being led by co-applicant Kieren Egan from University of Strathclyde. WS6 is funded by the NIHR in a variation to contract to the main ‘iSupport’ grant (NIHR_130914). |
| 11. to 23. | Amendment to section numbers (including all sub-section numbers) to accommodate two new sections as above. Affected sections: 11; 12; 13; 14; 15; 16; 17; 18; 19; 20; 21; 22; and 23. |
| 21 | Minor change to add WS5 research assistants EF, AB and BA;  Minor change to add WS6 co-investigators RM, MD, BF and AK;  Minor change to add WS6 research assistants BM and WH. |
| 23.1 | Minor change to update protocol version and date. |
| 24. | Amendment from section number 21 to 24. |
| 25 | Amendment from section number 22 to 25;  New references 66-77, added for new section 10 as above. |
| Footer | Protocol version and date updated. |

| **Pg/section** | **Changes to protocol since v2** |
| --- | --- |
| Pg.1 | Protocol version and date updated;  New sentence to add “IRAS ID: 311565”;  Sponsor Representative name and email changed. Previous representative has left Bangor University. |
| Pg.2 | Co-investigator Paul Brocklehurst removed. He has taken up a new post elsewhere and is unable to continue with the study. |
| Contents | Minor changes to include addition of new section 17 and subsequent amendments for subsequent section numbers. |
| 10.4 | Minor change to change responsibility from Algar-Skaife to Masterson-Algar. |
| 17. | New section to include Definition of end of study. |
| 18. | Amendment from section number 17 to 18. |
| 19. | Amendment from section number 18 to 19;  Minor change from “FI” to “RI, FAI”;  Minor change from “DP” to “SK”;  Minor change to remove PB who has left the study, and amend the health economist to include initials for BA. |
| 20 | Amendment from section number 19 to 20. |
| 21 | Amendment from section number 20 to 21. |
| 21.1 | Minor change to update protocol version and date. |
| Footer | Protocol version and date updated. |

| **Pg/section** | **Changes to protocol since v1** |
| --- | --- |
| Pg.1 | Protocol version and date updated; |
| Pg.2 | Co-investigator Kat Algar-Skaife removed. She has taken up a new post elsewhere and is unable to continue with the study. Patricia Masterson-Algar is now leading the process evaluation (changes agreed by the NIHR).  Contact email for GH updated. |
| 2.1 | Minor change to bullet points order to match section 2.2. |
| 4.3.1 | New section to describe research sites. |
| 4.3.2 | New section for organogram of research sites and study reporting. |
| 4.5 | Minor change to remove “competence” (see section 4.5.4 below). |
| 4.5.1 | Minor changes to make clear individual criteria to be considered in the context of the whole study, as recommended by the IDMC and recorded in meeting minutes from 25/05/2021. |
| 4.5.4 | Minor change to remove information about the Short Sense of Competence Questionnaire (SSCQ), as it was agreed with NIHR to remove from the case report form (CRF) due to overlap with other measures. |
| 4.7 | Minor changes to remove reference to some health economics questions which were removed from the CRF, following initial piloting on the CRF. |
| 4.7.1 | Minor changes to remove reference to some health economics questions which were removed from the CRF, following initial piloting. |
| 11. | Minor change to make clear the research ethics committee. |
| 18. | Minor changes to content for PMA, JS, GH, and “FI, JC and DP”.  Minor changes to remove 2 researchers previously listed who have left the study, and add a sentence to capture health economist post. |
| 19. | Minor change to remove reference to Faaiza Bashir, as she has left the Carers Trust (who are still named collaborators). |
| 20.1 | Minor change to update protocol version and date. |
| 22. | References removed for SSCQ, as above for section 4.5.4. |
| Footer | Protocol version and date updated. |

**24. Appendices**

[Appendix 1: ‘iSupport’ logic model 59](#_Toc144913578)

*Appendix 1: ‘iSupport’ logic model*


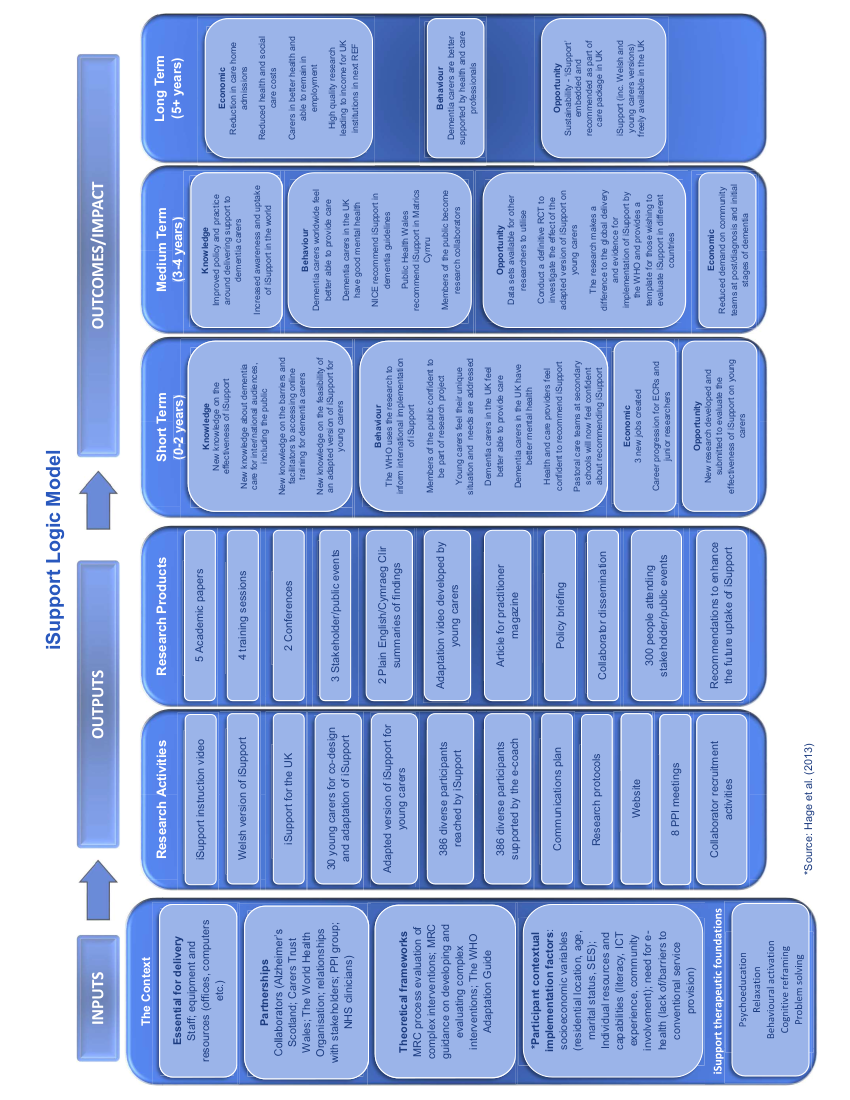


**25. References**

1. Lewis, et al., *Trajectory of Dementia in the UK – Making a Difference*. Office of Health Economics for Alzheimer’s Research UK 2014. Available from: https://www.alzheimersresearchuk.org/wp-content/uploads/2015/01/OHE-report-Full.pdf [↑](#endnote-ref-1)
2. Pinquart M, Sörensen S. Differences between carers and non-carers in psychological health and physical health: A meta-analysis. *Psychology and Aging*. 2003;18(2):250–67. [↑](#endnote-ref-2)
3. Gilhooly KJ, Gilhooly MLM, Sullivan MP, et al. A meta-review of stress, coping and interventions in dementia and dementia caregiving. *BMC Geriatrics*. 2016;16(106). Available from: doi:10.1186/s12877-016-0280-8. [↑](#endnote-ref-3)
4. Alzheimer’s Society. *Dementia UK. Update*. 2014. Available from: https://www.alzheimers.org.uk/sites/default/files/migrate/downloads/dementia_uk_update.pdf [↑](#endnote-ref-4)
5. World Health Organisation. *Global action plan on the public health response to dementia 2017–2025*. 2017. Available from: https://apps.who.int/iris/bitstream/handle/10665/259615/9789241513487-eng.pdf;sequence=1 [↑](#endnote-ref-5)
6. Welsh Government. *Dementia Action Plan for Wales, 2018-2022*. 2018. Available from: https://gov.wales/sites/default/files/publications/2019-04/dementia-action-plan-for-wales.pdf [↑](#endnote-ref-6)
7. Scottish Government. *National Dementia Strategy, 2017-2020*. 2017. Available from: https://www.gov.scot/publications/scotlands-national-dementia-strategy-2017-2020/ [↑](#endnote-ref-7)
8. Department of Health. *Living well with dementia: A national dementia strategy*. 2009. Available from: https://www.gov.uk/government/publications/living-well-with-dementia-a-national-dementia-strategy [↑](#endnote-ref-8)
9. National Institute of Health and Care Excellence (NICE). *Dementia: assessment, management and support for people living with dementia and their carers*. 2018. Available from: https://www.nice.org.uk/guidance/ng97 [↑](#endnote-ref-9)
10. McCabe M, You E, Tatangelo G. Hearing Their Voice: A Systematic Review of Dementia Family Carers’ Needs. *The Gerontologist*. 2016;56(5): 70–88. Available from: https://doi.org/10.1093/geront/gnw078. [↑](#endnote-ref-10)
11. Dickinson C, et al. Psychosocial intervention for carers of people with dementia: What components are most effective and when? A systematic review of systematic reviews. *International Psychogeriatrics*. 2017;29(1): 31-43. [↑](#endnote-ref-11)
12. Mehta KM, Gallagher-Thompson D, Varghese M, et al. ‘iSupport’, an online training and support program for carers of people with dementia: study protocol for a randomized controlled trial in India. *Trials*. 2018;19(1): 271. Available from: DOI: 10.1186/s13063-018-2604-9. [↑](#endnote-ref-12)
13. Pinto-Bruno ÁC, Pot AM, Kleiboer A, Droes RM, van Straten A. An Online Minimally Guided Intervention to Support Family and Other Unpaid Carers of People With Dementia: Protocol for a Randomized Controlled Trial. *JMIR Research Protocols*. 2019;8(10):e14106. Published 2019 Oct 10. Available from: doi:10.2196/14106. [↑](#endnote-ref-13)
14. Teles S, Ferreira A, Seeher K, et al. Online training and support program (‘iSupport’) for informal dementia carers: protocol for an intervention study in Portugal. *BMC Geriatrics*. 2020;20(10). Available from: https://doi.org/10.1186/s12877-019-1364-z [↑](#endnote-ref-14)
15. Moore GF, Audrey S, Barker M, Bond L, Bonell C, Hardeman W, et al. Process evaluation of complex interventions: Medical Research Council guidance. *BMJ*. 2015;350:h1258. [↑](#endnote-ref-15)
16. O'Cathain A, Croot L, Duncan E, et al. Guidance on how to develop complex interventions to improve health and healthcare. *BMJ Open*. 2019;9:e029954. Available from: doi: 10.1136/bmjopen-2019-029954. [↑](#endnote-ref-16)
17. Pfadenhauer LM, Gerhardus A, Mozygemba K, et al. Making sense of complexity in context and implementation: the Context and Implementation of Complex Interventions (CICI) framework. *Implementation Science*. 2017;12(21). ISSN 1748-5908. [↑](#endnote-ref-17)
18. Bédard M, Molloy DW, Squire L, Dubois S, Lever JA, O'Donnell M. The Zarit Burden Interview: a new short version and screening version. *Gerontologist*. 2001;41(5):652-657. [↑](#endnote-ref-18)
19. Radloff LS. CES-D scale: a self report depressions scale for research in the general populations. *Applied Psychological Measurement*. 1977;1(3): 385–401. [↑](#endnote-ref-19)
20. Andresen EM, Byers K, Friary J, Kosloski K, Montgomery R. Performance of the 10-item Center for Epidemiologic Studies Depression scale for caregiving research. *SAGE Open Medicine*. 2013. Available from: https://doi.org/10.1177/2050312113514576. [↑](#endnote-ref-20)
21. Join Dementia Research is funded by the Department of Health and delivered by the National Institute for Health Research in partnership with Alzheimer Scotland, Alzheimer's Research UK and Alzheimer's Society - www.joindementiaresearch.nihr.ac.uk [accessed 10/03/2021]. [↑](#endnote-ref-21)
22. Russell I, Hoare ZS, et al. Generalised method for adaptive randomisation in clinical trials. *Statistics in Medicine*. 2011;30(9): 922–934. [↑](#endnote-ref-22)
23. Sörensen S, Pinquart M, Duberstein, P. How effective are interventions with carers? An updated meta-analysis. *Gerontologist*. 2002;42(3): 356-72. [↑](#endnote-ref-23)
24. Kitwood, T. *Dementia reconsidered: The person comes first*. England: Open University Press; 1997. [↑](#endnote-ref-24)
25. Pot AM, Gallagher-Thompson D, Xiao LD, Willemse BM, Rosier I, Mehta KM, et al. ‘iSupport’: a WHO global online intervention for informal carers of people with dementia. *World Psychiatry*. 2019. [↑](#endnote-ref-25)
26. Office for National Statistics. *2011 Census: key statistics for Wales, March 2011*. 2011. Available from: https://www.ons.gov.uk/peoplepopulationandcommunity/populationandmigration/populationestimates/bulletins/2011censuskeystatisticsforwales/2012-12-11#proficiency-in-welsh [↑](#endnote-ref-26)
27. Welsh Government. *More than just words*. 2015. Available from: http://www.wales.nhs.uk/sites3/Documents/415/A%20active%20offer%20information%20pack%20-%20Health%20-%20FINAL1.pdf [↑](#endnote-ref-27)
28. Alzheimer’s Society. *Caring for a person with dementia: A practical guide*. Available from: https://www.alzheimers.org.uk/get-support/publications-factsheets/caring-person-dementia-practical-guide [↑](#endnote-ref-28)
29. Higginson IJ, Gao W, Jackson D, Murray J, Harding R. Short-form Zarit Carer Burden Interviews were valid in advanced conditions. *Journal of Clinical Epidemiology*. 2010;63(5): 535–542. Available from: doi: 10.1016/j.jclinepi.2009.06.014. [↑](#endnote-ref-29)
30. Leng M, Zhao Y, Xiao H, Li C, Wang Z. Internet-Based Supportive Interventions for Family Carers of People With Dementia: Systematic Review and Meta-Analysis. *J Med Internet Res*. 2020 Sep 9;22(9):e19468. doi: 10.2196/19468. PMID: 32902388; PMCID: PMC7511858. [↑](#endnote-ref-30)
31. Ying J, Yap P, Gandhi M, Liew T, M: Validity and Utility of the Center for Epidemiological Studies Depression Scale for Detecting Depression in Family Carers of Persons with Dementia. *Dement Geriatr Cogn Disord* 2019;47:323-334. doi: 10.1159/000500940 [↑](#endnote-ref-31)
32. Sugimoto, T. and Sozu, T. and Hamasaki, T. (2012). A convenient formula for sample size calculations in clinical trials with multiple co-primary continuous endpoints. *Pharmaceut. Statist*.,11:118-128. doi:10.1002/pst.505 [↑](#endnote-ref-32)
33. Sozu, T. and Sugimoto, T. and Hamasaki, T. and Evans, S.R. (2015).*Sample Size Determination in Clinical Trials with Multiple Endpoints*. Springer Briefs in Statistics, ISBN 978-3-319-22005-5. [↑](#endnote-ref-33)
34. Vasileiou K, Barnett J, Thorpe S, et al. Characterising and justifying sample size sufficiency in interview-based studies: systematic analysis of qualitative health research over a 15-year period. *BMC Medical Research Methodology*. 2018;18(148). Available from: https://doi.org/10.1186/s12874-018-0594-7. [↑](#endnote-ref-34)
35. Ritchie J, Lewis J, Elam G. Designing and selecting samples. In: Ritchie J, Lewis J. (eds.) *Qualitative research practice: a guide for social science students and researchers*. London: Sage; 2003. p.77–108. [↑](#endnote-ref-35)
36. Deeken, F., Rezo, A., Hinz, M., Discher, R., & Rapp, M. A. (2019). Evaluation of Technology-Based Interventions for Informal Carers of Patients With Dementia-A Meta-Analysis of Randomized Controlled Trials. *The American journal of geriatric psychiatry : official journal of the American Association for Geriatric Psychiatry*, 27(4), 426–445. https://doi.org/10.1016/j.jagp.2018.12.003 [↑](#endnote-ref-36)
37. O’Rourke N, Tuokko HA. Psychometric Properties of an Abridged Version of the Zarit Burden Interview Within a Representative Canadian Carer Sample. *The Gerontologist*. 2003;43(1):121-127. [↑](#endnote-ref-37)
38. Gratão ACM, Brigola AG, Ottaviani AC, Luchesi BM, Souza EN, Rossetti ES, et al. Brief version of Zarit Burden Interview (ZBI) for burden assessment in older carers. *Dementia e Neuropsychologia*. 2019;13(1): 122–129. Available from: doi: 10.1590/1980-57642018dn13-010015. [↑](#endnote-ref-38)
39. Ying J, Yap P, Gandhi M, Liewa TM. Validity and Utility of the Center for Epidemiological Studies Depression Scale for Detecting Depression in Family Carers of Persons with Dementia. *Dementia and Geriatric Cognitive Disorders*. 2019;47(4-6): 323–334. Available from: doi: 10.1159/000500940. [↑](#endnote-ref-39)
40. Spitzer R, Kroenke K, Williams JBW, Lowe B. A brief measure for assessing generalised anxiety disorder. *Archives of Internal Medicine*. 2006;166(10):1092-1097. [↑](#endnote-ref-40)
41. McKechnie V, Barker C, Stott J. The Effectiveness of an Internet Support Forum for Carers of People With Dementia: A Pre-Post Cohort Study. *Journal of Medical Internet Research*. 2014;16(2):e68. [↑](#endnote-ref-41)
42. National Collaborating Centre for Mental Health. *The Improving Access to Psychological Therapies Manual*. 2019. Available from: https://www.england.nhs.uk/wp-content/uploads/2020/05/iapt-manual-v4.pdf. [↑](#endnote-ref-42)
43. Mountain G, Moniz-Cook ED, Ǿksenberg L. *Dementia Outcome Measures: Charting New Territory.* (Report of a Joint Programme for Neurodegenerative Diseases Working Group on Longitudinal cohorts). 2015. Available from: https://www.neurodegenerationresearch.eu/wp-content/uploads/2015/10/JPND-Report-Fountain.pdf [↑](#endnote-ref-43)
44. Wagnild GM. *The Resilience Scale User’s Guide for the US English version of the Resilience Scale and the 14-item Resilience Scale (RS-14)*. The Resilience Center, Montana. 2009. [↑](#endnote-ref-44)
45. D’Onofrio G, Sancarlo D, Raciti M, Burke M, Teare A, Kovacic T et al. MARIO Project: Validation and Evidence of Service Robots for older people with dementia. *Journal of Alzheimer’s Disease*. 2019;68(4): 1587-1601. [↑](#endnote-ref-45)
46. Spruytte N, Van Audenhove C, Lammertyn F. Internal Report: *The Scale for the Quality of the Current Relationship*. Leuven: LUCAS-KULeuven. 2000. [↑](#endnote-ref-46)
47. Annear MJ, Toye C, Elliott KJ, McInerney F, Eccleston C, Robinson, A. Dementia knowledge assessment scale (DKAS): confirmatory factor analysis and comparative subscale scores among an international cohort. *BMC Geriatrics*. 2017;17(168). Available from: doi:10.1186/s12877-017-0552-y. [↑](#endnote-ref-47)
48. Mulhern, B., Rowen, D., Brazier, J., Smith, S., Romeo, R., Tait, R., Watchurst, C., Chua, K. C., Loftus, V., Young, T., Lamping, D., Knapp, M., Howard, R., & Banerjee, S. (2013). Development of DEMQOL-U and DEMQOL-PROXY-U: generation of preference-based indices from DEMQOL and DEMQOL-PROXY for use in economic evaluation. *Health technology assessment (Winchester, England), 17(5)*, v–140. https://doi.org/10.3310/hta17050 [↑](#endnote-ref-48)
49. Brooke J. SUS: A ‘quick and dirty’ usability scale. In: Jordan PW, Thomas B, Weerdmeester BA, McClelland IL. (eds.) *Usability Evaluation in Industry*. London, UK: Taylor Francis; 1996. p.189–194. [↑](#endnote-ref-49)
50. May C, Finch T, Mair F, Ballini L, Dowrick C, Eccles M, et al. Understanding the implementation of complex interventions in health care: the normalization process model. *BMC Health Services Research*. 2007;7:148. DOI: 10.1186/1472-6963-7-148. [↑](#endnote-ref-50)
51. Braun V, Clarke V. Using thematic analysis in psychology. *Qualitative Research in Psychology*. 2006;3(2): 77-101. ISSN 1478-0887. [↑](#endnote-ref-51)
52. National Institute for Health and Care Excellence (NICE). *Methods for the development of NICE public health guidance (third edition)*. 2012. Available from: https://www.nice.org.uk/process/pmg4/chapter/introduction. [↑](#endnote-ref-52)
53. Herdman M, Gudex C, Lloyd A, Janssen MF, Kind P, Parkin D, et al. Development and preliminary testing of the new five-level version of EQ-5D (EQ-5D-5L). *Quality of life research*. 2011;20(10): 1727-1736. [↑](#endnote-ref-53)
54. Aguirre E, Kang S, Hoare Z, Edwards RT, Orrell M. How does the EQ-5D perform when measuring quality of life in dementia against two other dementia-specific outcome measures. *Quality of Life Research*. 2016;25(1): 45-49. [↑](#endnote-ref-54)
55. Curtis L, Burns A. *Unit costs of health and social care 2019*. Canterbury: Personal Social Services Research Unit, University of Kent; 2019. [↑](#endnote-ref-55)
56. NHS Improvement. *National Cost Collection for the NHS*. https://improvement.nhs.uk/resources/national-cost-collection/ [Last accessed 10th March 2020]. [↑](#endnote-ref-56)
57. Hoefman RJ, Van Exel NJA, Brouwer WBF. *iMTA Valuation of Informal Care Questionnaire (iVICQ)*. Version 1.0 (December 2011). Rotterdam: iBMG / iMTA, 2011. [↑](#endnote-ref-57)
58. Van Hout B, Janssen MF, Feng YS, Kohlmann T, Busschbach J, Golicki D, et al.. Interim scoring for the EQ-5D-5L: mapping the EQ-5D-5L to EQ-5D-3L value sets. *Value in Health*. 2012;15(5): 708-715. [↑](#endnote-ref-58)
59. Bland JM, Altman DG. Statistics notes: bootstrap resampling methods. *BMJ*. 2015;350:h2622. [↑](#endnote-ref-59)
60. Briggs AH, Gray AM. Methods in health service research: Handling uncertainty in economic evaluations of healthcare interventions. *BMJ: British Medical Journal*. 1999;319(7210): 635. [↑](#endnote-ref-60)
61. Fenwick E, O'Brien BJ, Briggs A. Cost‐effectiveness acceptability curves–facts, fallacies and frequently asked questions. *Health Economics*. 2004;13(5): 405-415. [↑](#endnote-ref-61)
62. Husereau D, Drummond M, Petrou S, Carswell C, Moher D, Greenberg D, et al. Consolidated health economic evaluation reporting standards (CHEERS) statement. *International Journal of Technology Assessment in Health Care*. 2013;29(2):117-122. [↑](#endnote-ref-62)
63. Masterson-Algar P & Williams S. ‘Thrown into the deep end’: mapping the experiences of young people living in a family affected by a neurological condition. *Qualitative Health Research*. 2020;30(5): 717-729. Available from: DOI:10.1177/1049732319900498. [↑](#endnote-ref-63)
64. Lancaster GA, Dodd S, Williamson PR. Design and analysis of pilot studies: recommendations for good practice. *Journal of Evaluation in Clinical Practice*. 2002;10(2): 307–312. [↑](#endnote-ref-64)
65. Stirman SW, Baumann AA, Miller CJ. The FRAME: an expanded framework for reporting adaptations and modifications to evidence-based interventions. *Implementation Science*. 2019;14(58). Available from: DOI:10.1186/s13012-019-0898-y. [↑](#endnote-ref-65)
66. Egan KJ, Hodgson W, Imperatore G, Dunlop MD, Maguire R, Kirk A. Supporting Physical Activity for Informal Carers during and beyond COVID-19: Exploring the Feasibility, Usability and Acceptability of a Digital Health Smartphone Application,‘CareFit’. *International Journal of Environmental Research and Public Health*. 2022 Sep 30;19(19):12506. [↑](#endnote-ref-66)
67. Egan KJ, Hodgson W, Dunlop MDet al. A Novel Mobile App (“CareFit”) to Support Informal Carers to Undertake Regular Physical Activity From Home During and Beyond COVID-19 Restrictions: Co-design and Prototype Development Study. 2021. *JMIR formative research*, 5(10), e27358. [↑](#endnote-ref-67)
68. World Health Organization. (‎2016)‎. Monitoring and evaluating digital health interventions: a practical guide to conducting research and assessment. World Health Organization. https://apps.who.int/iris/handle/10665/252183. License: CC BY-NC-SA 3.0 IGO. [↑](#endnote-ref-68)
69. Glasgow RE, Harden SM, Gaglio B, et al. RE-AIM planning and evaluation framework: adapting to new science and practice with a 20-year review. *Frontiers in public health*. 2019 7, 64. [↑](#endnote-ref-69)
70. Biddle S, Mutrie N. Psychology of physical activity: Determinants, well-being and interventions. Routledge; 2007 Sep 12. [↑](#endnote-ref-70)
71. Health Research Authority and Medicines and Healthcare products Regulatory Agency. *Joint statement on seeking consent by electronic methods*. 2018. Available from: https://s3.eu-west-2.amazonaws.com/www.hra.nhs.uk/media/documents/hra-mhra-econsent-statement-sept-18.pdf [↑](#endnote-ref-71)
72. Craig C, Marshall A, Sjostrom M, et al. International physical activity questionnaire-short form. *J Am Coll Health*. 2017. 65(7), 492-501. [↑](#endnote-ref-72)
73. MacDonald B, Gibson AM, Janssen X, & Kirk A. A mixed methods evaluation of a digital intervention to improve sedentary behaviour across multiple workplace settings. *International Journal of Environmental Research and Public Health*, 17(12), 4538. [↑](#endnote-ref-73)
74. Strain T, Fitzsimons C, Kelly P, & Mutrie N. The forgotten guidelines: cross-sectional analysis of participation in muscle strengthening and balance & co-ordination activities by adults and older adults in Scotland. *BMC public health*. 2016. 16(1), 1-12. [↑](#endnote-ref-74)
75. Biddle S, Mutrie N & Gorely T. *Psychology of physical activity for health: Determinants, well-being and interventions*. 3^rd^ Ed. London: Routledge, Taylor & Francis Group 2015. [↑](#endnote-ref-75)
76. Marcus BH, Selby VC, Niaura RS, & Rossi JS, (1992). Self-efficacy and the stages of exercise behavior change. *Research quarterly for exercise and sport.* 1992. 63(1), 60-66. [↑](#endnote-ref-76)
77. Field A. Discovering statistics using IBM SPSS statistics. *sage*; 2013 Feb 20. [↑](#endnote-ref-77)
78. Amit O, Heiberger RM, Lane PW. Graphical approaches to the analysis of safety data from clinical trials. *Pharmaceutical Statistics*. 2008;7: 20-35. Available from: doi:10.1002/pst.254. [↑](#endnote-ref-78)
79. *The DEEP-Ethics Gold Standards for Dementia Research*. Produced by Innovations in Dementia. Version 1: Summer 2020. Accessible from: <https://www.dementiavoices.org.uk/wp-content/uploads/2020/07/The-DEEP-Ethics-Gold-Standards-for-Dementia-Research.pdf>

    **iSupport: A randomised controlled trial and feasibility study of the effects of an e-health intervention ‘iSupport’ for reducing distress of dementia carers**

    **HEALTH ECONOMICS ANALYSIS PLAN**

    Version: V2 Date: 29/04/24 [↑](#endnote-ref-79)
