# Supplementary material for: Cost-consequence analysis of an e-health intervention to reduce distress in dementia carers: results from the iSupport randomised controlled trial
Source: BMJ Open. 2025 May 16;15(5):e095611. doi: 10.1136/bmjopen-2024-095611 (PMC12086930; doi:10.1136/bmjopen-2024-095611)
Supplement: online supplemental file 2 [file bmjopen-15-5-s002.docx]

**iSupport: A randomised controlled trial and feasibility study of the effects of an e-health intervention ‘iSupport’ for reducing distress of dementia carers**

**HEALTH ECONOMICS ANALYSIS PLAN**

Version: V2 Date: 29/04/24

**Table of Contents**

[**1.1 Amendment history** 4](#_Toc180573341)

[**1.2 Abbreviations, acronyms and definitions of terms** 4](#_Toc180573342)

[**2 Purpose of HEAP** 5](#_Toc180573343)

[**3 Trial overview** 5](#_Toc180573344)

[**3.1 Trial background and rationale** 5](#_Toc180573345)

[**3.2 Trial aims(s)** 5](#_Toc180573346)

[**3.3 Trial objectives** 5](#_Toc180573347)

[**3.4 Trial inclusion/ exclusion criteria** 6](#_Toc180573348)

[**3.5 Intervention and comparator** 7](#_Toc180573349)

[**3.6 Trial design** 8](#_Toc180573350)

[**4 Economic analysis approach** 8](#_Toc180573351)

[**4.1 Key dates** 8](#_Toc180573352)

[**4.2 Aims of the economic evaluation** 8](#_Toc180573353)

[**4.3 Objectives of the economic evaluation** 8](#_Toc180573354)

[**4.4 Economic evaluation design** 9](#_Toc180573355)

[**4.5 Perspective(s)** 9](#_Toc180573356)

[**4.6 Time horizon and discount rates** 9](#_Toc180573357)

[**4.7 Data entry and management** 9](#_Toc180573358)

[**4.8 Data validation and cleaning** 9](#_Toc180573359)

[**4.9 Statistical software** 9](#_Toc180573360)

[**4.10 Costing the intervention** 9](#_Toc180573361)

[**4.11 Measurement and valuation of resource use data** 10](#_Toc180573362)

[**4.12 Measurement and valuation of outcome data** 10](#_Toc180573363)

[EQ-5D-5L 11](#_Toc180573364)

[DEMQOL-proxy 11](#_Toc180573365)

[ZBI-12 11](#_Toc180573366)

[CES-D10 11](#_Toc180573367)

[**4.13 Population for the base-case economic analysis** 12](#_Toc180573368)

[**4.13 Reporting checklists/standards** 12](#_Toc180573369)

[**5 Cost-effectiveness analysis** 12](#_Toc180573370)

[**5.1 Missing data** 12](#_Toc180573371)

[**5.2 Analysis of costs** 12](#_Toc180573372)

[**5.3 Analysis of outcomes** 12](#_Toc180573373)

[**5.4 Analysis of cost-effectiveness** 12](#_Toc180573374)

[**5.5 Handling uncertainty** 13](#_Toc180573375)

[**5.6 Subgroup analysis** 14](#_Toc180573376)

[**5.7 Sensitivity analysis** 14](#_Toc180573377)

[**8 References** 15](#_Toc180573378)

**1.1 Amendment history**

| **HEAP Version No.** | **Date issued** | **Author(s) of changes** | **Details of changes made** |
| --- | --- | --- | --- |
| V1 | 06/10/23 | RTE, BFA, KD | Deviation to the protocol – as the main trial did not demonstrate significant differences in effectiveness, the health economics team will now conduct a cost-consequence analysis. Our base case analysis will use the proxy good method to calculate informal care costs and we will also conduct a separate sensitivity analysis using the opportunity costs method to explore variation in costs. We will use total time spend accessing iSupport for our sub group analysis instead of number of times a carers has logged on to iSupport. |

**1.2 Abbreviations, acronyms and definitions of terms**

| **Acronym** | **Meaning** |
| --- | --- |
| CCA | Cost-consequence analysis |
| CEAC | Cost-effectiveness Acceptability Curve |
| CHEME | Centre for Health Economics and Medicines Evaluation, Bangor University |
| CRF | Case Report Form- the questionnaire used in participant interviews |
| Dyad | Refers to the carer-care recipient pair |
| HEAP | Health Economics Analysis Plan |
| HRQoL | Health related Quality of Life |
| ICER | Incremental Cost-effectiveness Ratio |
| ITT | Intention To Treat |
| NIHR | National Institute for Health Research |
| QALY | Quality-adjusted Life Year |
| SAP | Statistical Analysis Plan |

**2 Purpose of HEAP**

The purpose of this HEAP is to describe the analysis and reporting procedure intended for the economic evaluation of iSupport. The analysis plan is designed to ensure that there is no conflict with the protocol and associated statistical analysis plan (SAP) and it should be read in conjunction with them.

**3 Trial overview**

Section 3 is a condensed extract from protocol version: v1, 10/03/2021.

**3.1 Trial background and rationale**

There are approximately 850,000 people living with dementia in the UK and the majority of these people are cared for at home with the support of family or friends (Lewis et al., 2014). Unfortunately, the caring role is often associated with detrimental impacts on the physical and mental health of dementia carers (Gilhooly et al., 2016). In the UK, the total estimated cost of dementia to society is £26.3 billion per year (Alzheimer’s Society, 2014). The work of unpaid carers contribute £11.6 billion annually to the cost of dementia which is significantly higher than the £4.3 billion contributed by the NHS for healthcare costs and also the £10.3 billion spent on social care (Alzheimer’s Society, 2014). Due to the significant financial contribution provided by informal carers coupled with the negative health consequences related to the caring role, the global action plan on the public health response to dementia 2017-2025 have outlined their goal to prioritise support for carers of people living with dementia (Action area 5). The action area sets out to provide accessible evidence-based information to carers to facilitate knowledge and skills in order to prevent stress and health problems among carers (World Health Organisation, 2017).

Although health is a devolved area of government policy, UK national dementia strategies all make commitments to support the health and wellbeing of dementia carers (Welsh Government, 2018; Scottish Government, 2017; Department of Health, 2009). NICE recommend informal carers of people living with dementia should be offered training and psychoeducation to help them develop care skills and manage their own physical and mental health (NICE, 2018). This is especially important given the current pandemic, when many carers no longer have access to usual respite, leisure, and support services, finding themselves distanced and isolated.

**3.2 Trial aims(s)**

To test the clinical and cost-effectiveness of the iSupport intervention for carers of people living with dementia.

**3.3 Trial objectives**

WS1. A definitive pragmatic individually randomised controlled trial across Wales, Scotland and England, with a six-month nested internal pilot. This was conducted to:

- Determine progression of the definitive trial based on a go/review/stop criteria (nested internal pilot).
- Determine the effectiveness of ‘iSupport’ in reducing symptoms of distress and/or depression.
- Determine the effectiveness of ‘iSupport’ in reducing symptoms of anxiety.
- Determine the effectiveness of ‘iSupport’ in improving dementia knowledge, relationship quality and resilience.
- Describe the trial sample according to demographic/socioeconomic characteristics.

WS2. A process evaluation to determine the barriers and facilitators to the implementation of ‘iSupport’ at scale, and the extent it supports carers in the face of the ongoing or future COVID-19 pandemic. This will:

• Determine participant engagement and adherence to ‘iSupport’.

• Explore the mechanisms of change.

• Identify the external factors to ‘iSupport’ which influence the delivery and function of the intervention.

• Explore the contextual factors that influence the scalability of ‘iSupport’ into wider contexts using the CICI framework (Pfadenhauer et al., 2017).

WS3. A parallel cost-effectiveness analysis, undertaken from a public sector plus opportunity costs perspective. See sections 4 and 5 for full details of the economic evaluation.

- If the main trial does not demonstrate a significant difference in outcome measures, we will undertake a cost-consequence analysis.

WS4. A non-randomised feasibility study of intervention refinement for younger dementia carers (aged 11-17). This will:

- Explore the potential of ‘iSupport’ to address the required support that is unique to young carers, including the potential of the platform in the face of the ongoing or future COVID-19 pandemic.
- Work with young carers to refine ‘iSupport’ to fit their needs.
- Identify what outcomes are most important and relevant to young carers in relation to ‘iSupport’.
- Identify the best ways to increase the accessibility and uptake of ‘iSupport’ for young carers.
- Explore the feasibility of the refined ‘iSupport’ intervention.

**3.4 Trial inclusion/ exclusion criteria**

**Inclusion criteria**

1) Adults (18+) who self-identify as an unpaid carer (partners, children, friends, etc.) of a person with dementia who is not living in a full-time care facility, caring at least weekly for at least 6 months.

2) Self-identify as experiencing at least some stress, depression or anxiety.

3) The care recipient has to have a confirmed diagnosis of dementia (through self-report of the carer, to reflect the ‘real world’ application of ‘iSupport’).

**Exclusion criteria**

1) Receiving psychological treatment from a mental health specialist at the time of recruitment.

2) Unable to comprehend written English.

3) No access to the internet.

4) Unable to give informed consent to the trial.

5) Have previously used ‘iSupport’ materials (in the last 12 months).

**3.5 Intervention and comparator**

‘iSupport’ is an internet-based psychoeducation and skills development intervention. The theoretical underpinnings of ‘iSupport’ are based on person-centred care, which recognises that dementia care should reflect the individual’s needs, personality and ability (Kitwood, 1997).

‘iSupport’ consists of five main themes (see Figure 1 below) and twenty-three accompanying exercises, namely: (i) introduction to dementia; (ii) being a carer; (iii) caring for me; (iv) providing everyday care; and (v) dealing with behaviour changes. Each exercise takes approximately 5-15 minutes and follows the same format: information about a topic presented; short interactive exercises and questions with instant feedback on responses; a summary of the lesson; a relaxation exercise.

Carers can construct their own personalised plan and access which sessions they feel are most relevant to them at that point in time. It is anticipated the whole programme can be completed in 3 months. The programme can be followed via the internet using a personal computer or a tablet (e-health), or through a mobile phone accessing a ‘mobile friendly’ version of the platform (m-health).

To address potential inequity of uptake, a short video tutorial on how to use the programme will be developed and sent to all participants randomised to the intervention group. Participants will be advised to use ‘iSupport’ regularly in order to obtain the most benefit. They will be provided with the contact details of an ‘e-coach’, who will be trained to explain anything that is not clear about the ‘iSupport’ programme. The ‘e-coach’ will contact participants randomised to intervention shortly after randomisation, 1 month later and 2 months later (if required by the participant). We will translate ‘iSupport’ into Welsh following WHO adaptation guidelines. To improve access, we will also develop audio/read aloud function for inclusion in the platform.


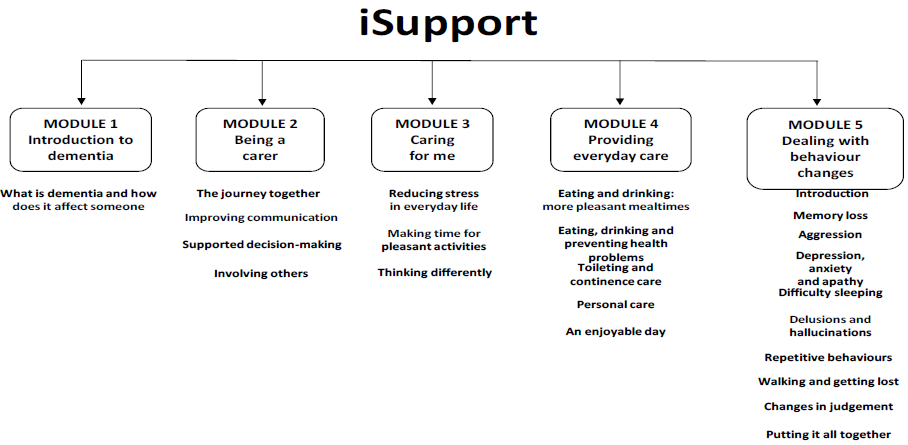


***Figure 1:*** *Overview of iSupport themes*

Participants assigned to the comparison group will receive information about dementia developed by the Alzheimer’s Society (Alzheimer’s Society, 2019). This covers the topics of understanding the diagnosis, taking on the caring role, looking ahead, understanding and supporting the person with dementia, services, support and housing, finances, the later stages of dementia, end of life care and support, contact details of useful organisations. This information will be available online and/or in printed format. Carers can choose which format they prefer. Alongside this education, carers will receive care-as-usual. They can search for other information or seek help from other providers. Information about local context support and services will be obtained at baseline. Following the final data collection the participants allocated to the comparison group will be provided with access to ‘iSupport’.

**3.6 Trial design**

WS1 is a multi-centre, pragmatic, single-blinded, two-arm randomised controlled trial (with a nested 6-month internal pilot- see the main study protocol for details of go/review/stop criteria).

Assessments will be completed at baseline (T0), 3 months after baseline (post-intervention, T1), and 6 months after baseline (follow-up, T2).

Randomisation will use a secure web-based dynamic adaptive randomisation algorithm and be stratified for site along with age and gender, previously found to influence the outcome measure of caregiver distress (Sorensen et al., 2002). If more than one person identifies as the carer for the same person (e.g. spouse/partner, adult child, or friend) we would allocate one person as the ‘index carer’ (based on caring frequency, or if caring frequency is equal between carers, then the carers will nominate who will be ‘index carer’). The economic analysis is based on power calculations of the trial in WS1. The sample size is 350 accounting for expected attrition. It is usual practice for the economic analysis to be based on the clinical power of a trial; however, it is widely acknowledged that this leads to some underpowering of economic analysis. For ethical reasons it would be unusual to power a clinical study above and beyond that needed for clinical outcomes in order to enable a fully powered economic analysis (see Briggs, 2000 and CREST, 2019).

**4 Economic analysis approach**

**4.1 Key dates**

Funding period: 01/01/2021 – 31/12/2023.

**4.2 Aims of the economic evaluation**

The aim of the economic evaluation is to address the following research question: How cost-effective is iSUPPORT relative to the provision of standardised information about dementia in terms of QALYs, carer distress levels and depression?

**4.3 Objectives of the economic evaluation**

- Undertake micro costing of the costs of implementing ‘iSupport’, including technical support and time spent supporting carers to use the tool.
- Explore patterns of, and estimate the cost of, health and social care resource use for carers in the ‘iSupport’ and comparison arms of the trial.
- Explore patterns of, and estimate the cost of, health and social care resource use for the care recipients of carers in the trial.
- Explore the opportunity cost of informal care through the measurement of informal care time, types of care task, impacts on carer’s leisure and employment hours, and carers’ willingness to pay for more support.
- Using QALYs derived from the EQ-5D-5L (Herdman et al., 2011), determine the cost-effectiveness of ‘iSupport’ compared to the control condition; conduct secondary cost-effectiveness analyses using the Zarit Burden Interview-12 (ZBI-12; Bedard et al., 2001), the Centre for Epidemiological Studies of Depression Scale (CES-D10; Radloff 1977; Andresen et al., 2013), and the DEMQOL-proxy (Mulhern et al., 2013).

**4.4 Economic evaluation design**

- Primary cost-effectiveness analysis using the ZBI-12 and CES-D10 outcomes based on the main trial
- Secondary cost-utility analysis using the EQ-5D-5L and DEMQOL-Proxy
- If the trial does not find significant differences in the primary and secondary outcomes, a cost-consequence analysis will be conducted to present the disaggregated costs and consequences of iSupport.

Details of the approach to the analyses listed above is given in Section 5.

**4.5 Perspective(s)**

Analysis will be undertaken from two perspectives; the base case analysis will adopt a public sector perspective (NHS, personal social services and local authorities) in line with NICE public health guidance (NICE, 2012), and a secondary analysis will be undertaken from a societal perspective using an opportunity cost method.

**4.6 Time horizon and discount rates**

The primary economic analysis will compare the costs and consequences of each arm over 6 months. No discounting of costs or outcomes will be applied.

**4.7 Data entry and management**

Data collection will take place by telephone or internet-based service such as Zoom, Teams or Skype. Interviewers will record answers on paper-based Case Report Forms (CRFs), and responses will then be input on the MACRO data entry system. NWORTH will manage the database and provide extracts of the trial data to the health economics team on request.

**4.8 Data validation and cleaning**

Face validity checks will be conducted on the data to identify typos and numerical outliers. Discrepancies will be checked against the source documents and a log of errors kept. The health economists will notify NWORTH of any errors identified.

**4.9 Statistical software**

Cost-effectiveness analyses will be conducted using Microsoft Excel 2016, Stata version 17.0 or Statistical Package for Social Sciences (SPSS).

**4.10 Costing the intervention**

We will calculate the costs of implementing iSupport, including technical support time and time spent by the e-coaches supporting carers to use the tool.

Research and development costs will not be included e.g. training time spent instructing staff on questionnaires, time spent arranging interviews with participants, time spent completing questionnaires.

**4.11 Measurement and valuation of resource use data**

Resource use data will be collected using a bespoke Service Use measure. Carers’ frequency of contacts with hospital services, day services and selected community-based services will be collected at baseline, 3 months and 6 months. Carers will also report on the service use of the person being cared for at the same time-points, and whether or not they accompanied the person being cared for to their appointments. Service use data will be costed in £ pounds sterling using national unit costs (Curtis and Burns, 2020; NHS Improvement, 2020).

To measure opportunity costs, we will ask carers to report on changes in types of care tasks undertaken during the last week, measured at baseline, 3-months and 6-months follow-up. The Erasumus iMTA informal care questionnaire will be adapted for this (Hoefman et al., 2011). We will explore the opportunity costs of undertaking informal care tasks (household activities, personal care and practical support) using two methods: the proxy good method, and the opportunity cost method. Pages 15-21 of the iMTA manual contains guidance on costing informal care time using these various methods (Hoefman et al., 2011). Our base case analysis will calculate informal care costs using the proxy good method (also known as the replacement cost method) regards work from the perspective of society and requires multiplying the number of hours spent completing informal care task by a shadow price market substitute. To explore variations in informal care costs, we will undertake a sensitivity analysis using the opportunity cost method. Opportunity costs of informal care time will be calculated based on salary information obtained from the UK Government careers website, the PSSRU (Jones et al., 2022), and other online job market websites where necessary (where job salary information is not available for some occupations reported in the data) to source to cost an hourly rate for informal care tasks based on the occupational role details provided by participants. For retired participants, opportunity costs will be valued as a proxy cost of lost leisure time.

There is no clear consensus on how informal care should be included in the economic evaluation of interventions; however, previous studies have included cost per QALY estimates both with and without informal care costs (Goodrich et al., 2012). Carers may find it difficult to differentiate between ‘normal’ and caregiving’ activities (Urwin et al., 2021). Consequently, due to the potential for coresident carers to report large numbers of hours for unpaid care (e.g., for purposes of supervision when required rather than for time conducting other tasks), we will conduct sensitivity analysis around the number of unpaid care hours costed (Goodrich, Kaambwa and Al-Janabi, 2012).

**4.12 Measurement and valuation of outcome data**

We will follow the lead of the main trial primary outcomes and we will conduct a subsequent cost-utility analysis if a signifianct difference is demonstrated in the outcome measures. The cost-effectiveness analysis will use the ZBI-12 and CES-D10 and the cost-utility analysis will use the EQ-5D-5L and DEMQOL-proxy. If the main trail does not demonstrate a significance difference in effectiveness a cost-consequence analysis will be conducted to present the disaggregated costs and outcomes of the iSupport trial.

The primary outcomes for the effectiveness analysis are the ZBI-12 (Bedard et al., 2001) and CES-D10 (Radloff, 1977; Andreson et al., 2013). Secondary outcome measures include the Generalised Anxiety Disorder Questionnaire (GAD-7; Spitzer et al., 2006), Resilience Scale-14 (RS-14; Wagnild, 2009), Quality of the carer-patient relationship (QCPR; Spruytte et al., 2000), dementia knowledge (DKAS; Annear et al., 2017), EQ-5D-5L (Herdman et al., 2011), and DEMQOL-proxy (Mulhern et al., 2013).

EQ-5D-5L

The EQ-5D-5L is a generic, preference based, health-related quality of life (HRQoL) measure (Herdman et al., 2011). It consists of two parts, a five-item questionnaire and a visual analogue scale (EQ-VAS).

The first section contains a set of five multiple choice questions, asking the respondent about the level of difficulty they have in the following domains: Mobility, Self-care, Usual activities, Pain / discomfort, Anxiety / depression. Each item has three possible answer options scored 1-5, where 1 indicates no problems in that domain, and 5 indicates extreme problems/unable to perform. The second section of the EQ-5D-5L asks the participant to rate their overall health using a vertical visual-analogue scale, where health is rated anywhere between 0 (worst imaginable health) and 100 (best imaginable health).

DEMQOL-proxy

The DEMQOL-proxy is a 31-item dementia specific, preference based, health-related quality of life measure (Mulhern et al., 2013) used with carers to report on the quality of life of the person being cared for. There are four levels of response for each item, ranging from ‘a lot’ to ‘not at all’. An algorithm is used to convert responses into an index score of between 0.363 and 0.937 for the proxy version.

ZBI-12

A 12-item measure to assess carer distress. Item responses range from 0 (never) to 4 (almost always), and higher scores indicate greater distress. The original 22-item ZBI is used widely in research with dementia caregivers and internal consistency of the 12-item version, as measured by Cronbach’s alpha, is α =.85 (O’Rourke and Tuokko, 2003). Concurrent validity of responses to the 12-item version has been examined and found to be good relative to indices of patient behavioural disturbance and Activities of Daily Living (ADL) impairment in dementia (Bedard et al., 2001).

CES-D10

The Centre for Epidemiological Studies of Depression Scale (CES-D10; Andreson et al., 2013), is a widely used 10-item measure of depression. Ratings relate to the past week with eight items measuring frequency of depressive symptoms and two measuring positive affect. Response categories range from 0 (rarely or none of the time present) to 3 (most or all of the time present). Scores range from 0 (no depression) to 30 (very depressed). CES-D is a valid and reliable scale for detecting caregiver depression in dementia.

**4.13 Population for the base-case economic analysis**

The trial will recruit 350 participants. The economic analysis will adopt an Intention to Treat (ITT) approach, where all randomised participants will be included.

**4.13 Reporting checklists/standards**

The cost-effectiveness analysis will be reported according to the Consolidated Health Economic Evaluation Reporting Standards (CHEERS; Husereau et al., 2022).

**5 Cost-effectiveness analysis**

**5.1 Missing data**

Missing data will be handled in accordance with the Statistical Analysis Plan (SAP), with the exception of missing resource use data, which will not be imputed. For our main analysis, we will conduct a complete case analysis of E5-5D-5L data but we will explore the possibility of imputation.

**5.2 Analysis of costs**

Differences in overall mean costs between the arms (adjusted for baseline differences) will be presented. We will also bootstrap (5,000 replications) the difference in mean total cost to see if this is a statistically significant difference. Our priority is to cost the within trial service use and we will reflect on the equivalent or otherwise service use prior to the beginning of the trial.

**5.3 Analysis of outcomes**

The outcomes for the cost-utility analysis will be quality-adjusted life years (QALYs) at 6 months. Utility values will be obtained from responses to the EQ-5D-5L at baseline, 3 months and 6 months for the primary analysis. A scoring algorithm using UK tariff values will be used to convert responses into an index score of between -0.594 and 1, with 1 representing full HRQoL. An appropriate regression model will be used to adjust for imbalances in baseline utility.

The cost-effectiveness analysis will use the ZBI-12 and CES-D10 and the cost-utility analysis will use the EQ-5D-5L and DEMQOL-proxy. Our secondary analysis using imputation will use data from the SAP. We will calculate the cost per unit change in carer distress using the 12-item Zarit Burden Interview, cost per unit change in carer anxiety and depression using the 10-item CES-D, and dementia-related quality of life of the person living with dementia, as rated by the carer, using the DEMQOL-proxy responses converted into an index score of between 0.363 and 0.937. We will express a cost per change for a 4-point difference on the ZBI scale and a 2-point difference on the CES-D10.

**5.4 Analysis of cost-effectiveness**

Cost and outcomes data will be combined to calculate an incremental cost-effectiveness ratio (ICER):

C1= cost in intervention group

C0= cost in control group

E1= effect in intervention group

E0= effect in control group

$$ICER =\frac{(C1-C0)}{(E1-E0)}$$

**5.5 Handling uncertainty**

The nonparametric bootstrapping approach (Bland and Altman, 2015; Briggs and Gray, 1999) will be used to determine the level of sampling uncertainty surrounding the mean ICER by generating 5,000 estimates of incremental costs and benefits. A cost-effectiveness plane such as the one in Figure 2 will be plotted to illustrate where the distribution of the estimates fall.


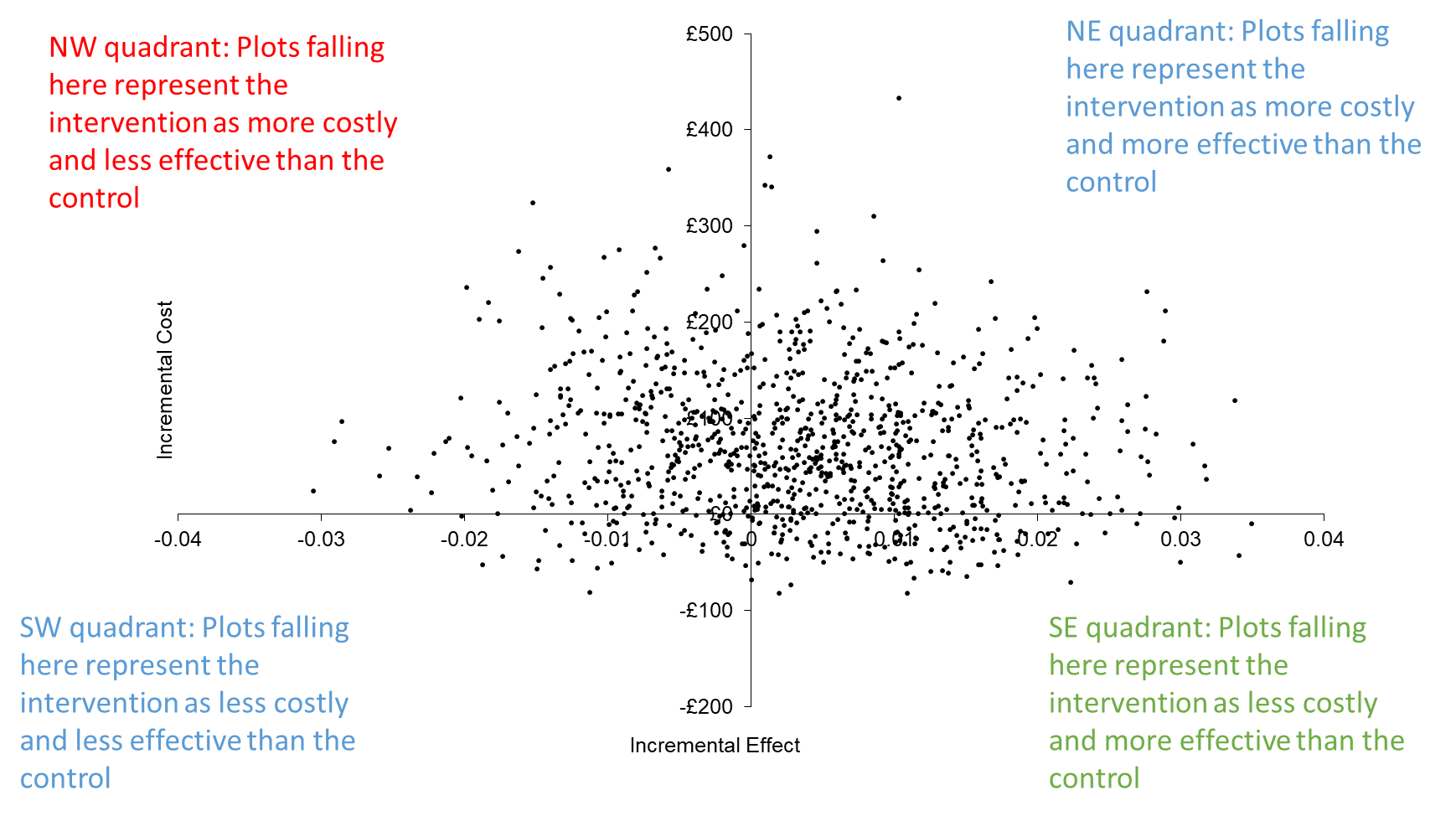


***Figure 2:*** *Example cost-effectiveness plane*

A cost-effectiveness acceptability curve (CEAC) will be plotted to represent the probability that iSupport will be cost-effective at any given threshold (Fenwick et al., 2004). For example, in the CEAC in Figure 3 the probability that the intervention is cost-effective at a threshold of £30,000 per QALY gained is 55%.


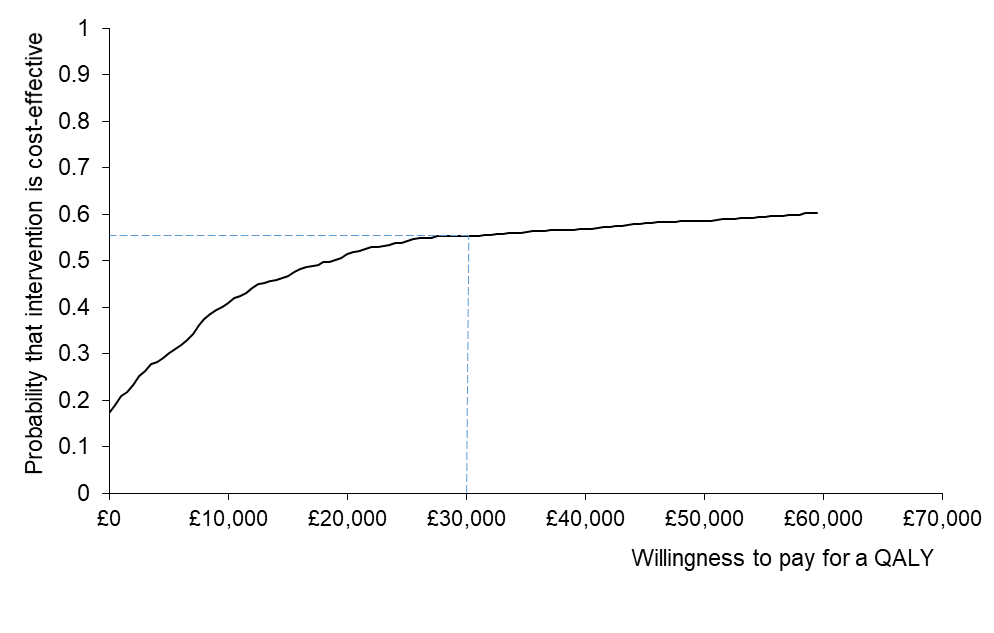


***Figure 3:*** *Example cost-effectiveness acceptability curve*

**5.6 Subgroup analysis**

A subgroup analysis will be conducted on the total length of time that carers in the intervention group access ‘iSupport’ (low/ moderate/ high user categories to be classified when the health economics team access the data).

Possible subgroup analysis will be considered for the following variables: gender, age and dyad relationship categories (spouses / siblings/ child-parent/ parent-child/ friends/ other). The scope for subgroup analysis will be constrained by the size of the subgroups however, useful lessons can be made with the caveat that they are underpowered.

**5.7 Sensitivity analysis**

The results for complete cost and quality of life data (i.e. those with no missing data) will be provided to identify the impact of missing data on the analysis.

Sensitivity analyses will be conducted to vary the costs of inputs (e.g. the cost of the staff supporting carers to use ‘iSupport’). For example, we will explore the impact on costs from varying our assumptions on e-coach time spent supporting participants to use the platform.

A sensitivity analysis will plot and describe the outliers with high-cost resource use will be carried out. We will look at extreme outliers where participant service use is not related to their dementia caring role.

Our base case analysis will use the proxy good method to calculate informal care time costs. We will also complete a sensitivity analysis using the opportunity cost method to explore variations in costs using this method.

**8 References**

Alzheimer’s Society. *Dementia UK. Update*. 2014. Available from: https://www.alzheimers.org.uk/sites/default/files/migrate/downloads/dementia_uk_update.pdf

Alzheimer’s Society. *Caring for a person with dementia: A practical guide*. 2019. Available from: https://www.alzheimers.org.uk/get-support/publications-factsheets/caring-person-dementia-practical-guide

Andresen EM, Byers K, Friary J, Kosloski K, Montgomery R. Performance of the 10-item Center for Epidemiologic Studies Depression scale for caregiving research. *SAGE Open Medicine*. 2013. Available from: https://doi.org/10.1177/2050312113514576.

Annear MJ, Toye C, Elliott KJ, McInerney F, Eccleston C, Robinson, A. Dementia knowledge assessment scale (DKAS): confirmatory factor analysis and comparative subscale scores among an international cohort. *BMC Geriatrics*. 2017;17(168). Available from: doi:10.1186/s12877-017-0552-y.

Bédard M, Molloy DW, Squire L, Dubois S, Lever JA, O'Donnell M. The Zarit Burden Interview: a new short version and screening version. *Gerontologist*. 2001;41(5):652-657.

Bland JM, Altman DG. Statistics notes: bootstrap resampling methods. bmj. 2015 Jun 2;350:h2622.

Briggs AH, Gray AM. Methods in health service research: Handling uncertainty in economic evaluations of healthcare interventions. BMJ: British Medical Journal. 1999 Sep 4;319(7210):635.

Curtis L and Burns A. Unit costs of health and social care 2020. Canterbury: Personal Social Services Research Unit, University of Kent; 2017.

Department of Health. *Living well with dementia: A national dementia strategy*. 2009. Available from: https://www.gov.uk/government/publications/living-well-with-dementia-a-national-dementia-strategy

Fenwick E, O'Brien BJ, Briggs A. Cost‐effectiveness acceptability curves–facts, fallacies and frequently asked questions. Health economics. 2004 May 1;13(5):405-15.

Gilhooly KJ, Gilhooly MLM, Sullivan MP, et al. A meta-review of stress, coping and interventions in dementia and dementia caregiving. *BMC Geriatrics*. 2016;16(106). Available from: doi:10.1186/s12877-016-0280-8.

Goodrich, K., Kaambwa, B. and Al-Janabi, H., 2012. The inclusion of informal care in applied economic evaluation: a review. Value in Health, 15(6), pp.975-981.

Herdman M, Gudex C, Lloyd A, Janssen MF, Kind P, Parkin D, Bonsel G, Badia X. Development and preliminary testing of the new five-level version of EQ-5D (EQ-5D-5L). Quality of life research. 2011 Dec 1;20(10):1727-36.

Hoefman RJ, Van Exel NJA, Brouwer WBF. *iMTA Valuation of Informal Care Questionnaire (iVICQ)*. Version 1.0 (December 2011). Rotterdam: iBMG / iMTA, 2011.

Husereau D, Drummond M, Augustovski F, de Bekker-Grob E, Briggs AH, Carswell C, Caulley L, Chaiyakunapruk N, Greenberg D, Loder E, Mauskopf J. Consolidated Health Economic Evaluation Reporting Standards 2022 (CHEERS 2022) statement: updated reporting guidance for health economic evaluations. International Journal of Technology Assessment in Health Care. 2022;38(1).

Kitwood, T. *Dementia reconsidered: The person comes first*. England: Open University Press; 1997.

Lewis, et al., *Trajectory of Dementia in the UK – Making a Difference*. Office of Health Economics for Alzheimer’s Research UK 2014. Available from: https://www.alzheimersresearchuk.org/wp-content/uploads/2015/01/OHE-report-Full.pdf

Pfadenhauer LM, Gerhardus A, Mozygemba K, et al. Making sense of complexity in context and implementation: the Context and Implementation of Complex Interventions (CICI) framework. *Implementation Science*. 2017;12(21). ISSN 1748-5908.

Mulhern B, Rowen D, Brazier J, Smith S, Romeo R, Tait R, et al. Development of DEMQOL-U and DEMQOL-PROXY-U: generation of preference-based indices from DEMQOL and DEMQOL-PROXY for use in economic evaluation. Health technology assessment (Winchester, England). 2013 Feb;17(5):v-xv, 1-140.

NHS Improvement. *National Cost Collection for the NHS*. https://improvement.nhs.uk/resources/national-cost-collection/ [Last accessed 10th March 2020].

National Institute for Health and Care Excellence. Methods for the development of NICE public health guidance (third edition). 2012. Available at <https://www.nice.org.uk/process/pmg4/chapter/introduction> [Last accessed 13/04/18].

National Institute of Health and Care Excellence (NICE). *Dementia: assessment, management and support for people living with dementia and their carers*. 2018. Available from: https://www.nice.org.uk/guidance/ng97

O’Rourke N, Tuokko HA. Psychometric Properties of an Abridged Version of the Zarit Burden Interview Within a Representative Canadian Caregiver Sample. *The Gerontologist*. 2003;43(1):121-127.

Radloff LS. CES-D scale: a self report depressions scale for research in the general populations. *Applied Psychological Measurement*. 1977;1(3): 385–401.

Scottish Government. *National Dementia Strategy, 2017-2020*. 2017. Available from: https://www.gov.scot/publications/scotlands-national-dementia-strategy-2017-2020/

Sörensen S, Pinquart M, Duberstein, P. How effective are interventions with caregivers? An updated meta-analysis. *Gerontologist*. 2002;42(3): 356-72.

Spitzer R, Kroenke K, Williams JBW, Lowe B. A brief measure for assessing generalised anxiety disorder. *Archives of Internal Medicine*. 2006;166(10):1092-1097.

Spruytte N, Van Audenhove C, Lammertyn F. Internal Report: *The Scale for the Quality of the Current Relationship*. Leuven: LUCAS-KULeuven. 2000.

Urwin, S., Lau, Y.S., Grande, G. and Sutton, M., 2021. The extent and predictors of discrepancy between provider and recipient reports of informal caregiving. Social Science & Medicine, 277, p.113890.

Wagnild GM. *The Resilience Scale User’s Guide for the US English version of the Resilience Scale and the 14-item Resilience Scale (RS-14)*. The Resilience Center, Montana. 2009.

Welsh Government. *Dementia Action Plan for Wales, 2018-2022*. 2018. Available from: https://gov.wales/sites/default/files/publications/2019-04/dementia-action-plan-for-wales.pdf

World Health Organisation. *Global action plan on the public health response to dementia 2017–2025*. 2017. Available from: https://apps.who.int/iris/bitstream/handle/10665/259615/9789241513487-eng.pdf;sequence=1

iSupport
