# Supplementary material for: Cost-consequence analysis of an e-health intervention to reduce distress in dementia carers: results from the iSupport randomised controlled trial
Source: BMJ Open. 2025 May 16;15(5):e095611. doi: 10.1136/bmjopen-2024-095611 (PMC12086930; doi:10.1136/bmjopen-2024-095611)
Supplement: online supplemental file 4 [file bmjopen-15-5-s004.docx]

**Table of contents**

| **Supplementary material:** | **Page numbers:** |
| --- | --- |
| Supplementary Table 1: Costs of e-coach tasks and materials for iSupport group and control group. | 2 |
| Supplementary Table 2: Unit cost schedule – public sector perspective. | 3-11 |
| Supplementary Table 3: Unit cost schedule – wider perspective. | 12 |
| References of unit cost sources. | 13 |
| Informal care cost approach – opportunity cost method (including sources of costs for opportunity cost method). | 14 |
| Supplementary Table 4: Baseline characteristics of complete cases EQ-5D-5L participants (n=261). | 15 |
| Supplementary Table 5: Frequencies of carer resource use (public sector perspective). | 16 |
| Supplementary Table 6: Subgroup analysis on the total time spent on the iSupport platform from NHS perspective. | 17 |
| Supplementary Table 7: Total resource use costs for patients living with dementia from the public sector and wider societal perspective. | 17 |
| Supplementary Table 8. Mean costs per participant of hospital, community and other services for people living with dementia from the public sector perspective (£, 95% CI). | 18 |
| Supplementary Table 9: Total and mean (SD) informal care time reported by carers (hours). | 18 |
| Supplementary Table 10: Total group costs of resource use and informal care for carers from the wider societal perspective incorporating the opportunity cost method. | 19 |
| Supplementary Table 11: Difference in mean costs per participant of resource use and informal care for carers (opportunity cost method) from the wider societal perspective (£, 95% CI). | 20 |
| Supplementary Table 12. Mean costs per participant of hospital, community and other services for people living with dementia from the wider societal perspective (£, 95% CI). | 21 |
| Supplementary Table 13: Sensitivity analysis (removing outliers) reporting on the difference in mean costs per participant from the public sector and societal perspective (£, 95% CI) | 22 |
| Supplementary Table 14: Number of outliers that were removed from the sensitivity analysis (n). | 23 |
| Supplementary Table 15: Total group costs of resource use and informal care for carers (public sector and wider societal perspective). | 24 |

**Supplementary Table 1: Costs of e-coach tasks and materials for iSupport group and control group**

| **E-coach training and role preparation tasks for iSupport** | **Average duration to complete task (minutes)** | **Quantity of tasks completed** | **Total time completing task (minutes)** | **Total costs (salary including on-costs)** | **Total cost with overheads (30% uplift)** |
| --- | --- | --- | --- | --- | --- |
| Induction, training and reading protocol | 435 | 4 | 1740 | £591.60 | £769.08 |
| Meeting with manager to discuss emails and other aspect of e-coach role | 30 | 68 | 2040 | £693.60 | £901.68 |
| Updating participant contact information records | 45 | 40 | 1800 | £612.00 | £795.60 |
| Drafting bi-lingual template emails for contact attempts | 45 | 4 | 180 | £61.20 | £79.56 |
| **Totals:** |  | **116** | **5760** | £1,958.40 | £2,545.92 |
| **E-coach intervention delivery tasks for iSupport** | **Average duration to complete task (minutes)** | **Quantity of tasks completed** | **Total time completing task (minutes)** | **Total cost (salary and oncosts)** | **Total cost with overheads (30% uplift)** |
| Sending out first invitation email | 2 | 156 | 312 | £106.08 | £137.90 |
| Sending out 1-month check-in email | 2 | 168 | 336 | £114.24 | £148.51 |
| Sending out 2-month check-in email to inactive participants | 2 | 63 | 126 | £42.84 | £55.69 |
| Drafting miscellaneous email templates and sending five emails | 11 | 5 | 55 | £18.70 | £24.31 |
| Loading email platform and check shared inbox | 1 | 152 | 152 | £51.68 | £67.18 |
| Reading emails from participants (queries or general points/feedback) | 2 | 137 | 274 | £93.16 | £121.11 |
| Providing email responses to participants for specific queries | 30 | 80 | 2400 | £816.00 | £1,060.80 |
| Giving assistance to participants over the telephone | 20 | 2 | 40 | £13.60 | £17.68 |
| Monthly checking of the iSupport platform for participant inactivity | 30 | 16 | 480 | £163.20 | £212.16 |
| **Totals:** |  | **779** | **4175** | **£1,419.50** | **£1,845.35** |
| **E-coach delivery tasks for control group** | **Average duration to complete task (minutes)** | **Quantity of tasks completed** | **Total time completing task (minutes)** | **Total cost (salary and oncosts)** | **Total cost with overheads (30% uplift)** |
| Sending emails | 2 | 176 | 352 | 119.68 | 155.584 |
| Reading emails of questions or general points/feedback | 2 | 73 | 146 | 49.64 | 64.532 |
| **Total** |  | 249 | 498 | 169.32 | 220.116 |
| **Materials sent to control group** | **Cost per item** | **Postage per item** | **Total cost** |  |  |
| Alzheimer society booklet (PDF version) sent to all participants in control group | £0.00 | NA | £0.00 |  |  |
| Alzheimer society booklet (paper version) sent out to 39 participants who requested a physical copy | NA | £2.99 | £116.61 |  |  |
| **Total:** |  |  | £116.61 |  |  |

**Supplementary Table 2: Unit cost schedule – public sector perspective**

| **Community-based resource use:** | **Unit cost** | **Unit** | **Source** |
| --- | --- | --- | --- |
| GP clinic visit | £41.00 | per visit | Per surgery/clinic contact lasting on average 9.22 minutes. Includes qualification costs and direct care staff costs (PSSRU, 2022, p.70). |
| GP home visit | £132.50 | per visit | Per patient contact lasting 30 minutes (assumption based on clinic visit being 9.22 minutes). Includes qualification costs and direct care staff costs (PSSRU, 2022, p.70). |
| GP telephone | £15.50 | per intervention | Per intervention lasting 4 minutes. Includes other costs (PSSRU, 2022, p.73). |
| Nurse clinic visit | £52.00 | per hour | Unit cost per hour (Band 5 practice nurse). Includes qualification costs (PSSRU, 2022, p.68). |
| Nurse home visit | £52.00 | per hour | Assumed the same as clinic visit. Unit cost per hour (Band 5 practice nurse). Includes qualification costs (PSSRU, 2022, p.68). |
| Nurse telephone | £8.69 | per intervention | Per intervention lasting 6.56 minutes. Band 5 nurse. Includes other costs (PSSRU, 2022, p.73). |
| Mental health team worker clinic visit | £52.00 | per hour | Unit cost per working hour (with qualifications) Band 5 community-based nurse (PSSRU, 2022, p.66). |
| Mental health team worker home visit | £52.00 | per hour | Assumed home visit is the same as clinic visit. Unit cost per hour (Band 5). Includes qualification costs (PSSRU, 2022, p.68). |
| Mental health team worker telephone | £8.69 | per intervention | Assumed same as Band 5 nurse-led telephone call lasting 6.56 minutes. Includes other costs (PSSRU, 2022, p.73). |
| Psychiatrist clinic visit | £145.00 | per hour | Costed as a Consultant psychiatric, with qualifications (PSSRU, 2022, p.101). |
| Psychiatrist home visit | £145.00 | per hour | Assumed home visit is the same as clinic visit. Costed as a Consultant psychiatric, with Qualifications (PSSRU, 2022, p.101). |
| Psychiatrist telephone | £36.25 | per contact | Assumed 15 minutes per telephone contact, £145 per hour. Costed as a Consultant psychiatric, with Qualifications (PSSRU, 2022, p.101). |
| Psychologist clinic visit | £66.00 | per hour | Unit cost per working hour Band 7 clinical psychologist (PSSRU, 2022, p.60). |
| Psychologist home visit | £66.00 . | per hour | Assumed home visit is same as clinic visit. £66 per working hour Band 7 clinical psychologist (PSSRU, 2022, p.60). |
| Psychologist telephone | £16.50 | per contact | Assumed 15 minutes per telephone contact, £66 per working hour. Band 7 clinical psychologist (PSSRU, 2022, p.60). |
| Counsellor clinic visit | £55.00 | per hour | Unit cost per working hour, Band 6 counsellor (PSSRU, 2022, p.60). |
| Counsellor home visit | £55.00 | per hour | Assumed home visit is same as clinic visit. Unit cost per working hour, Band 6 counsellor (PSSRU, 2022, p.60). |
| Counsellor telephone | £13.75 | per contact | Assumed 15 minutes per telephone contact. £55 per hour Band 6 counsellor (PSSRU, 2022. p.60). |
| Social worker clinic visit | £50.00 | per hour | Cost per hour, including qualifications social worker adult services (PSSRU, 2022, p.81). |
| Social worker home visit | £50.00 | per hour | Assumed home visit is same as clinic visit. Cost per hour, including qualifications social worker adult services (PSSRU, 2022, p.81). |
| Social worker telephone | £12.50 | per contact | Assumed 15 minutes per telephone contact. £50 per hour social worker (PSSRU 2022, p.81). |
| Occupational therapist clinic visit | £50.00 | per hour | Unit cost per hour (with qualifications) community occupational therapist (PSSRU, 2022, p.83). |
| Occupational therapist home visit | £50.00 | per hour | Assumed same cost as clinic visit. Unit cost per hour (with qualifications) community occupational therapist (PSSRU, 2022, p.83). |
| Occupational therapist telephone | £12.50 | per contact | Assumed 15 minutes per online/telephone contact. £50 per hour community occupational therapist (PSSRU, 2022, p.83). |
| Support/education group clinic/centre visit | £25.00 | per hour | Unit cost per hour support and outreach worker (PSSRU, 2022, p.86). |
| Support/education group home visit | £25.00 | per hour | Assumed same cost as clinic/centre visit. Unit cost per hour support and outreach worker (PSSRU, 2022, p.86). |
| Support/education group telephone | £6.25 | per contact | Assumed 15 minutes per telephone contact. £25 per hour (PSSRU, 2022, p.86). |
| Memory clinic | £576.00 | per hour | Dementia memory service, p.28 PSSRU 2021, Cost per hour £528 inflated to £576 for cost year 2022. |
| Memory clinic telephone | £8.69 | per contact | Assumed same as Band 5 nurse-led telephone call lasting 6.56 minutes. Includes other costs (PSSRU, 2022, p.73). |
| **Emergency care:** | **Unit cost** | **Unit:** | **Source/notes:** |
| Accident and Emergency | £144.00 | unit cost | NHS reference costs 2021/22. (Total Outpatient Attendance) Emergency Medicine Service (service code 180) |
| Ambulance: | £268.39 | unit cost | NHS reference costs 2021/22 (Ambulance) See & Treat. |
| Rapid/medical/surgical assessment unit | £144.00 | unit cost | NHS reference costs 2021/22. (Total Outpatient Attendance) Emergency Medicine Service (service code 180) |
| Day hospital (non-specified reason for attendance) | £1,038.00 | per visit | Day case (DC), NHS unit costs 2021/22 |
| **Hospital-based telephone calls:** | **Unit cost** | **Unit:** | **Source/notes:** |
| Telephone call from hospital | £8.69 | per intervention | Assumed same as community-based nurse telephone call. Nurse-led triage, per intervention lasting 6.56 minutes. Includes other costs (PSSRU, 2022, p.73). |
| Telephone call from hospital doctor/physician/consultant | £15.50 | per intervention | Assumed same as community-based phone call with doctor. GP-led triage, per intervention lasting 4 minutes. Includes other costs (PSSRU, 2022, p.73). |
| **Day hospital:** | **Unit cost:** | **Unit:** | **Source/notes:** |
| Cataract Surgery | £1,269.47 | Unit Cost | NHS reference costs 2021/22 - Total HRGs - Phacoemulsification Cataract Extraction and Lens Implant, with CC Score 0-1 (Day case) Currency: BZ34C |
| Consultation with Surgeon (Ulcer) | £1,038.00 | Unit cost | NHS reference costs 2021/22 - Day case, Index tab: DC |
| CT scan | £104.97 | Unit Cost | NHS reference costs 2021/22 - Imaging Outpatient - Computerised Tomography Scan of One Area, without Contrast, 19 years and over (IMAGOP: RD20A) |
| Heart check-up | £1,038.00 | unit cost | NHS reference costs 2021/22 - Day case, Index tab: DC |
| Hernia Surgery | £2,715.75 | unit cost | NHS reference costs 2021/22 - Day case, Inguinal, Umbilical or Femoral Hernia Procedures, 19 years and over, with CC Score 0 Currency: FF62D |
| Immunotherapy (Kidney Cancer) | £1,038.00 | unit cost | NHS reference costs 2021/22 - Day case, Index tab: DC |
| Knee Operation | £2,080.55 | Unit cost | NHS reference costs 2021/22 - Day case, Minor Knee Procedures for Non-Trauma, 19 years and over, Currency: HN25A |
| Laser Eye Surgery | £1,399.42 | Unit cost | NHS reference costs 2021/22 - Day case, Intermediate, Cornea or Sclera Procedures, with CC Score 0, Currency: BZ64B |
| Mastectomy | £3,333.53 | Unit cost | NHS reference costs 2021/22 - Day case, Unilateral Major Breast Procedures with CC Score 0-2 , Currency: JA20F |
| To fit the pacemaker | £2,613.80 | Unit cost | NHS reference costs 2021/22 - Day case, Implantation of Dual-Chamber Pacemaker with CC Score 0-2, Currency: EY06E |
| Gastroscopy | £596.98 | Unit cost | NHS reference costs 2021/22 - Day case, Diagnostic Endoscopic Upper Gastrointestinal Tract Procedures, 19 years and over, Currency: FE22Z |
| Removal of scab (surgery + stitches) | £1,323.00 | Unit cost | NHS reference costs 2021/22 - Total HRGs - Major Skin Procedures (Day Case) Currency: JC41Z |
| Blood Test | £4.70 | Unit cost | NHS reference costs 2021/22 (DAPS) Phlebotomy (Currency Code DAPS08) |
| Minor surgery | £1,038.00 | Unit cost | NHS reference costs 2021/22 - Day case, Index tab: DC |
| Glaucoma | 401.4 | unit cost | NHS reference costs 2021/22 - Day case, Minor, Glaucoma or Iris Procedures, Currency: BZ95Z |
| Fracture in hip | £567.00 | Unit cost | NHS reference costs 2021/22 - Total HRGs - Hip Fracture without Interventions, with CC Score 0-3 (Day Case) Currency: HE11H |
| Iron Infusion | £1,038.00 | Unit cost | NHS reference costs 2021/22 - Day case, Index tab: DC |
| Eye Operation | £1,399.42 | Unit cost | NHS reference costs 2021/22 - Day case, Intermediate, Cornea or Sclera Procedures, with CC Score 0, Currency: BZ64B |
| Myeloma - infusion to strengthen bones | £1,038.00 | Unit cost | NHS reference costs 2021/22 - Day case, Index tab: DC |
| Cataract surgery | £1,269.47 | Unit cost | NHS reference costs 2021/22 - Total HRGs - Phacoemulsification Cataract Extraction and Lens Implant, with CC Score 0-1 (Day case) Currency: BZ34C |
| Cystoscopy | £652.00 | Unit Cost | NHS reference costs 2021/22 - Total HRGs -Diagnostic Flexible Cystoscopy 19 years and over (Day case), Currency: LB72A |
| Temporal artery biopsy | £1,747.98 | Unit cost | NHS reference costs 2021/22 - Total HRGs - Biopsy of Temporal Artery (Day case), Currency: YQ43Z |
| Laparoscopy | £2,400.98 | Unit cost | NHS reference costs 2021/22 - Total HRGs - Minor, Laparoscopic or Endoscopic, Upper Genital Tract Procedures (Day case), Currency: MA10Z |
| Clinical Respiratory Assessment | £1,038.00 | Unit cost | NHS reference costs 2021/22 - Day case, Index tab: DC |
| Cataracts Operation | £1,269.47 | unit cost | NHS reference costs 2021/22 - Total HRGs - Phacoemulsification Cataract Extraction and Lens Implant, with CC Score 0-1 (Day case) Currency: BZ34C |
| Cancerous Growth Removal | £1,323.00 | Unit Cost | NHS reference costs 2021/22 - Total HRGs - Major Skin Procedures (Day Case) Currency: JC41Z |
| Eye Procedure | £1,399.42 | Unit cost | NHS reference costs 2021/22 - Day case, Intermediate, Cornea or Sclera Procedures, with CC Score 0 , Currency: BZ64B |
| Back Procedure | £1,038.00 | Unit cost | NHS reference costs 2021/22 - Day case, Index tab: DC |
| **Outpatient:** |  |  |  |
| Urine/stool sample | £3.35 | Unit cost | NHS reference costs 2021/22 (DAPS) Other (Currency Code DAPS09) |
| Blood test | £4.70 | Unit cost | NHS reference costs 2021/22 (DAPS) Phlebotomy (Currency Code DAPS08) |
| Nurse intervention/consultation | £40.00 | Unit cost | NHS reference costs 2021/22 - (Other Currencies - total) Specialist Nursing, Treatment Room Nursing Services, Adult, Face to face (Currency code N27AF) |
| Specialist nurse intervention/consultation | £76.00 | Unit cost | NHS reference costs 2021/22 - Other Currencies, Other Specialist Nursing, Adult, Face to face (N29AF) |
| Paramedic or pharmacist outpatient contact | £53.00 | per hour | Cost per working hour, Band 6 Hospital Health Professional (PSSRU, 2022, p.94). |
| X-ray | £41.41 | Unit cost | NHS reference costs 2021/22 - Imaging Outpatient - Plain Film (IMAGOP: PF) |
| DEXA | £44.46 | Unit Cost | NHS reference costs 2021/22 - Imaging Outpatient - Dexa Scan (IMAGOP: RD50Z) |
| CT scan | £104.97 | Unit Cost | NHS reference costs 2021/22 - Imaging Outpatient - Computerised Tomography Scan of One Area, without Contrast, 19 years and over (IMAGOP: RD20A) |
| MRI scan | £188.11 | Unit Cost | NHS reference costs 2021/22 - Imaging Outpatient - Magnetic Resonance Imaging Scan of One Area, without Contrast, 19 years and over (IMAGOP: RD01A) |
| General diagnostic scan | £46.00 | Unit cost | NHS reference costs 2021/22 - Total Outpatient Attendance - Diagnostic Imaging Service (Total) Service code: 812 |
| Ultrasound scan | £77.81 | Unit cost | NHS reference costs 2021/22 - Imaging Outpatient - Ultrasound Scan with duration of less than 20 minutes, without Contrast (IMAGOP: RD40Z) |
| Echocardiogram | £134.35 | Unit Cost | NHS reference costs 2021/22 - Imaging Outpatient - Simple Echocardiogram, 19 years and over (IMAGOP: RD51A) |
| Electrocardiogram | £181.00 | Unit cost | NHS reference costs 2021/22 - Electrocardiogram Monitoring or Stress Testing (Total HRGs tab, outpatient procedure - EY51Z) |
| Physiotherapy/musculoskeletal problem | £73.00 | Unit cost | NHS reference costs 2021/22 - (Other Currencies - total) Physiotherapist, Adult, One to One (Currency code A08A1) |
| Physiotherapist group session | £66.00 | Unit cost | NHS reference costs 2021/22 - (Other Currencies - total) Physiotherapist, Adult, Group (Currency code A08AG) |
| Podiatrist | £69.00 | Unit cost | NHS reference costs 2021/22 - (Other Currencies - total) Podiatrist, Tier 1, General Podiatry (Currency code A09A) |
| Dietician | £77.00 | Unit cost | NHS reference costs 2021/22 - Other Currencies, Dietitian (A03) |
| Occupational therapist | £99.00 | Unit cost | NHS reference costs 2021/22 - (Other Currencies - total) Occupational Therapist, Adult, One to One (Currency code A06A1) |
| Family planning clinic | £104.00 | Unit cost | NHS reference costs 2021/22 - Family Planning Clinic £104, Total Outpatient Attendance, service code FPC |
| Incontinence service | £108.00 | Unit Cost | NHS reference costs 2021/22 (Other Currencies) Specialist Nursing, Continence Services, Adult, Face to face (Currency code: N14AF) |
| Parkinson’s/ dementia nurse | £118.00 | Unit cost | NHS reference costs 2021/22 - Other Currencies, Specialist Nursing, Parkinson's and Alzheimer’s Nursing/Liaison, Adult, Face to face (N22AF) |
| Midwifery | £120.00 | Unit cost | NHS reference costs 2021/22 - Total Outpatient Attendance - MIDWIFERY SERVICE (Total) Service code: 560 |
| Audiology | £126.00 | Unit cost | NHS reference costs 2021/22 - (Total Outpatient Attendance) - Total - Audiology Service (service code 840) |
| Speech and language therapist | £128.00 | Unit cost | NHS reference costs 2021/22 - (Other Currencies - total) Speech and Language Therapist, Adult, One to One (Currency code A13A1) |
| Colorectal surgery service | £130.00 | Unit cost | NHS reference costs 2021/22 - Total Outpatient Attendance - Colorectal Surgery Service (Total) Service code: 104. |
| Urology service | £138.00 | Unit cost | NHS reference costs 2021/22 - Total Outpatient Attendance -Urology Service (Total) Service code: 101. |
| Optometry service | £138.00 | Unit cost | NHS reference costs 2021/22 - Total - Optometry Service (Service Code 662) 'Total Outpatient Attendance'. |
| Ophthalmology service | £142.00 | Unit cost | NHS reference costs 2021/22 - Ophthalmology Service, Unit costs £142, Total Outpatient Attendance, Service code: 130 |
| Gastroenterology service | £149.00 | unit cost | NHS reference costs 2021/22 - Total Outpatient Attendance - Gastroenterology Service (Total) Service code: 301. |
| Dermatology service | £152.00 | unit cost | NHS reference costs 2021/22 - (Total Outpatient Attendance) Dermatology Service - Total (service code 330) |
| Ear nose and throat service | £155.00 | unit cost | NHS reference costs 2021/22 - Total Outpatient Attendance - Ear Nose throat Service (Total) Service code: 120. |
| Orthopaedic service | £157.00 | unit cost | NHS reference costs 2021/22 - Total Outpatient Attendance - Orthopaedic Service (Total) Service code: 111 |
| Orthopaedic trauma service (including break/fracture) | £159.00 | unit cost | NHS reference costs 2021/22 - Total Outpatient Attendance - Trauma and Orthopaedic Service (Total) Service code: 110 |
| Rheumatology service | £165.00 | unit cost | NHS reference costs 2021/22 - Total Outpatient Attendance - Rheumatology Service (Total) Service code: 410. |
| Cardiology service | £169.00 | unit cost | NHS reference costs 2021/22 - Total Outpatient Attendance -Cardiology Service (Total) Service code: 320. |
| Orthotics service | £172.00 | unit cost | NHS reference costs 2021/22 - Total Outpatient Attendance - Orthotics Service (Total) Service code: 658 |
| Gynaecology service | £181.00 | Unit cost | NHS reference costs 2021/22 - Total Outpatient Attendance -Gynaecology Service (Total) Service code: 502. |
| Diabetes service | £183.00 | Unit cost | NHS reference costs 2021/22 - Total Outpatient Attendance - Diabetes Service - Total (service code 307) |
| Respiratory medicine service | £185.00 | Unit cost | NHS reference costs 2021/22 - Total Outpatient Attendance - Respiratory Medicine Service (Total) Service code: 340. |
| Dental service | £192.00 | Unit cost | NHS reference costs 2021/22 - Other Currencies, General Dental Service, Contact (M01B) |
| Orthodontic service | £211.00 | Unit cost | NHS reference costs 2021/22 - Total Outpatient Attendance - Orthodontic Service (Total) Service code: 143 |
| Tooth extraction | £339.00 | Unit cost | NHS reference costs 2021/22 - Total HRGs - Minor Extraction of Tooth, 19 years and over (Outpatient procedure) Currency: CD07A |
| Haematology service | £194 | Unit cost | NHS reference costs 2021/22 - Total Outpatient Attendance - Clinical Haematology Service (303) |
| Other general outpatient appointment (including prefer not to answer) | £184.00 | Unit cost | NHS reference costs 2021/22 - Consultant Led (Index tab - CL) |
| Other general outpatient procedure | £203.00 | Unit cost | NHS reference costs 2021/22 - Outpatient procedures (Index tab - OPROC) |
| Renal services | £197.00 | Unit cost | NHS reference costs 2021/22 - Total Outpatient Attendance - Renal Medicine Service (Total) Service code: 361. |
| Oncology service | £206.00 | Unit cost | NHS reference costs 2021/22 - Medical oncology service - Total (Service Code 370) 'Total Outpatient Attendance'. |
| Chemotherapy infusion | £440.71 | Unit cost | NHS reference costs 2021/22 - Deliver Complex Chemotherapy, including Prolonged Infusional Treatment, at First Attendance (Index tab - CHEM, Outpatient - OP service Code, Currency Code SB14Z) |
| Chemotherapy | £515.00 | Unit cost | NHS reference costs 2021/22 - Chemotherapy (Index tab - CHEM) |
| Endocrine clinic | £198.00 | Unit cost | NHS reference costs 2021/22 - Total Outpatient Attendance -Endocrinology Service (Total) Service code: 302. |
| Neurology service | £214.00 . | Unit cost | NHS reference costs 2021/22 - Neurology Service - Total (Service Code 400) 'Total Outpatient Attendance'. |
| Minor skin procedure | £198.00 | Unit cost | NHS reference costs 2021/22 - Total HRGs - Minor Skin Procedures, 19 years and over (Outpatient procedure) Currency: JC43C |
| Pacemaker review | £216.00 | Unit cost | NHS reference costs 2021/22 - Total HRGs - Testing of Cardiac Pacemaker or Cardioverter Defibrillator (Outpatient procedure) Currency: EY11Z |
| Glaucoma procedure | £217.82 | Unit cost | NHS reference costs 2021/22 - OPROC (Minor, Glaucoma or Iris Procedures - Currency Code: BZ95Z) |
| Nerve conduction procedure | £229.50 | Unit cost | NHS reference costs 2021/22 - Conventional EEG, EMG or Nerve Conduction Studies, 19 years and over (Currency Code: AA33C, Service Code: 812) DIAGNOSTIC IMAGING SERVICE |
| Diagnosis from psychologist | £253.00 . | Unit cost | NHS reference costs 2021/22 - Clinical Psychology Service - Total (Service Code 656) 'Total Outpatient Attendance' |
| Hysteroscopy | £282.00 | Unit cost | NHS reference costs 2021/22 - Diagnostic Hysteroscopy - Outpatient procedures (Total HRGs tab, currency: MA31Z) |
| TIA clinic | £293.00 | Unit cost | NHS reference costs 2021/22 - Total Outpatient Attendance - Transient Ischaemic Attack Service (Total) Service code: 329 |
| Immunology | £303.00 | Unit cost | NHS reference costs 2021/22 - Total - Clinical Immunology Service (Service Code 316) 'Total Outpatient Attendance' |
| Tongue biopsy | £348.00 | Unit cost | NHS reference costs 2021/22 - Total HRGs - Excision or Biopsy, of Lesion of Mouth, 19 years and over (Outpatient procedure) Currency: CA66A |
| Sleep clinic | £355.00 | Unit cost | NHS reference costs 2021/22 - Total Outpatient Attendance - Sleep Medicine Service (Total) Service code: 347 |
| Elderly medicine | £372.00 | Unit cost | NHS reference costs 2021/22 - Total - Elderly Medicine Service (Service Code 430) 'Total Outpatient Attendance'. |
| Sigmoidoscopy | £510.00 | Unit cost | NHS reference costs 2021/22 - Diagnostic Flexible Sigmoidoscopy, 19 years and over - Outpatient procedures (Total HRGs tab, currency: FE35Z) |
| Colonoscopy | £510.00 | Unit cost | NHS reference costs 2021/22 - Diagnostic Colonoscopy, 19 years and over (Total HRGs tab, Outpatient procedures, currency: FE32Z) |
| Gastroscopy and endoscopy | £540.00 | Unit cost | NHS reference costs 2021/22 - Total HRGs - Diagnostic Endoscopic Upper Gastrointestinal Tract Procedures, 19 years and over (Outpatient procedure) Currency: FE22Z |
| Oesophageal Manometry test | £575.00 | Unit cost | NHS reference costs 2021/22 - Major, Oesophageal, Stomach or Duodenum Procedures, 19 years and over, with CC Score 0-1, FF04D, Unit cost £575 outpatient procedure, Total HRGs |
| Cataract surgery | £1,268.00 | Unit Cost | NHS reference costs 2021/22 - Total HRGs - Phacoemulsification Cataract Extraction and Lens Implant, with CC Score 0-1 (Total) Currency: BZ34C |
| Skin cancer removal | £1,323.00 | Unit cost | NHS reference costs 2021/22 - Total HRGs - Major Skin Procedures (Day Case) Currency: JC41Z |
| Surgery on hand | £1,484.00 | Unit cost | NHS reference costs 2021/22 - Minor Hand Procedures for Non-Trauma, 19 years and over (Total HRGs tab, Day case, currency: HN45A) |
| Surgery on foot | £1,842.00 | Unit cost | NHS reference costs 2021/22 - Minor Foot Procedures for Non-Trauma, 19 years and over (Total HRGs tab, Day case, currency: HN35A) |
| Shoulder surgery | £2,200.00 | Unit cost | NHS reference costs 2021/22 - Minor Shoulder Procedures for Non-Trauma (Total HRGs tab, Day case, currency: HN55Z) |
| **Other community-based resource use:** | **Unit cost** | **Unit:** | **Source:** |
| Covid-19 vaccination | £10.06 | per intervention | NHS England. COVID-19 vaccination programme: 1 September 2022 to 31 August 2023 (section 11.1.1)  <https://www.england.nhs.uk/coronavirus/documents/enhanced-service-specification/#service-delivery> |
| Flu vaccination | £10.06 | per intervention | NHS England. COVID-19 vaccination programme: 1 September 2022 to 31 August 2023 (section 11.1.1)  <https://www.england.nhs.uk/coronavirus/documents/enhanced-service-specification/#service-delivery> |
| Phlebotomy | £4.70 | unit cost | NHS reference costs 2021/22 (DAPS) Phlebotomy (Currency Code DAPS08) |
| Clinician from GP practice (not GP) clinic visit | £52.00 | per hour | Cost as practice nurse Band 5. Includes qualification costs (PSSRU, 2022, p.68). |
| GP paramedic clinic visit | £55.00 | per hour | Cost per working hour, Band 6 Community Health Professional (PSSRU, 2022, p.60). |
| GP pharmacist clinic visit | £55.00 | per hour | Costed as a Band 6 Community-based Pharmacist (PSSRU, 2022, p.60). |
| GP pharmacist telephone | £13.75 | Per contact | Assumed 15 minutes. Costed as a Band 6 Community-based Pharmacist (PSSRU, 2022, p.60). |
| Physician assistant clinic visit | £66.00 | per hour | Cost per working hour, Band 7 Community Health Professional (PSSRU, 2022, p.60). |
| Physician assistant telephone | £16.50 | per hour | Assumed 15 minutes. Cost per working hour, Band 7 Community Health Professional (PSSRU, 2022, p.60). |
| Breast screening service | £52.45 | per intervention | No community-based cost located in PSSRU, costed as hospital service. Diagnostic imaging service (service code 812), Non-Admitted Face-to-Face Attendance, First (NHS reference costs, 2021/2022). |
| Chiropodist | £42.00 | per hour | Costed as a Band 5 Community-based Podiatrist (PSSRU, 2022, p.60). |
| District/Admiral/Macmillan/Hospice nurse clinic visit | £63.00 | per hour | Unit cost per working hour (with qualifications) Band 6 community-based nurse (PSSRU, 2022, p.66). |
| District/Admiral/Macmillan/Hospice nurse home visit | £63.00 | per hour | Assumed same as clinic visit. Unit cost per working hour (with qualifications) Band 6 community-based nurse (PSSRU, 2022, p.66). |
| District/Admiral/Macmillan/Hospice nurse telephone | £8.69 | per intervention | Per intervention lasting 6.56 minutes. Band 5 nurse. Includes other costs (PSSRU, 2022, p.73). |
| Healthcare assistant | £25.00 | per hour | Costed as Support and outreach worker (p.86) which has a similar salary £20181 (healthcare assistants and other support staff from table 12.2.1 (PSSRU, 2022, p107) £20,148), but includes other costs. |
| Community physiotherapist/rehab team | £42.00 | per hour | Cost per working hour, Band 5 Community Physiotherapist (PSSRU, 2022, p.60). |
| Cognitive simulation therapy support worker | £42 | Per hour | Cost per working hour, Band 5 Community occupational therapist, Counsellor, clinical psychology trainee (PSSRU, 2022, p.60). |
| Adult social services/social worker | £50.00 | per hour | Social worker (adult services) - with qualifications, Table 10.1 (PSSRU, 2022, p.81). |
| Social worker telephone | £12.50 | Per contact | Assumed 15 minutes per telephone contact. £50 per hour social worker (PSSRU 2022, p.81). |
| Optician | £43.01 | per intervention | NHS Primary eye care - Cost weighted by maximum number of sight tests (Optometric Fees Negotiating Committee, 2022, p.5). |
| Gym GP exercise referral class | £17.94 | per week | Costed as National Exercise Referral Scheme 16/17 LA costs = £244 (Hawkins et al., 2019, p.11). Bank of England inflation calculator for 2022 = £287 / 16 weeks (length of program) = £17.94 per week. |
| Social prescriber | £37.00 | per hour | Costed as a Band 4 Community based practitioner (PSSRU, 2022, p.60). |
| Meals on wheels | £8.50 | per meal | Meals on Wheels, average cost £6.60 per meal (LA), £46 per week. (PSSRU, 2014, p.127) Bank of England inflation calculator £8.50 per meal, £56 per week. |
| Community support worker | £25.00 | per hour | Support and outreach worker - cost per hour (PSSRU, 2022, p.86). |
| Day care at another location | £78.00 | per attendance | Costed as local authority own-provision day care for older people (age 65+), £78 per client attendance (PSSRU, 2022, p.13) |
| Sitting service at home | £23.00 | per weekday hour | Costed as Home Care worker (PSSRU, 2022, p.84) |
| Overnight care attendant at home | £23.00 | per weekday hour | Costed as Home Care worker (PSSRU, 2022, p.84) |
| Respite care at another location | £23.00 | per weekday hour | Costed as Home Care worker (PSSRU, 2022, p.84) |
| Carer | £23.00 | per weekday hour | Costed as Home Care worker (PSSRU, 2022, p.84) |

| **Inpatient stays (NHS England. Annex A 2021/22 national tariff workbook 2021/22).** | **Spell cost** | **Excess bed day cost** | **Trim point** | **HRG code** |
| --- | --- | --- | --- | --- |
| Annex A NHS reference costs 2021/22 - APC & OPROC - Unspecified Acute Lower Respiratory Infection without Interventions, with CC Score 0-4 (NE) | £2,250.00 | £238.00 | 14 | DZ23N |
| Annex A NHS reference costs 2021/22 - APC & OPROC - Hypertension (NEL) | £853.00 | £273.00 | 5 | EB04Z |
| Annex A NHS reference costs 2021/22 - APC & OPROC - Diagnostic Flexible Cystoscopy using Photodynamic Fluorescence (EL) | £1,084.00 | £248.00 | 5 | LB73Z |
| Annex A NHS reference costs 2021/22 - APC & OPROC - Tendency to Fall, Senility or Other Conditions Affecting Cognitive Functions, without Interventions, with CC Score 0-1 (NE) | £2,312.00 | £247.00 | 13 | WH09G |
| Annex A NHS reference costs 2021/22 - APC & OPROC - Intermediate Foot Procedures for Non-Trauma, 19 years and over, with CC Score 0-1 (EL) | £2,247.00 | £268.00 | 5 | HN34C |
| Annex A NHS reference costs 2021/22 - APC & OPROC - Asthma with Interventions (NEL) | £3,301.00 | £238.00 | 28 | DZ15M |
| Annex A NHS reference costs 2021/22 - APC & OPROC - Unspecified Chest Pain with CC Score 0-4 (NE) | £386.00 | £273.00 | 5 | EB12C |
| Annex A NHS reference costs 2021/22 - APC & OPROC - Infections or Other Complications of Procedures, without Interventions, with CC Score 0-1 (NEL) | £1,284.00 | £247.00 | 5 | WH07G |
| Annex A NHS reference costs 2021/22 - APC & OPROC - Intermediate Extradural Spinal Procedures with CC Score 0-1 (EL) | £3,396.00 | £268.00 | 5 | HC64C |
| Annex A NHS reference costs 2021/22 - APC & OPROC - Pulmonary Embolus with Interventions, with CC Score 0-8 (NEL) | £5,154.00 | £238.00 | 30 | DZ09K |
| Annex A NHS reference costs 2021/22 - APC & OPROC - Gastrointestinal Infections with Single Intervention, with CC Score 0-1 (NE) | £2,622.00 | £269.00 | 13 | FD01E |
| Annex A NHS reference costs 2021/22 - APC & OPROC - Diagnostic Colonoscopy, 19 years and over (EL) | £478.00 | £269.00 | 5 | FE32Z |
| Annex A NHS reference costs 2021/22 - APC & OPROC -Actual or Suspected Myocardial Infarction, with CC Score 0-3 (NEL) | £1,576.00 | £273.00 | 9 | EB10E |
| Annex A NHS reference costs 2021/22 - APC & OPROC - Major, Open or Laparoscopic, Bladder Neck Procedures (Female) (EL) | £3,039.00 | £248.00 | 6 | LB59Z |
| Annex A NHS reference costs 2021/22 - APC & OPROC - Diagnostic Colonoscopy, 19 years and over (EL) | £478.00 | £269.00 | 5 | FE32Z |
| Annex A NHS reference costs 2021/22 - APC & OPROC - Very Major Hip Procedures for Non-Trauma with CC Score 2-3 (EL) - BPT: Primary Total Hip and Knee Replacements (for double hip replacement) | £6,992.00 | £268.00 | 8 | HN12E |
| Annex A NHS reference costs 2021/22 - APC & OPROC - Major Therapeutic Endoscopic, Upper or Lower Gastrointestinal Tract Procedures, 19 years and over, with CC Score 0 (EL) | £660.00 | £269.00 | 5 | FE02C |
| Annex A NHS reference costs 2021/22 - APC & OPROC - Ureteric or Bladder Disorders, without Interventions, with CC Score 0-1 (NE) | £579.00 | £248.00 | 5 | LB19G |
| Annex A NHS reference costs 2021/22 - APC & OPROC - Gastrointestinal Infections without Interventions, with CC Score 0-1 (EL) | £418.00 | £269.00 | 5 | FD01J |
| Annex A NHS reference costs 2021/22 - APC & OPROC - Inflammatory Bowel Disease without Interventions, with CC Score 0 (NEL) | £1,800.00 | £269.00 | 5 | FD02H |
| Annex A NHS reference costs 2021/22 - APC & OPROC - Unspecified Acute Lower Respiratory Infection without Interventions, with CC Score 0-4 (NE) | £1,070.00 | £238.00 | 5 | DZ22Q |
| Annex A NHS reference costs 2021/22 - APC & OPROC - Complex Echocardiogram (EL) | £478.00 | £273.00 | 5 | EY50Z |
| Annex A NHS reference costs 2021/22 - APC & OPROC - Sepsis with Single Intervention, with CC Score 0-4 (NEL) - *sepsis reported in data* | £4,197.00 | £247.00 | 28 | WJ06F |
| Annex A NHS reference costs 2021/22 - APC & OPROC - Non-Malignant, Hepatobiliary or Pancreatic Disorders, without Interventions, with CC Score 0-1 (NEL) | £1,659.00 | £267.00 | 9 | GC17K |
| Annex A NHS reference costs 2021/22 - APC & OPROC - Percutaneous Transluminal Angioplasty, including Stenting, of Intracranial or Extracranial Blood Vessel (EL) | £2,733.00 | £276.00 | 8 | YA10Z |
| Annex A NHS reference costs 2021/22 - APC & OPROC - Open Operations, on Other or Unspecified Blood Vessels, with CC Score 0-1 (EL) | £1,218.00 | £276.00 | 5 | YQ41B |
| Annex A NHS reference costs 2021/22 - APC & OPROC - Sepsis without Interventions, with CC Score 0-4 (NEL) - *suspected sepsis in data* | £2,483.00 | £247.00 | 15 | WJ06J |
| Annex A NHS reference costs 2021/22 - APC & OPROC - Intermediate Skin Procedures, 13 years and over (EL) | £966.00 | £262.00 | 5 | JC42A |
| Annex A NHS reference costs 2021/22 - APC & OPROC - Bronchopneumonia without Interventions, with CC Score 0-5 (NEL) | £2,250.00 | £238.00 | 14 | DZ23N |
| Annex A NHS reference costs 2021/22 - APC & OPROC - Major Skin Procedures (EL) | £2,187.00 | £262.00 | 5 | JC41Z |
| Annex A NHS reference costs 2021/22 - APC & OPROC - Skin Disorders without Interventions, with CC Score 0-1 (NES) | £637.00 | £262.00 | 5 | JD07K |
| Annex A NHS reference costs 2021/22 - APC & OPROC - Muscular, Balance, Cranial or Peripheral Nerve Disorders, Epilepsy or Head Injury, with CC Score 0-2 (NEL) | £650.00 | £266.00 | 5 | AA26H |
| Annex A NHS reference costs 2021/22 - APC & OPROC - Intermediate Knee Procedures for Non-Trauma, 19 years and over, with CC Score 0-1 (EL) | £1,874.00 | £268.00 | 5 | HN24C |
| Annex A NHS reference costs 2021/22 - APC & OPROC - Stroke with CC Score 0-3 (NEL) | £2,074.00 | £266.00 | 14 | AA35F |
| Annex A NHS reference costs 2021/22 - APC & OPROC - Very Major Hip Procedures for Non-Trauma with CC Score 0-1 (EL) - BPT: Primary Total Hip and Knee Replacements | £6,339.00 | £268.00 | 7 | HN12F |
| Annex A NHS reference costs 2021/22 - APC & OPROC - Very Major Knee Procedures for Non-Trauma with CC Score 0-1 (EL) - BPT: Primary Total Hip and Knee Replacements | £6,314.00 | £268.00 | 7 | HN22E |
| Annex A NHS reference costs 2021/22 - APC & OPROC - Excision or Biopsy, of Lesion of External Nose (EL) | £528.00 | £280.00 | 5 | CA16Z |
| Annex A NHS reference costs 2021/22 - APC & OPROC - Sepsis with Multiple Interventions, with CC Score 0-4 (NEL) - *Treatment for sepsis, heart failure and compacted bowel reported in data* | £6,712.00 | £247.00 | 42 | WJ06C |
| Annex A NHS reference costs 2021/22 - APC & OPROC - Hip Fracture without Interventions, with CC Score 0-3 (NE) | £2,237.00 | £268.00 | 30 | HE11H |
| Annex A NHS reference costs 2021/22 - APC & OPROC - Rib or Chest Fracture, without Interventions, with CC Score 0-2 (NE) | £671.00 | £268.00 | 8 | HE71D |
| Annex A NHS reference costs 2021/22 - APC & OPROC - Very Major Hip Procedures for Non-Trauma with CC Score 0-1 (NE) - BPT: Primary Total Hip and Knee Replacements | £6,770.00 | £268.00 | 31 | HN12F |
| Annex A NHS reference costs 2021/22 - APC & OPROC - Foot Fracture without Interventions, with CC Score 0-1 (NE) | £671.00 | £268.00 | 5 | HE31G |

In addition to the resource use information gathered for carers, additional resource use items for ‘other services’ were collected for patients living with dementia including: day care at another location, sitting service at home, overnight care attendance at home, respite care at another location. For ‘other services’ where respondents were asked to report on the total number of ‘other services’ received by patients living with dementia but had selected multiple providers (i.e. NHS, local authority, third sector, private), without indicating the number of services received from each provider separately, the total number of services were costed from the public sector perspective as we did not have sufficient information to ascertain any subsidised rates received by families to contribute to any services where funding was shared across multiple providers.

**Supplementary Table 3:** **Unit cost schedule – wider perspective**

| **Resource use:** | **Unit cost** | **Unit** | **Source** |
| --- | --- | --- | --- |
| Community support worker (private and third sector) | £25.00 | per hour | Support and outreach worker - cost per hour (PSSRU, 2022, p.86). |
| Day care at another location (private and third sector) | £78.00 | per attendance | Costed as local authority own-provision day care for older people (age 65+), £78 per client attendance (PSSRU, 2022, p.13) |
| Sitting service at home (private and third sector) | £23.00 | per weekday hour | Costed as Home Care worker (PSSRU, 2022, p.84) |
| Overnight care attendant at home (private and third sector) | £23.00 | per weekday hour | Costed as Home Care worker (PSSRU, 2022, p.84) |
| Respite care at another location (private and third sector) | £23.00 | per weekday hour | Costed as Home Care worker (PSSRU, 2022, p.84) |
| Carer (private and third sector) | £23.00 | per weekday hour | Costed as Home Care worker (PSSRU, 2022, p.84) |
| Privately funded emergency alarm bracelet | £68.00 | per year | £68 per annum (Careline website) |
| Privately funded GP/geriatrician clinic visit | £250.00 | per hour | Per 60-minute appointment (Bupa) |
| Privately funded GP/geriatrician telephone | £59.00 | Per contact | Per 15-minute telephone/video conference appointment (Bupa) |
| Privately funded physiotherapist | £72/£49 | Per initial consultation/per follow-up consultation | Outside greater London cost (Bupa). |
| Privately funded life coach | £60 | Per hour | Costed at upper end of 'beginner' (indeed.com) |
| Privately funded chiropractor | £75.00 | per session | Mid-range cost for first session (between £50 and £100) (NHS) |
| Privately funded osteopath | £55.89 | per session | Initial fee national average (iosteopathy website) |
| Third sector contacts/support (e.g., Age UK, Alzheimer’s Society, carers centre, befriending service, support groups etc.) | £25.00 | per hour | Costed as a Support and outreach worker which includes 'costs' (PSSRU, 2022, p.86) |
| Third sector contacts/support telephone | £6.25 | Per contact | Assumed 15 minutes £25.00/4 = £6.25. Costed as a Support and outreach worker which includes 'costs' (PSSRU, 2022, p.86) |

Miscellaneous services reported but not included in our analysis included fire service, police, Scottish Brain Science movement (research participant) and employee wellbeing service.

**References of unit cost sources**

**PSSRU**

- Jones KC, Weatherly H, Birch S, Castelli A, Chalkley M, Dargan A, Forder JE, Gao J, Hinde S, Markham S, Ogunleye D. Unit costs of health and social care 2022 manual. Personal Social Services Research Unit.
- Jones KC, Burns A. Unit costs of health and social care 2021. Personal Social Services Research Unit.
- Curtis, L.A. 2014. Unit costs of health and social care 2014. Personal Social Services Research Unit.

**NHS published reference costs**

- NHS England. National Cost Collection for the NHS. 2021/22. https://www.england.nhs.uk/publication/2021-22-national-cost-collection-data-publication/
- NHS England. Annex A 2021/22 national tariff workbook 2021/22. https://www.england.nhs.uk/publication/national-tariff-payment-system-documents-annexes-and-supporting-documents/

**Wider sources**

- Optometric Fees Negotiating Committee, 2022. See page 5 of letter. <https://www.aop.org.uk/-/media/files/media-centre/ofnc-fees-and-grants-28-09-22.pdf?rev=957ed160fee24840a1c4aa7d2327db41&hash=A4B3F540DF126C3BD8C6A94CF6B757AD>
- NHS England. COVID-19 vaccination programme: 1 September 2022 to 31 August 2023 (section 11.1.1) https://www.england.nhs.uk/coronavirus/documents/enhanced-service-specification/#service-delivery
- Hawkins J, Charles JM, Edwards M, Hallingberg B, McConnon L, Edwards RT, Jago R, Kelson M, Morgan K, Murphy S, Oliver EJ. Acceptability and feasibility of implementing accelorometry-based activity monitors and a linked web portal in an exercise referral scheme: feasibility randomized controlled trial. Journal of medical Internet research. 2019 Mar 29;21(3):e12374.
- Careline. Emergency care bracelet. <https://www.careline.co.uk/>
- Bupa. Private GP clinic visit. <https://www.bupa.co.uk/health/payg/gp-services?pdg_channel=ppc&pdg_account=gp&pdg_network=GOOGLE&pdg_campaign=GP+%7C+F2F+%7C+Brand&pdg_adgroup=F2F+%7C+Brand&pdg_keyword=bupa+gp+services&gad_source=1&gclid=EAIaIQobChMImpKaqZ2thAMVsY9QBh2KWQqNEAAYASAAEgKLKfD_BwE&gclsrc=aw.ds>
- Bupa Private GP telephone call. <https://www.bupa.co.uk/health/payg/gp-services?pdg_channel=ppc&pdg_account=gp&pdg_network=GOOGLE&pdg_campaign=GP+%7C+F2F+%7C+Brand&pdg_adgroup=F2F+%7C+Brand&pdg_keyword=bupa+gp+services&gad_source=1&gclid=EAIaIQobChMImpKaqZ2thAMVsY9QBh2KWQqNEAAYASAAEgKLKfD_BwE&gclsrc=aw.ds>
- Bupa. Private physiotherapist. <https://www.bupa.co.uk/health/payg/physiotherapy#prices>
- Indeed. Private life coach. <https://uk.indeed.com/career-advice/pay-salary/how-much-does-life-coach-make>
- Iosteopath. Private osteopath. <https://www.iosteopathy.org/wp-content/uploads/2022/09/iO-Professional-Census-June-2022.pdf>
- NHS. Private chiropractor. <https://www.nhs.uk/conditions/chiropractic/>

**Informal care approach: opportunity cost method**

We also conducted a separate sensitivity analysis using the opportunity cost method to explore variations in our costing assumptions to calculate informal care costs. At baseline, 3-month and 6-month follow-up, care participants were asked to report on the number hours spent during the last week on household activities, personal care and practical support. Opportunity costs of informal care time were calculated based on salary information obtained from the UK Government website, the PSSRU (Jones et al., 2022), and other online job market websites (where job salary information was not available for some occupations reported in the data) to source to cost an hourly rate for informal care tasks based on the occupational role details provided by participants. An hourly rate was calculated using the mid-point salary assuming 37.5 working hours per week, and 52 weeks per year. For participants of working age who were not in employment during the time of the study, lost income was valued at the UK national living wage (£8.91 per hour for people aged 23 and over between April 2021 and March 2022) (UK Government, 2023). For retired participants, opportunity costs were valued as a proxy cost of lost leisure time valued at £14.82 per hour in the cost year 2021/2022, converted and inflated from 16 Euros per hour in 2014 from published estimates (Verbooy et al., 2018).

Sources of costs for opportunity cost method:

- Glass Door - <https://www.glassdoor.co.uk>
- Jones KC, Weatherly H, Birch S, Castelli A, Chalkley M, Dargan A, Forder JE, Gao J, Hinde S, Markham S, Ogunleye D. Unit costs of health and social care 2022 manual. Personal Social Services Research Unit.
- UK Government. National Careers Service. <https://nationalcareers.service.gov.uk/explore-careers>
- UK Government. National Minimum Wage Rates. <https://www.gov.uk/national-minimum-wage-rates>
- Verbooy K, Hoefman R, Van Exel J, Brouwer W. Time is money: investigating the value of leisure time and unpaid work. Value in Health. 2018 Dec 1;21(12):1428-36.

**Supplementary Table 4: Baseline characteristics of complete cases EQ-5D-5L participants (n=261)**

| Control group (n=136) | | **Control group n (%)** | **Intervention group n (%)** |
| --- | --- | --- | --- |
| Intervention group (n=125) | |  |  |
| **Age (mean, ±SD)** | | 63 (10.64) | 64 (10.70) |
| **Female** |  | 104 (76%) | 97 (78%) |
| **Main language (English)** | | 134 (99%) | 123 (98%) |
| **Ethnicity** |  |  |  |
|  | English/Welsh/Scottish/Northern Irish/British | 128 (94%) | 120 (96%) |
| **Highest education** | |  |  |
|  | University higher degree | 36 (27%) | 31 (25%) |
|  | First degree level qualification | 35 (26%) | 41 (33%) |
|  | Degree level | 23 (17%) | 16 (13%) |
|  | A level | 10 (7%) | 10 (8%) |
|  | NVQ level 3 or below | 2 (2%) | 5 (4%) |
|  | CSEs, GCSE/O level | 17 (13%) | 12 (10%) |
| **Occupational status** | |  |  |
|  | Professional | 81 (60%) | 70 (57%) |
|  | Managerial/technical | 28 (21%) | 25 (20%) |
|  | Skilled (non-manual) | 21 (16%) | 24 (19%) |
|  | Skilled (manual) | 4 (3%) | 2 (2%) |
|  | Partly skilled | 0 (0%) | 1 (1%) |
|  | Unskilled | 0 (0%) | 2 (2%) |
| **Have paid work** |  |  |  |
|  | Yes Part-time | 22 (16%) | 23 (18%) |
|  | Fulltime | 22 (16%) | 23 (18%) |
|  | No Retired | 81 (60%) | 72 (57%) |
| **Marital status** |  |  |  |
|  | Married | 104 (77%) | 95 (76%) |
|  | Single and never married or never in a legally recognised Civil Partnership | 70 (7%) | 10 (8%) |
|  | Civil Partnership in a legally recognised Civil Partnership | 6 (4%) | 2 (2%) |
|  | Cohabiting | 8 (6%) | 9 (7%) |
|  | Separate but legally married | 0 (0%) | 1 (1%) |
|  | Divorced | 4 (3%) | 7 (6%) |
|  | Windowed | 4 (3%) | 1 (1%) |
| **Relationship with person with dementia** | |  |  |
|  | Spouse/partner | 62 (46%) | 55 (44%) |
|  | Sibling | 2 (2%) | 1 (1%) |
|  | Child | 65 (48%) | 62 (50%) |
|  | Friend | 1 (1%) | 1 (1%) |
|  | Other | 6 (4%) | 6 (5%) |
| **Type of dementia** | |  |  |
|  | Alzheimer's disease | 60 (44%) | 56 (45%) |
|  | Vascular dementia | 21 (15%) | 19 (15%) |
| **Travel distance from person with dementia (minutes, SD)** | | 27 (85.04) | 30 (82.12) |
| **Experience in providing informal care (years, SD)** | | 2.9 (0.23) | 2.9 (0.30) |

**Supplementary Table 5: Frequencies of carer resource use (public sector perspective)**

| **Parameter (total visits)** | **Control group** | | | **Intervention group** | | |
| --- | --- | --- | --- | --- | --- | --- |
|  | **Baseline** | **3 months** | **6 months** | **Baseline** | **3 months** | **6 months** |
| **Community based services** |  |  |  |  |  |  |
| GP | 149 | 119 | 131 | 184 | 135 | 109 |
| Nurse | 65 | 52 | 64 | 91 | 51 | 56 |
| Mental Health Team Worker | 13 | 7 | 10 | 11 | 18 | 4 |
| Psychiatrist | 0 | 0 | 0 | 3 | 0 | 0 |
| Psychologist | 0 | 13 | 13 | 3 | 21 | 4 |
| Counsellor | 28 | 16 | 56 | 31 | 30 | 33 |
| Social worker | 13 | 8 | 24 | 11 | 9 | 12 |
| Occupational therapist | 5 | 2 | 0 | 4 | 5 | 4 |
| Support/education group | 90 | 133 | 173 | 142 | 174 | 138 |
| Other services | 51 | 38 | 47 | 46 | 27 | 28 |
| **Hospital services** |  |  |  |  |  |  |
| Accident and Emergency | 5 | 3 | 4 | 3 | 4 | 7 |
| Rapid/ Medical/ Surgical Assessment Unit | 1 | 2 | 1 | 6 | 8 | 4 |
| Inpatient admission (number of nights) | 4 | 8 | 12 | 64 | 16 | 10 |
| Day hospital | 8 | 4 | 1 | 6 | 4 | 0 |
| Memory clinic | 1 | 0 | 0 | 2 | 3 | 0 |
| Outpatient attendances | 83 | 65 | 81 | 98 | 53 | 77 |
| Other services | 2 | 3 | 6 | 6 | 7 | 6 |

**Supplementary Table 6: Subgroup analysis on the total time spent on the iSupport platform from NHS perspective (mean total costs of health service use of intervention group (SD))**

| **Subgroup categories** | **n (%)** | **Baseline** | **3 months** | **6 months** | **Difference in total mean costs between baseline and 6 months** |
| --- | --- | --- | --- | --- | --- |
| **0 hr** | 32 (18%) | £199 (270) | £684 (1050) | £676 (1271) | £395 (1332) |
| **<1 hr** | 69 (39%) | £342 (1644) | £294 (835) | £321 (669) | £160 (655) |
| **1-2 hrs** | 38 (22%) | £230 (339) | £229 (325) | £210 (336) | -£31 (475) |
| **2-3 hrs** | 16 (9%) | £365 (759) | £428 (941) | £180 (348) | -£209 (835) |
| **≥ 3 hrs** | 20 (11%) | £202 (322) | £262 (444) | £209.62 (405) | £4 (456) |

**Supplementary Table 7: Total resource use costs for patients living with dementia from the public sector and wider societal perspective**

| **Cost details:** | **Control group** | | | | | | **Intervention group** | | | | | |
| --- | --- | --- | --- | --- | --- | --- | --- | --- | --- | --- | --- | --- |
|  | **n** | **Baseline** | **n** | **3 months** | **n** | **6 months** | **n** | **Baseline** | **n** | **3 months** | **n** | **6 months** |
| Total resource use costs (public sector perspective) | 175 | £131,680 | 138 | £112,902 | 130 | £119,716 | 175 | £127,115 | 130 | £88,072 | 122 | £108,994 |
| Total private costs (societal perspective) |  | £6,772 |  | £10,533 |  | £14,685 |  | £20,772 |  | £26,852 |  | £21,744 |
| Total third sector costs (with other services) |  | £8,009 |  | £2,826 |  | £5,872 |  | £3,172 |  | £3,184 |  | £3,315 |
| **Total resource use costs: public sector, private and third sector (societal perspective)** |  | **£146,461** |  | **£126,261** |  | **£140,273** |  | **£151,059** |  | **£118,108** |  | **£134,053** |

**Supplementary Table 8. Mean costs of hospital, community and other services for people living with dementia from the public sector perspective (£, 95% CI)**

|  | **Control group** | | | | **Intervention group** | | | | **Mean change at 6 months between groups (Intervention - control)** | **p value** |
| --- | --- | --- | --- | --- | --- | --- | --- | --- | --- | --- |
|  | **Baseline (n=177)** | **3 months (n=138)** | **6 months (n=130)** | **Mean cost difference between baseline and 6 months** | **Baseline (n=175)** | **3 months (n=129)** | **6 months (n=122)** | **Mean cost difference between baseline and 6 months** |  |  |
| **Mean cost of community services (public sector)** | £224 (188,261) | £192 (157,230) | £205 (165,247) | -£20 (-69,29) | £246 (208,286) | £226 (181,280) | £240 (188,295) | £2 (-44,54) | £22 (-44,90) | 0.55 |
| **Mean cost of hospital services (public sector)** | £446 (302,617) | £562 (307,898) | £614 (349,927) | £143 (-171,501) | £368 (249,513) | £388 (228,591) | £577 (345,837) | £161 (-161,491) | £18 (-485,499) | 0.74 |
| **Mean cost of other services (public sector)** | £81 (26,153) | £64 (17,128) | £102 (32-190) | £24 (-61,102) | £114 (52,184) | £74 (16,149) | £76 (27,138) | -£11 (-101,69) | £35 (-78,148) | 0.59 |
| **Total mean resource use: community, hospital, and other services (public sector)** | **£744 (567,945)** | **£818 (548,1171)** | **£921 (646-1234)** | **£147 (-159,463)** | **£726 (581,899)** | **£683 (502,897)** | **£893 (627,1186)** | **£159 (-191,527)** | **£12 (-471,447)** | 0.48 |

For 95% CI using non-parametric bootstrapping 5,000 samples bias-corrected and accelerated method. *Due to non-normality of cost data, Mann–Whitney U independent t test was used with p-value significant level at 0.05.

**Supplementary Table 9: Total and mean (SD) informal care time reported by carers (hours)**

|  | **Control** | | | | | | **Intervention** | | | | | |
| --- | --- | --- | --- | --- | --- | --- | --- | --- | --- | --- | --- | --- |
|  | **Total hours** | | | **Mean hours** | | | **Total hours** | | | **Mean hours** | | |
|  | **Baseline** | **3 months** | **6 months** | **Baseline** | **3 months** | **6 months** | **Baseline** | **3 months** | **6 months** | **Baseline** | **3 months** | **6 months** |
| Household activities | 2,277.00 | 1,691.00 | 1,697.30 | 13.39 (13.41) | 12.34 (12.44) | 13.26 (15.70) | 2,625.50 | 1,588.30 | 1,841.50 | 15.09 (18.22) | 12.61 (13.40) | 15.22 (15.94) |
| Personal care | 1,165.50 | 826.30 | 717.20 | 6.82 (11.09) | 6.25 (7.92) | 5.65 (7.62) | 1,256.80 | 695.30 | 747.50 | 7.18 (11.18) | 6.25 (7.89) | 5.65 (8.67) |
| Practical support | 1,523.50 | 11,095.00 | 1,037.00 | 8.86 (8.93) | 8.04 (7.76) | 8.10 (7.53) | 1,407.00 | 1,025.50 | 1,131.00 | 8.04 (8.78) | 8.01 (9.27) | 8.10 (9.48) |
| **Total informal care time for tasks** | 4,966.00 | 3,662.80 | 3,451.50 | 28.87 (25.49) | 26.54 (22.13) | 26.96 (24.69) | 5,289.30 | 3,309.10 | 3,720.00 | 30.22 (29.89) | 25.85 (25.30) | 30.24 (29.59) |

**Supplementary Table 10:** **Total group costs of resource use and informal care for carers from the wider societal perspective incorporating the opportunity cost method.**

|  |  | **Control group** | | | | |  | **Intervention group** | | | | |
| --- | --- | --- | --- | --- | --- | --- | --- | --- | --- | --- | --- | --- |
|  | **n** | **Baseline** | n | **3 months** | **n** | **6 months** | **n** | **Baseline** | **n** | **3 months** | **n** | **6 months** |
| Total public sector resource use costs | 177 | £29,516 | 140 | £35,737 | 137 | £41,371 | 175 | £48,632 | 137 | £35,079 | 126 | £23,385 |
| Total privately funded resource use costs | 177 | £0 | 140 | £0 | 137 | £72 | 175 | £980 | 137 | £0 | 126 | £785 |
| Total third sector resource use costs | 177 | £350 | 140 | £681 | 137 | £1,644 | 175 | £594 | 137 | £494 | 126 | £75 |
| Total informal care costs (opportunity cost method) | 172 | £75,919 | 138 | £53,415 | 127 | £50,389 | 174 | £82,427 | 128 | £51,430 | 122 | £57,957 |
| **Total costs** | £105,784 | | £89,833 | | £93,476 | | £132,632 | | £87,003 | | £82,202 | |
| Total resource use costs difference between baseline and 3 months |  | -£15,951 |  |  |  |  |  | -£45,629 |  |  |  |  |
| Total resource use costs difference between baseline and 6 months |  | -£12,308 |  |  |  |  |  | -£50,430 |  |  |  |  |

For the intervention group, total resource use costs from the wider perspective including public sector, private, third sector and opportunity costs at baseline was £132,632 (Supplementary Table 4). Total resource use costs from this wider perspective of analysis, decreased to £87,003 at 3-month follow-up and then decreased again to £82,202 at 6-months (Supplementary Table 4). For the control group, total resource use costs from the wider perspective at baseline was £105,748, and decreased to £89,833 at 3-month follow-up, and then increased to £93,476 at 6-months (Supplementary Table 4). From baseline to 6-month follow-up, total resource use costs from the wider societal perspective decreased by £50,430 in the intervention group and by £12,308 in the control group.

**Supplementary Table 11: Difference in mean costs per participant of resource use and informal care for carers (opportunity cost method) from the wider societal perspective (£, 95% CI)**

|  | **Control group** | | | | **Intervention group** | | | | **Mean change at 6 months between group (Intervention - control)** | **p value** |
| --- | --- | --- | --- | --- | --- | --- | --- | --- | --- | --- |
|  | **Baseline (n=177)** | **3 months (n=140)** | **6 months (n=137)** | **Mean cost difference between baseline and 6 months** | **Baseline (n=175)** | **3 months (n=129)** | **6 months (n=126)** | **Mean cost difference between baseline and 6 months** |  |  |
| Community services (public sector, private and third sector) | £70 (57,83) | £146 (152,230) | £189 (153,228) | £120 (86,159) | £111 (88,135) | £167 (127,211) | £140 (104,179) | £22 (-14,60) | -£98 (47,148) | <0.001* |
| Hospital service (public sector, private and third sector) | £99 (60,146) | £109 (39,210) | £194 (97,318) | £102 (0,231) | £178 (77,328) | £162 (76,265) | £159 (79,259) | £38 (-70,156) | -£64 (-96,238) | 0.57 |
| Informal care costs (Opportunity cost method) | £441 (384,504) | £387 (337,442) | £397 (337,459) | -£24 (-91,44) | £474 (405,546) | £402 (336,475) | £475 (395,564) | £32 (-38,101) | £56 (-150,47) | 0.05* |
| **Total resource use and informal care costs** | **£598 (527,671)** | **£632 (532,748)** | **£750 (609,916)** | **£168 (35,320)** | **£759 (616,952)** | **£726 (596,872)** | **£759 (635,895)** | **£81 (-55,218)** | **-£87 (-120,309)** | **0.23** |

For 95% CI using non-parametric bootstrapping 5,000 samples bias-corrected and accelerated method. *Because cost data is non-normality, Mann–Whitney U independent t test was used with p-value significant level at 0.05.

**Supplementary Table 12. Mean costs per participant of hospital, community and other services for people living with dementia from the wider societal perspective (£, 95% CI)**

|  | **Control group** | | | | **Intervention group** | | | | **Mean change at 6 months between group (Intervention - control)** | **p value** |
| --- | --- | --- | --- | --- | --- | --- | --- | --- | --- | --- |
|  | **Baseline (n=177)** | **3 months (n=138)** | **6 months (n=130)** | **Mean cost difference between baseline and 6 months** | **Baseline (n=175)** | **3 months (n=129)** | **6 months (n=122)** | **Mean cost difference between baseline and 6 months** |  |  |
| **Mean cost of community services (societal perspective)** | £232 (195,271) | £203 (166,242) | £224 (184,267) | -£5 (-52,44) | £260 (222,300) | £232 (185,284) | £244 (191,304) | -£4 (-54,49) | -£1 | 0.23 |
| **Mean cost of hospital services (societal perspective)** | £446 (302,617) | £562 (307,898) | £614 (349,927) | £142 (-169,494) | £369 (250,515) | £388 (228,591) | £577 (345,837) | £161 (-1678,500) | £19 | 0.75 |
| **Mean cost of other services (societal perspective)** | £165 (93,253) | £150 (82,231) | £249 (148,365) | £74 (-40,187) | £249 (156,347) | £300 (170,453) | £277 (157,427) | £67 (-47,183) | -£6 | 0.68 |
| **Total mean resource use: community, hospital, and other services (societal perspective)** | **£836 (647,1047)** | **£915 (646,1258)** | **£1088 (807,1407)** | **£211 (-100,529)** | **£876 (716,1063)** | **£909 (693,1161)** | **£1099 (802,1420)** | **£231 (-120,590)** | **£20** | **0.37** |

For 95% CI using non-parametric bootstrapping 5,000 samples bias-corrected and accelerated method. *Due to non-normality of cost data, Mann–Whitney U independent t test was used with p-value significant level at 0.05.

**Supplementary Table 13. Sensitivity analysis (removing outliers) reporting on the difference in mean costs per participant from the public sector and societal perspective (£, 95% CI)**

|  | **Control group** | | | | **Intervention group** | | | | **Mean change at 6 months between groups (Intervention - control)** | **p value** |
| --- | --- | --- | --- | --- | --- | --- | --- | --- | --- | --- |
|  | **Baseline (n=172)** | **3 months (n=129)** | **6 months (n=137)** | **Mean cost difference between baseline and 6 months** | **Baseline (n=156)** | **3 months (n=129)** | **6 months (n=126)** | **Mean cost difference between baseline and 6 months** |  |  |
| **Mean cost of community services (public sector)** | £58 (48,68) | £99 (81,118) | £145 (119, 175) | £77 (51, 104) | £56 (45,67) | £101 (78,122) | £110 (84,139) | £45 (21,69) | -£33 (-69,4) | 0.06 |
| **Mean cost of hospital services (public sector)** | £0 (0,0) | £0 (0,0) | £27 (18,38) | £20 (11,30) | £0 (0,0) | £0 (0,0) | £29 (18,42) | £26 (15,38) | £6 (-10,23) | 0.38 |
| **Mean total resource use (public sector)** | £56 (47,65) | £74 (58,89) | £123 (98,148) | £67 (43,92) | £54 (44,64) | £70 (53,86) | £89 (70,110) | £36 (15,57) | -£31 (-64,2) | 0.15 |
| **Mean cost of community services (societal perspective)** | £60 (50,70) | £120 (97,144) | £154 (126,184) | £93 (67,125) | £67 (53,81) | £111 (87,136) | £114 (86,141) | £47 (22,72) | -£46 (-85,-7) | 0.018* |
| **Mean cost of hospital services (societal perspective)** | £0 (0,0) | £0 (0,0) | £20 (11,30) | £20 (11,30) | £0 (0,0) | £0 (0,0) | £27 (16,38) | £26 (15,38) | £6 (-10,23) | 0.38 |
| **Mean total resource use costs (societal perspective)** | £53 (41, 65) | £105 (84,129) | £146 (114,178) | £94 (63,126) | £51 (37,65) | £103 (78,126) | £105 (77,136) | £55 (28,84) | -£39 (-82,4) | 0.07 |

For 95% CI using non-parametric bootstrapping 5,000 samples bias-corrected and accelerated method. *Due to non-normality of cost data, Mann–Whitney U independent t test was conducted with p-value significance level at 0.05. The mean cost of resource use from the societal perspective included privately funded and third sector resource use, in addition to public sector resource use.

**Supplementary Table 14. Number of outliers that were removed from sensitivity analysis (n)**

|  | **Control group (n)** | | | **Intervention group (n)** | | |
| --- | --- | --- | --- | --- | --- | --- |
|  | **Baseline** | **3 months** | **6 months** | **Baseline** | **3 months** | **6 months** |
| **Mean cost of community services (public sector)** | 5 | 11 | 6 | 19 | 11 | 4 |
| **Mean cost of hospital services (public sector)** | 42 | 20 | 17 | 45 | 23 | 14 |
| **Mean total resource use (public sector)** | 42 | 20 | 17 | 45 | 23 | 14 |
| **Mean cost of community services (societal perspective)** | 5 | 6 | 5 | 19 | 9 | 4 |
| **Mean cost of hospital services (societal perspective)** | 42 | 20 | 17 | 45 | 23 | 14 |
| **Mean total resource use costs (societal perspective)** | 42 | 20 | 17 | 45 | 23 | 14 |

**Supplementary Table 15: Total group costs of resource use and informal care for carers (public sector and wider societal perspective).**

|  |  | **Control group** | | | | |  | **Intervention group** | | | | |
| --- | --- | --- | --- | --- | --- | --- | --- | --- | --- | --- | --- | --- |
|  | **n** | **Baseline** | n | **3 months** | **n** | **6 months** | **n** | **Baseline** | **n** | **3 months** | **n** | **6 months** |
| Total public sector resource use costs | 177 | £29,515.60 | 140 | £35,736.55 | 137 | £41,371.42 | 175 | £48,631.65 | 137 | £35,078.59 | 126 | £23,384.90 |
| Total privately funded resource use costs (societal perspective) | 177 | £0.00 | 140 | £0.00 | 137 | £72.00 | 175 | £980.00 | 137 | £0.00 | 126 | £785.34 |
| Total third sector resource use costs (societal perspective) | 177 | £350.00 | 140 | £681.25 | 137 | £1,643.75 | 175 | £593.75 | 137 | £493.75 | 126 | £75.00 |
| Total informal care costs: proxy good method (societal perspective) | 172 | £79,356.68 | 138 | £58,531.54 | 127 | £55,154.97 | 174 | £84,523.01 | 128 | £52,879.42 | 122 | £59,445.60 |
| **Total costs** | **£109,222.28** | | **£94,949.34** | | **£98,242.14** | | **£134,728.41** | | **£88,451.75** | | **£83,690.84** | |
| Total resource use costs difference between baseline and 3 months |  | -£14,272.94 |  |  |  |  |  | -£46,276.66 |  |  |  |  |
| Total resource use costs difference between baseline and 6 months |  | -£10,980.14 |  |  |  |  |  | -£51,037.57 |  |  |  |  |
