# Supplementary material for: Cost-consequence analysis of an e-health intervention to reduce distress in dementia carers: results from the iSupport randomised controlled trial
Source: BMJ Open. 2025 May 16;15(5):e095611. doi: 10.1136/bmjopen-2024-095611 (PMC12086930; doi:10.1136/bmjopen-2024-095611)
Supplement: Supplementary file 5 [file bmjopen-2024-095611.reviewer_comments.pdf]

## PEER REVIEW HISTORY

BMJ Open publishes all reviews undertaken for accepted manuscripts. Reviewers are asked to complete a checklist review form and are provided with free text boxes to elaborate on their assessment. These free text comments are reproduced below.

### ARTICLE DETAILS

**Title (Provisional)**

Cost-consequence analysis of an e-health intervention to reduce distress in dementia carers: Results from the iSupport randomised controlled trial

**Authors**

Anthony, Bethany; Doungsong, Kodchawan; Macleod, Catherine; Flynn, Greg; Masterson-Algar, Patricia; Goulden, Nia; Egan, Kieren; Jackson, Kiara; Kurana, Suman; Hughes, Gwenllian; Innes, Ryan; Connaghan, John; Proctor , Danielle; Ismail , Fatene Abakar; Hoare, Zoe; Spector, Aimee; Stott, Joshua; Windle, Gill; Edwards, Rhiannon Tudor

---

### VERSION 1 - REVIEW

---

| | |
|---|---|
| **Reviewer** | **1** |
| **Name** | **Fox, Chris** |
| **Affiliation** | **Norwich Medical School, Department of Psychological** |
| **Sciences** | |
| **Date** | **17-Jan-2025** |
| **COI** | **None** |

I would like a bit more in limitations as population was highly educated and white and those in greatest need may have been missed so this needs to be recognised in the discussion on costs.

---

| | |
|---|---|
| **Reviewer** | **2** |
| **Name** | **Jiang, Xiangxiang** |
| **Affiliation** | **University of South Carolina** |
| **Date** | **05-Feb-2025** |
| **COI** | **None** |

This study appropriately examined the cost-consequence of iSupport in addressing mental health issues among dementia caregivers, utilizing a well-designed approach and thorough

analysis. The findings were clearly presented and detailed. iSupport is a forward-looking program designed for caregivers. I have a few comments for further consideration.

1. It would be beneficial if authors could add the difference test results of baseline characteristics in Table 1.

2. The iSupport RCT found no significant between-group differences in the primary outcome or depression at 3 and 6 months. Is this due to the limited sample size, the intervention duration, or the effectiveness of iSupport itself?

3. From a broader societal perspective, there was no significant difference in mean total resource use or informal care costs. Given this, along with the potential usability challenges of an online platform for older caregivers, is iSupport still a worthwhile intervention to promote?

4. Since this study compares costs at 3 and 6 months, is this timeframe sufficient for iSupport to demonstrate its full effect? What are the potential long-term impacts of the program?

---

| | |
|---|---|
| **Reviewer** | **3** |
| **Name** | **Dack, Kyle** |
| **Affiliation** | **University of Bristol, MRC Integrative Epidemiology Unit** |
| **Date** | **27-Mar-2025** |
| **COI** | **None** |

---

Statistical review summary:

The methods are transparent and valid, and the results are clearly reported. My one concern is that there was a significant difference in baseline costs between the intervention and control group for carers (but less so for people living with dementia). I think this should be explored more to understand the underlying reasons, whether it is outliers, a specific category of costs, it would be informative because it means the study does not have baseline equivalence, and this difference could influence the comparisons at later timepoints.

Detailed comments:

Abstract

1. Usually p-values greater than 0.01 are reported to only two decimal places, so I suggest rounding the 0.229 p-value to 0.23.

2. “Compared with usual care, there was a significant difference (p=0.003) in mean costs of resource use in the intervention group from a public sector perspective” I think the effect size should be reported, because reading the abstract I assumed that costs would be higher in the intervention group. Whereas Table 2 shows that the intervention group had both

lower mean costs at 6 months, and a much greater change in costs compared to baseline (-51k vs +10k).

Introduction

3. “The annual cost of dementia is over £34 billion, and unpaid care accounts for 40% of this cost” It would be helpful to elaborate more on this point, how unpaid care can have a cost. Possibly the opportunity cost, or the estimated equivalent if the care was paid?

4. I think the “study aim” section could be removed because it repeats information in the final paragraph of the introduction.

Methods

5. The beginning of the methods states that this was a multi-centre trial. But the centres themselves – where patients were recruited – are not described. The authors have directed readers to the protocol for details of recruitment, but at least the basics of the study setting should be reported here.

6. I suggest using a separate subheading to describe the intervention/control. It would make it easier for readers to locate.

Results

7. Table 1. “Travel distance from person with dementia”, if approximately 40% of carers were the partner of the person living with dementia, I imagine this means there were a lot of people who reported zero for this item. This could skew the mean, and the median may be more informative?

8. Resource use costs for carers were much higher at baseline for the intervention group than the control group. I see from table 3 this is driven by higher community service and hospital service costs. This is quite surprising and interesting given that participants were randomly assigned to the two groups with otherwise equal baseline characteristics, and the intervention had not begun (?). Is this purely random chance? It seems like it is a study limitation – as the randomization has not equalized all baseline characteristics? I notice that there is less baseline difference in costs for the patients themselves.

This is interesting and quite important because the analysis compares change at 6 months, which is dependent on the initial baseline costs. Did the authors perform any statistical investigation of these differences that could be reported? For example an explanation of the specific costs which were higher, identifying outlier influence, comparing individual-level distributions using histograms, or comparing medians (if the costs are not normally distributed). Some of these could be informative.

9. Resource use cost is reported to 0 d.p. for the mean, but 2 d.p. for the 95% CI. I think this should be made consistent.

Discussion

No suggestions.

---

## VERSION 1 - AUTHOR RESPONSE

| **Editor's Comments to Author:** | **Author response:** |
|---|---|
| **Reviewer 1: Dr. Chris Fox, Norwich Medical School, University of Exeter** | |
| I would like a bit more in limitations as population was highly educated and white and those in greatest need may have been missed so this needs to be recognised in the discussion on costs. | Thank you very much for highlighting this important limitation. We have now added text to acknowledge this in the discussion section of the manuscript to recognise that the sample's homogeneity raises concern about the generalisability of the findings as people with the greatest need for support may have been missed. We have also discussed the implications on patterns of resource use and subsequent estimation of costs. |
| **Reviewer 2: Dr. Xiangxiang Jiang, University of South Carolina** | |
| 1. It would be beneficial if authors could add the difference test results of baseline characteristics in Table 1. | Thank you very much for your feedback. As noted in the CONSORT guidelines, item 15, https://www.bmj.com/content/340/bmj.c869.short comparison of baseline characteristics in individually randomised controlled trials is not recommended. This is because the sample is randomised therefore any observed differences between groups are attributable to chance. Therefore, statistically testing these differences is not meaningful and can mislead readers into assuming there is a systematic bias where there is not. Although the differences in resource use at baseline between groups was down to chance, we have included information on the main cost drivers at baseline. |
| 2. The iSupport RCT found no significant between-group differences in the primary outcome or depression at 3 and 6 months. Is this due to the limited sample size, the intervention duration, or the effectiveness of iSupport itself? | Thank you very much for your helpful comment. We have explained in the discussion section of the manuscript that the null findings are likely due to the effectiveness of the iSupport intervention itself as it stands in its current format. There was a low level of engagement with the iSupport platform over the intervention period and thus the intervention is unlikely to be effective due to the self-guided nature of the support. |
| 3. From a broader societal perspective, there was no significant difference in mean total resource use or informal care costs. Given this, along with the potential usability | Thank you very much for raising this important point. We have now added text to the discussion section of the manuscript to highlight that although the findings reported reductions in public sector |

| | |
|---|---|
| challenges of an online platform for older caregivers, is iSupport still a worthwhile intervention to promote? | costs, the intervention reported no significant impact on costs from the wider societal perspective and given this and the usability issues for older carers using online platforms, promoting iSupport is not justified from a societal viewpoint. |
| 4. Since this study compares costs at 3 and 6 months, is this timeframe sufficient for iSupport to demonstrate its full effect? What are the potential long-term impacts of the program? | Thank you for your helpful feedback. We have now added text in the discussion section to acknowledge that the study assessed costs at 3 and 6 months and therefore does not consider the long-term impact of the intervention. We have also added this as a key point to the 'Strengths and Limitations' section (after the abstract). We have emphasised in the discussion section of the manuscript that the study did not account for the long-term impacts on health and social care resource use costs and the potential for wider societal impacts such as informal care costs. We have further added that the initial impacts on reduced resource use might plateau or diminish over time as participants require ongoing support that was not part of the intervention. |
| **Reviewer 3: Dr. Kyle Dack, University of Bristol** | |
| Statistical review summary:<br>The methods are transparent and valid, and the results are clearly reported. My one concern is that there was a significant difference in baseline costs between the intervention and control group for carers (but less so for people living with dementia). I think this should be explored more to understand the underlying reasons, whether it is outliers, a specific category of costs, it would be informative because it means the study does not have baseline equivalence, and this difference could influence the comparisons at later timepoints. | Thank you very much for your helpful feedback. We have reported that the main cost drivers for the high resource use costs at baseline among the intervention group were the high frequencies of GP visits, support/educational group contacts and inpatient hospital stays reported by carer participants. We descriptively reported the mean cost per participant for the groups at baseline, 3 months and 6 months. The mean change from baseline at 6 months was assessed which reported a statistically significant difference in mean community services costs between groups. |
| Abstract<br>1. Usually p-values greater than 0.01 are reported to only two decimal places, so I suggest rounding the 0.229 p-value to 0.23. | Thank you very much. We have now rounded up the p-value to 0.23. |
| Abstract<br>2. "Compared with usual care, there was a significant difference (p=0.003) in mean costs of resource use in the intervention group from a public sector perspective" I think the effect size should be reported, because reading the abstract I assumed that costs would be higher in the intervention group. Whereas Table 2 shows that the intervention group had both lower mean | Thank you very much for the helpful feedback. We have now reported on the effect size and have amended the text to ensure consistency of reporting throughout the manuscript. We have now amended the text to state that both groups reported higher mean costs between baseline and 6 months, but the mean change in costs were significantly lower in the intervention group. Between baseline and 6 months, the mean change in total resource use costs from the public sector |

| | |
|---|---|
| costs at 6 months, and a much greater change in costs compared to baseline (-51k vs +10k). | perspective was significantly different between groups (p=0.003, r = -0.161) reporting a mean change per participant of £65 (95% CI:-52.52-193.73) for intervention group and £211 (95% CI: 95.67-355.76) for control group. We have also amended the Table 2 header to clarify that Table 2 presents total group costs rather than mean costs (which are presented in Table 3). |
| Introduction<br>3. "The annual cost of dementia is over £34 billion, and unpaid care accounts for 40% of this cost" It would be helpful to elaborate more on this point, how unpaid care can have a cost. Possibly the opportunity cost, or the estimated equivalent if the care was paid? | Thank you very much for this helpful feedback. We have now added text to explain that the figure of 40% of the total annual cost of dementia represents the estimated economic value if the equivalent amount of care was provided by formal (paid) carers. |
| Introduction<br>4. I think the "study aim" section could be removed because it repeats information in the final paragraph of the introduction. | Thank you. We have now removed the 'study aim' section. |
| Methods<br>5. The beginning of the methods states that this was a multi-centre trial. But the centres themselves – where patients were recruited – are not described. The authors have directed readers to the protocol for details of recruitment, but at least the basics of the study setting should be reported here. | Thank you. We have included details of the centres and how the participants were recruited. |
| Methods<br>6. I suggest using a separate subheading to describe the intervention/control. It would make it easier for readers to locate. | Thank you for the suggestion we have now used separate subheadings to describe the intervention and control. |
| Results<br>7. Table 1. "Travel distance from person with dementia", if approximately 40% of carers were the partner of the person living with dementia, I imagine this means there were a lot of people who reported zero for this item. This could skew the mean, and the median may be more informative? | Thank you very much for your helpful feedback. We have now added median and IQR for "Travel distance from person with dementia" in Table 1. |
| 8. Resource use costs for carers were much higher at baseline for the intervention group than the control group. I see from table 3 this is driven by higher community service and hospital service costs. This is quite surprising and interesting given that participants were randomly assigned to the two groups with otherwise equal baseline characteristics, and the intervention had not begun (?). Is this purely random chance? It seems like it is a study limitation – as the | Thank you very much for this very helpful feedback. We have now amended the text to ensure consistency throughout the manuscript and have now used transparent wording to explain that we have reported on the mean change in costs for groups between baseline and 6 months. We acknowledge that there are differences in costs between groups at baseline and we have now added text to report on the specific costs that were higher at baseline. We have also conducted sensitivity analysis to explore the impact of these |

| | |
|---|---|
| randomization has not equalized all baseline characteristics? I notice that there is less baseline difference in costs for the patients themselves.<br>This is interesting and quite important because the analysis compares change at 6 months, which is dependent on the initial baseline costs. Did the authors perform any statistical investigation of these differences that could be reported? For example an explanation of the specific costs which were higher, identifying outlier influence, comparing individual-level distributions using histograms, or comparing medians (if the costs are not normally distributed). Some of these could be informative. | outliers on the results (Supplementary File 2, Tables 13 and 14). |
| 9. Resource use cost is reported to 0 d.p. for the mean, but 2 d.p. for the 95% CI. I think this should be made consistent. | Thank you, we have now reported both to 0 d.p for consistency. |
